# Prolonged heat stress in *Brassica napus* during flowering negatively impacts yield and alters glucosinolate and sugars metabolism

**DOI:** 10.3389/fpls.2025.1507338

**Published:** 2025-05-09

**Authors:** Mariam Kourani, Maria Anastasiadi, John P. Hammond, Fady Mohareb

**Affiliations:** ^1^ The Bioinformatics Group, Centre for Soil, Agrifood and Biosciences (SABS), Cranfield University, Cranfield, United Kingdom; ^2^ School of Agriculture, Policy and Development, University of Reading, Reading, United Kingdom

**Keywords:** *Brassica napus*, oilseed rape, heat stress, RNA-seq, HPLC, LC-MS, simple sugars, glucosinolates

## Abstract

Oilseed rape (*Brassica napus*), one of the most important sources of vegetable oil worldwide, is adversely impacted by heatwave-induced temperature stress especially during its yield-determining reproductive stages. However, the underlying molecular and biochemical mechanisms are still poorly understood. In this study, we investigated the transcriptomic and metabolomic responses to heat stress in *B. napus* plants exposed to a gradual increase in temperature reaching 30°C in the day and 24°C at night for a period of 6 days. High-performance liquid chromatography (HPLC) and liquid chromatography–mass spectrometry (LC-MS) was used to quantify the content of carbohydrates and glucosinolates, respectively. Results showed that heat stress reduced yield and altered oil composition. Heat stress also increased the content of carbohydrate (glucose, fructose, and sucrose) and aliphatic glucosinolates (gluconapin and progoitrin) in the leaves but decreased the content of the indolic glucosinolate (glucobrassicin). RNA-Seq analysis of flower buds showed a total of 1,892, 3,253, and 4,553 differentially expressed genes at 0, 1, and 2 days after treatment (DAT) and 4,165 and 1,713 at 1 and 7 days of recovery (DOR), respectively. Heat treatment resulted in downregulation of genes involved in respiratory metabolism, namely, glycolysis, pentose phosphate pathway, citrate cycle, and oxidative phosphorylation especially after 48 h of heat stress. Other downregulated genes mapped to sugar transporters, nitrogen transport and storage, cell wall modification, and methylation. In contrast, upregulated genes mapped to small heat shock proteins (sHSP20) and other heat shock factors that play important roles in thermotolerance. Furthermore, two genes were chosen from the pathways involved in the heat stress response to further examine their expression using real-time RT-qPCR. The global transcriptome profiling, integrated with the metabolic analysis in the study, shed the light on key genes and metabolic pathways impacted and responded to abiotic stresses exhibited as a result of exposure to heat waves during flowering. DEGs and metabolites identified through this study could serve as important biomarkers for breeding programs to select cultivars with stronger resistance to heat. In particular, these biomarkers can form targets for various crop breeding and improvement techniques such as marker-assisted selection.

## 1 Introduction

Over the last decade, climate change has led to more extreme climatic events, impacting crop productivity and threatening global food security (Ismaili et al., 2015; Lamaoui et al., 2018). These extreme events include periods of high temperature stress in the form of heatwaves (Goel et al., 2023), broadly defined as periods of excessively high temperature as compared to the local climate (Dikšaitytė et al., 2019). Previous studies have shown that heat stress can be detrimental during reproduction development due to the damage caused to plant organs and cellular structures (Hedhly, 2011; Hinojosa et al., 2019; Goel et al., 2023). During reproduction, high temperature can induce irreversible structural and physiological changes in both male and female floral organs, leading to premature senescence (Lohani et al., 2020; Jagadish et al., 2021). High temperature stress can also decrease chlorophyll synthesis and disrupt photosynthesis and respiration, further reducing the yield potential of crop plants (Hasanuzzaman et al., 2014; Zandalinas et al., 2016). Thus, understanding the impact of heatwaves on crops and particularly during reproductive stages is essential to the development of varieties that can withstand these periods of elevated temperatures.

Traditionally, most heat stress experiments involve a heat shock, where plants are subjected to a high temperature within a very short time (10–15°C above their optimal temperature, from several minutes to a few hours) (Wahid et al., 2007). As a result, plant responses to heat shock have been well studied (Huang et al., 2019; Wang et al., 2020), but relatively little information exists on the responses to heatwaves, especially at the transcriptomic and metabolomic levels (Jin et al., 2011; Glaubitz et al., 2017).

In plants, heat shock reduces photosynthesis and respiratory metabolism and increases antioxidant activity (Huang et al., 2019; Wang et al., 2020). Heat shock also negatively affects plant growth and productivity (Ismaili et al., 2015). While these experiments revealed the regulatory mechanisms in response to sudden heat stress (Seth et al., 2021; Ikram et al., 2022; Li et al., 2022), they do not fully represent the impact of temperature changes in the field during heatwave conditions. As a result, several studies employed prolonged warming experiments to study heat stress (Jin et al., 2011; Way and Yamori, 2014; Glaubitz et al., 2017). In *Arabidopsis*, studies showed that plants exhibit different response patterns to prolonged warming (7 days) as compared to heat shocks (Jin et al., 2011; Wang et al., 2020). While prolonged warming led to a reduction in stomatal conductance, heat shock increased transpiration. Under both heat shock and prolonged heat stress, there was an induction of antioxidant enzymes in *Arabidopsis*; however, these were significantly higher under the heat shock treatment compared to the prolonged heat treatment (Wang et al., 2020).

Oilseed rape (*Brassica napus* L.) is the second largest source of oilseed after soybean and the third largest source of vegetable oil worldwide (USDA, 2025). Cultivation and breeding practices have resulted in numerous genetically diverse lines with strong agronomic and adaptation traits (Sun et al., 2017). As a result, *B. napus* is widely cultivated around the world for food, biofuel, and animal feed (Namazkar et al., 2016). Like other major temperate field crops, *B. napus* can be extremely sensitive to high temperature stress, especially if it occurs during flowering or seed development, threatening yields and quality (Angadi et al., 2000; Koscielny et al., 2018; Kourani et al., 2022). Studies in different *Brassica* species have found negative relationships between heat stress and seed yield and quality (Yu et al., 2014). For example, under elevated temperature scenarios, four *B. napus* cultivars experienced a significant reduction in seed biomass, resulting in a 58% decrease in the oil yield and 77% decrease in the essential fatty acid C18:3-ω3 (Namazkar et al., 2016). Heat stress also reduced oil seed content and impaired carbohydrate incorporation into triacylglycerols in *B. napus* (Huang et al., 2019). Additionally, impairment of chlorophyll biosynthesis and disruption of the biochemical reactions of photosystems were exhibited in 10-day-old *B. napus* seedlings subjected to 38°C (Hasanuzzaman et al., 2014). This was manifested through a significant reduction in chlorophyll and leaf relative water content and through an inefficient antioxidant defence system (Hasanuzzaman et al., 2014). Despite the significant advances that these studies have made in understanding the effect of heat stress on different *Brassica* species, most of the research have focused on the seedling stage or grain filling. However, the impact of heatwaves on the transcriptomics and metabolomics of *B. napus*, particularly during its yield determining reproductive stages, is still poorly understood (Kourani et al., 2022).

The aim of this study was therefore to investigate the transcriptomic and metabolomic responses to heat stress in *B. napus* during flowering stage under simulated field conditions. To achieve this, a heat treatment experiment simulating heatwave episode of *B. napus* cv. Westar was conducted in a controlled environment. Global transcriptome profiling using RNA-Seq was employed to identify differentially expressed genes in flower buds of heat stressed plants (30°C/24°C day/night for a period of 6 days). This was complemented with HPLC and LC-MS analyses to detect changes in sugars and glucosinolates (GLS) concentration in leaves in response to the heat treatment. Therefore, this study aims to provide insights into the impact of a future warmer climate on the important oil crop species *B. napus* during its reproductive stages.

## 2 Materials and methods

### 2.1 Plant materials and physiological parameters

Seeds of spring oilseed rape (*Brassica napus* L., cv. Westar) were surface-sterilised and sown into seed trays filled with a seed potting mix (Clover Peat, Dungannon, Northern Ireland). Trays were then placed in polyethylene tunnels for 4 weeks at the Crops and Environment Laboratory, University of Reading, to germinate. Twenty-eight days after sowing (DAS), the plants were transplanted into 3-L pots containing a peat based potting mix (Clover Peat).

At green bud stage (38 DAS), the plants were moved to two controlled environment chambers [Fitotron, Weiss Technik (UK) Ltd], for a period of 1 week to adjust to the new environment before the start of the heat experiment. Each cabinet contained 16 plants and was maintained under control conditions of 20°C/14°C day/night and a photoperiod of 16/8-h light/dark.

### 2.2 Heatwave experimental design

Since the flowering stage of winter-sown *B. napus* occurs during May in the UK, an analysis of daily weather temperatures during this developmentally crucial period was carried out using the UK Meteorological (Met) Office weather data obtained from the Met office (n.d.) and from WeatherOnline (n.d.) platforms. The data showed an increase in the frequency of high temperature fluctuations during May, ranging between 26°C and 28°C over several days (**Supplementary Figure S1**). Given the continuous rise in global temperature, it is expected that future heatwaves would increase in severity and duration (Seneviratne et al., 2012). Based on these data, the current study temperature simulates a heat stress event through a temperature increase to 30°C/24°C day/night for a period of 6 days and 20°C/14°C day/night for control. Forty-five DAS, the temperature in one cabinet was kept under control conditions (20°C/14°C) and denoted as control cabinet, while the second cabinet was set to a gradual increase in temperature and denoted as heat treatment (HT) cabinet. To mimic a heatwave, the temperature in the HT cabinet was increased gradually from 20°C to 30°C between 9:00 and 12:00 in three steps: 20°C at 9:00 h, 24°C at 10:00 h, 28°C at 11:00 h, and 30°C at 12:00 h. The temperature was held at 30°C until 22:00 h (**Supplementary Figure S2B**), before it was dropped gradually and maintained at 24°C until 8:00 of the next day (**Supplementary Figure S2B**). This cycle of gradual increase (day) and decrease (night) of temperature was held for 5 days (**Supplementary Figure S2C**). On the sixth day of treatment, the temperature was gradually decreased to 20°C/14°C day/night cycle (**Supplementary Figure S2D**) and held for a recovery period of 7 days (**Supplementary Figure S2E**). During heat treatment, the plants were frequently irrigated to avoid drought.

### 2.3 Sample preparation for transcriptomic analysis

Five biological replicates from each treatment condition were collected at days 0, 1, 2, 6, and 12 of the experiment. Each replicate was made up of three individual plants. Three flower buds were collected per plant (nine buds per replicate). All samples were immediately snap frozen in liquid nitrogen before they were stored at −80°C. Bud samples were ground into fine powder using pestle and mortar, with the frequent addition of liquid nitrogen to stop enzymatic reactions. The ground material was then stored at −80°C until RNA extraction.

### 2.4 RNA extraction and sequencing

Total RNA from bud samples was extracted using the Spectrum™ Plant Total RNA Kit (Sigma-Aldrich Dorest, UK) in accordance with the manufacturer’s protocol and treated with genomic DNAse using DNASE 70-On Column DNase I Digestion set (Sigma-Aldrich) to eliminate DNA contamination. RNA quantification was estimated on both NanoDrop 2000 (Thermo Scientific) and Qubit 2.0 fluorometer (Invitrogen, USA), and its quality was evaluated on 1% (w/v) denaturing formaldehyde agarose gel (MOPS). Samples were shipped over dry ice to Novogene Europe, where sequencing was performed on the Illumina Novaseq™ 6,000 (PE150) platform.

### 2.5 Differential expression and cluster analysis

Raw sequence reads were assessed for quality using FastQC tools (v0.11.5) (Andrews, 2012). The mean sequence lengths were 150 bp, and the mean sequence GC content was 43%. The mean quality scores in each base position were higher than 36, and the mean quality scores per sequence were 36. As a result, sequence trimming was not necessary.

STAR software (v2.7.10a) was used to map the clean reads to the *B. napus* cv. Westar v0 reference genome (Song et al., 2020), and the percentage of aligned reads was calculated by using the flagstat command from samtools on each alignment file generated by STAR aligner (**Supplementary Figure S3**). To estimate transcript abundance, RNA-Seq by Expectation-Maximisation (RSEM) software (v1.2.18) was used in three steps to prepare the reference, calculate expression, and generate the count matrices. First, rsem-prepare-reference script was used with “–gff3” option to extract reference sequences from the genome and “—star” option to build STAR indices. Next, rsem-calculate-expression script was used with “–paired-end” option to align input reads against the reference transcriptome with STAR and calculated expression values using the alignments. Finally, rsem-generate-data-matrix script was used to generate the count matrices from expression results files.

For differential expression analysis, DESeq2 (v1.41.10) was used in R environment (Love et al., 2014) with default parameters. A pre-filtering step was performed to keep transcripts that have a minimum count of 10 reads in a minimum of four samples. To identify transcripts that were significantly differentially expressed, the two conditions (heat stress versus control) were contrasted in pairwise comparisons. Thresholds of |log2(foldchange)| ≥ 0.5 and adjusted p-value < 0.05 (using the Benjamini and Hochberg method) were considered to identify significantly differentially expressed transcripts between the two treatment conditions.

Significant DEGs in each contrast were further analysed using K-mean clustering based on the kmeans() function in R (distance: Euclidean), with log2FC as the input.

### 2.6 Gene Ontology and KEGG pathway enrichment analysis of DEGs

Gene Ontology (GO) and Kyoto Encyclopedia of Genes and Genomes (KEGG) enrichment analyses were conducted on the DEGs using OmicsBox (BioBam Bioinformatics, 2019). The significantly enriched GO terms and biological pathways in HT samples as compared to control at different timepoints were identified to determine heat-stress-related functions and pathways. The analysis was carried out using Fisher exact statistical test with FDR adjustment cutoff <0.05. The background dataset consisted of all *B. napus* cv. Westar identifiers present in the assembly’s annotation file (Song et al., 2020).

### 2.7 Validation of RNA-seq results by real-time quantitative PCR

Sucrose Synthase 5 (SS5) and Heat Shock Protein 20 (HSP20) were chosen from the pathways that were involved in the heat stress response to further examine their expression in real time using real-time quantitative PCR (RT-qPCR). Five timepoints were chosen for each gene to represent the time course of the experiment. SS5 and HSP20 were not analysed at 1 day after treatment (DAT), as they exhibited similar expression profile at 0 and 1 DAT; therefore, one timepoint was selected (**Supplementary Table S2**).

Total RNA used for transcriptomic sequencing was used for cDNA synthesis. First, cDNA was synthesised from 1 µg total RNA by Invi trogen SuperScript IV VILO Master Mix kit according to the manufacturer’s instructions (Thermo Fisher Scientific). The RT-qPCR reaction volume was 20 µL consisting of 3 µL cDNA, 10 µL SYBR Premix Ex Taq II (Takara, China), 1 µL (200 nM final concentration) of 4 µM of each primer (forward and reverse, **Supplementary Table S3**), and 5 µL RNAse-free water. Three technical and three biological replicates for each sample were measured with the following protocol: 50°C for 2 min, 95°C for 2 min, followed by 40 cycles of 95°C for 3 s, 60°C for 30 s, followed by dissociation curve analysis ramping from 60°C to 95°C with a ramp rate of 0.3°C s^−1^ on an ABI StepOne Plus RT-qPCR platform (Applied Biosystems, USA). BnaActin was used as housekeeping gene to normalise the data. The relative expression level of all selected genes at each timepoint was calculated using the 2^−ΔΔCT^ method of the StepOne software (v2.2.2).

### 2.8 Sample preparation for metabolomic analysis

Five biological replicates from each treatment condition were collected at days 0, 1, 2, 5, 6, 8, and 12 of the heat treatment experiment. Each replicate was made up of three youngest fully expanded leaves collected from three plants. All leaf samples were immediately snap frozen in liquid nitrogen before being stored at −80°C until further analysis. Frozen leaf samples were freeze-dried for 5 days, then ground into a fine powder using a Precellys 24 lysis and homogenisation (Stretton Scientific, Alfreton, UK) and stored at −40°C in sealed sample bags until extraction and analysis.

### 2.9 Glucosinolates extraction and analysis

GLS extraction was carried out as per the protocol of Bell et al. (2015) as follows: 40 mg of ground leaf powder was heated in a dry block at 75°C for 2 min. This step was done as a precautionary measure to inactivate as much myrosinase enzyme as possible before extraction (Pasini et al., 2012). Afterwards, 1 mL of preheated (70% v/v) methanol (70°C) was added to each sample and placed in a water bath for 20 min at 70°C. Samples were then centrifuged for 5 min (6,000 rpm at 18°C) to collect the loose material into a pellet. The supernatant was then transferred into fresh labelled Eppendorf tube and stored at −80°C until further analysis. Before LC-MS analysis, the samples were filtered using 0.25-µm filter discs, diluted in 50% methanol at a 1:4 ratio (dilution factor =5) and spiked with 100 µL of the internal standard Sinigrin (100 ng mL^−1^). The GLS content was analysed by SCIEX QTRAP 6500+ LC-MS.

For LC separation, a Waters Acquity BEH C18 column (particle size, 1.7 µ, 2.1 × 50 mm) with a Security VanGuard system from Waters (UK) was used. The mobile phase consisted of water with 0.1% formic acid (A) and methanol with 0.1% formic acid (B). The gradient started at 5% B and was raised to 90% B in 3.5 min, held at 90% B for 0.5 min and re-equilibrated at 5% B for 1 min. The total time of the gradient program was 5 min. The flowrate was 0.4 mL min^−1^ with a column temperature of 60°C. A 1-µL aliquot of sample was injected for analysis.

The LC system was interfaced with a SCIEX QTRAP 6500+ mass spectrometer equipped with an IonDrive Turbo V ion source. Multiquant software was used to control sample acquisition and data analysis. The QTRAP 6500+ mass spectrometer was tuned and calibrated according to the manufacturer’s recommendations. For quantification, an external standard calibration curve was prepared using a series of standard samples containing the following GLS: Gluconapin (GNA), Progoitrin (PRO) and Glucobrassicin (GBS), with concentrations of 1,000, 500, 250, 100, 50, 10, and 1 ng mL^−1^. Two-way mixed ANOVA in R was performed at 0.05 significance level to calculate the significance variation in concentration under heat treatment as compared to control.

### 2.10 Sugars content extraction and analysis

The extraction of soluble sugars (glucose, fructose, and sucrose) was performed as follows: 50 mg of ground leaf powder was extracted with 1 mL of 62.5% methanol/37.5% HPLC-grade water (v/v) at 55°C over a period of 15 min in a shaking water bath and vortexed every 5 min. Afterwards, the samples were allowed to cool for 2 min and then centrifuged at 13,000 rpm for 10 min and filtered using 0.25-µm filter discs. The supernatant was then transferred to a clean labelled Eppendorf tube and stored at −80°C until further analysis. Before HPLC analysis, the samples were diluted in HPLC-grade water in a 1:2 ratio (dilution factor = 3). Chromatographic separation of sugars content was performed using HPLC (Agilent 1260, Infinity Series) equipped with an Evaporative Light Scattering Detector (ELSD) system and an Asahipak NH2P-50 4E column (250 × 4.6 mm; Shodex, Tokyo, Japan) in an isocratic elution mode. The mobile phase consisted of 75% acetonitrile/25% water at a flowrate of 1 mL min^−1^, a column temperature of 40°C, and an injection volume of 20 µL. The analysis had a total run time of 25 min. For quantification, an external standard calibration curve was prepared using a series of standard samples containing glucose, fructose, and sucrose with the concentrations: 0.025, 0.05, 0.1, 0.5, and 1 mg mL^−1^.

### 2.11 Near-infrared spectroscopy scanning

Following the treatments, the plants were allowed to complete their lifecycle, and total aboveground biomass and seed yields were collected from individual plants. Seed samples were scanned with a DA 7250 NIR analyser (PerkinElmer, Beaconsfield, UK). The seed samples were scanned for moisture, oil, and protein content and for fatty acid composition of the oil.

### 2.12 Statistical analysis

Two-way mixed ANOVA was performed at 0.05 significance level to calculate the statistically significant differences between the means of sugars and glucosinolate concentration under heat treatment as compared to control. For a significant two-way interaction between treatment and timepoint, the following analysis were conducted: the simple main effects of treatment on sugars concentration were examined at each timepoint, using Bonferroni-adjusted p-values for multiple comparisons. This was followed by pairwise comparisons between treatment levels (heat stress vs. control) at each timepoint to determine whether the mean sugars concentrations differed significantly between heat stress and control. In addition, near-infrared spectroscopy (NIRS) data were analysed for the investigated components using a t-test. All statistical analysis was performed using R.

## 3 Results

### 3.1 Prolonged heat exposure reduced yield and altered *B. napus* oil composition

**Figure 1:**
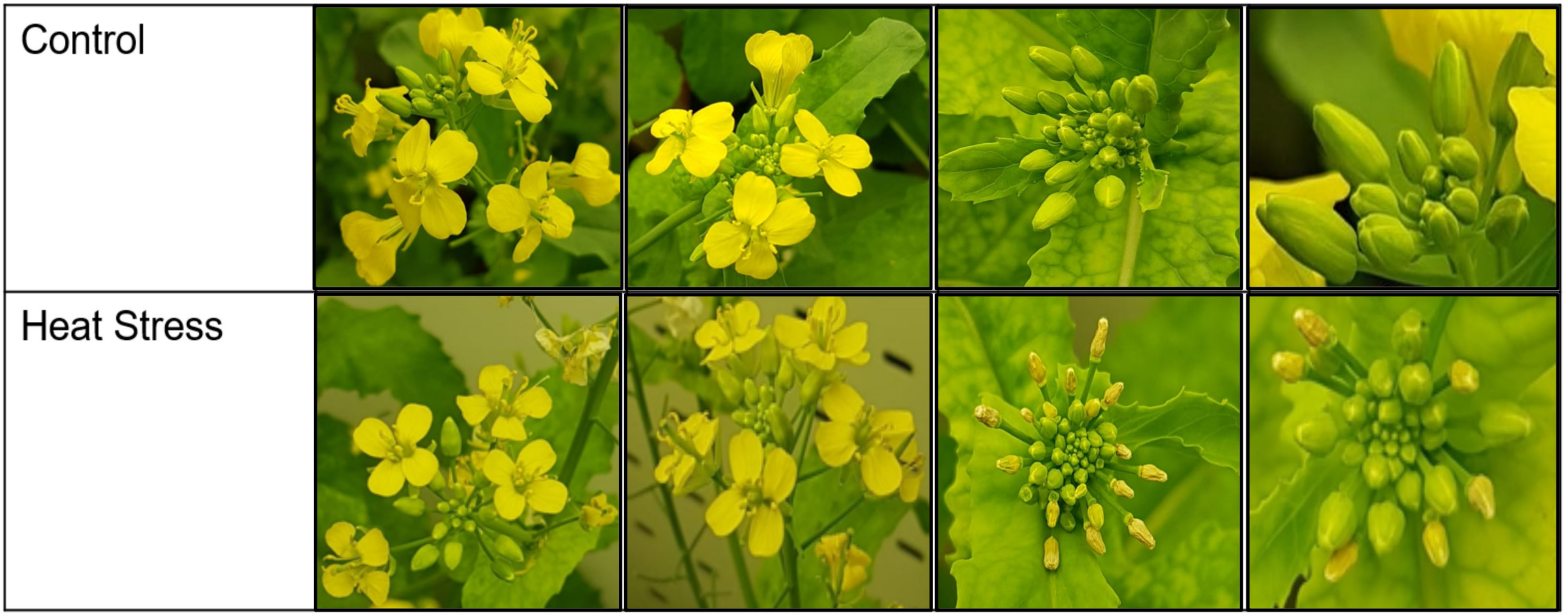
*B. napus* flowers and buds at 5 DAT and 3 DOR, respectively.

Heat treatment significantly altered plant growth and appearance, with visible physiological changes on the flowers and buds (**Figure 1**). At 5 DAT, flowers displayed smaller and paler petals compared to plants growing under control conditions. At 3 days of recovery (DOR), many buds suffered from abscission, appeared brown, and shrunken in size on plants subjected to the heat treatment. At maturity, heat-treated plants exhibited less total biomass and total seed weight relative to control plants (**Supplementary Figure S4**). In terms of oil quality, heat treatment significantly decreased linoleic acid, linolenic acid, and palmitic acid concentration but increased oleic acid and stearic acid concentration in seeds compared to control plants (**Supplementary Figure S5**).

### 3.2 Differential gene expression identifies distinct patterns of expression in *B. napus* under heat stress and recovery

Libraries were constructed and sequenced on the Illumina NovaSeq platform. An average of 47 million reads were generated per sample after low-quality reads were filtered out (**Supplementary Table S1**). Following this cleaning step, approximately 90%–95.49% total reads per sample were mapped to the *B. napus* cv. Westar v0 reference genome (**Supplementary Figure S3**) (Song et al., 2020). Principle component analysis (PCA) was used to analyse the samples that drove the group separation (**Supplementary Figure S7**). PC1 and PC2 captured 48.9% and 8.2% of the total variance of the samples, respectively. The analysis showed that both control and treatment samples clustered near each other at 7 DOR, which demonstrated that heat-treated plants might have started to return to normal after 7 days of recovery.

To understand the effect of heat stress on gene expression in *B. napus*, differentially expressed genes (DEGs) were identified in a pairwise comparison between heat treatment and control conditions at five timepoints during and after heat treatment. A total of 1,892, 3,253, 4,553, 4,165, and 1,713 genes were differentially expressed at days 0, 1, and 2 DAT and 1 and 7 DOR, respectively (**Supplementary Figure S8**). Analysis of the top differentially expressed genes after 24 h of heat treatment revealed that eight transcripts with logFC ranging between 4.4 and 10.8 mapped to small heat shock proteins (sHSP20), in addition to transcripts mapped to other heat shock factors such as Elongation Factor 1-beta 1-like and Chaperone Protein ClpB1 (**Supplementary Table S7**). On the other hand, top downregulated genes featured transcripts mapped to sugars transporters, nitrogen transport and storage, cell wall modification, and methylation (**Supplementary Table S8**). Similarly, after 48 h of heat treatment, many of the identified transcripts remained among the top DE, both up- and downregulated, highlighting the significant effect of heat stress on the functions performed by these enzymes and proteins.

**Figure 2:**
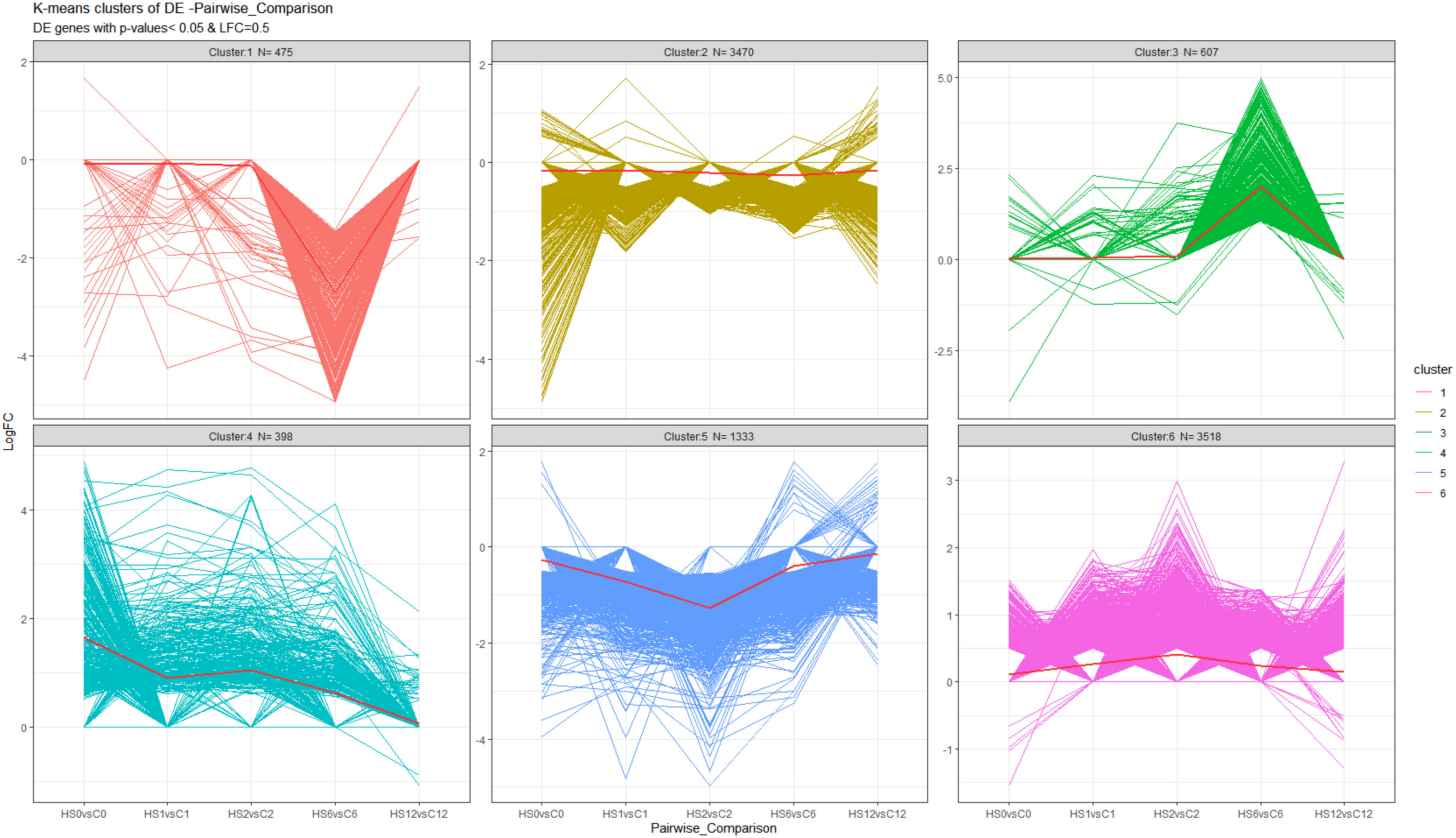
K-means cluster analysis of differential expressed genes in *B. napus* during and after heat treatment. K-means clustering was applied to 9,933 DE genes with logFC ≥ 0.5 using the “kmeans” function in R package “stats” (v. 3.2.2), where k represents a pre-specified number of clusters. Six groups of genes were classified, with N the number of genes in each cluster, and the solid red lines in each cluster indicate mean changes in DEG expression.

To identify trends in the expression of genes across the time course of the experiment, K-means clustering was applied to 9,933 DE genes with logFC ≥ 0.5. The algorithm randomly assigned each gene into one of the clusters based on the Euclidean distance between the gene and the cluster mean. Six distinctive clusters based on expression changes across the five timepoints were identified (**Figure 2**). Interestingly, clusters 1 and 3 (475 and 607 genes), showed opposite expression patterns towards the end of the heat treatment and the start of the recovery phase. In cluster 1, the expression profile of genes decreased at 1 DOR (HS6vsC6), before returning to normal levels at 7 DOR (HS12vsC12), while in cluster 3, a sharp increase in expression was observed at 1 DOR (HS6vsC6), which returned to normal levels at 7 DOR (HS12vsC12).

In clusters 2 and 5 (3,470 and 1,333 genes), genes were downregulated along the course of the experiment but more pronounced during the heat treatment phase especially in cluster 5, which exhibited a greater decrease in expression. Both clusters 4 and 6 (398 and 3,518 genes) showed increased expression during the treatment before it started to decline towards the end, reaching normal levels at 7 DOR (HS12vsC12) (**Figure 2**).

### 3.3 Validation of RNA-seq data by quantitative real-time PCR

To verify the reliability of RNA sequencing results, two genes with diverse expression profiles, including upregulated or downregulated at different timepoints of the experiment, were selected for real-time qPCR to measure expression levels. As a result, Heat Shock Protein 20 (HSP20) and Sucrose Synthase 5 (SS5), exhibited similar expression profiles between RT-qPCR and RNA-seq data (**Supplementary Figure S9**, **Supplementary Table S4**), with correlation coefficients of r=0.86 and r=0.68, respectively.

### 3.4 Functional annotation of DEGs identifies key pathways responding to heat treatment and recovery

Gene Ontology (GO) enrichment analysis of the DEGs was performed to identify enriched GO terms. Terms such as “binding,” “catalytic activity,” “metabolic process,” “hydrolase activity,” and “ion binding” were among the predominant enriched terms at all timepoints with more transcripts mapped to these terms during heat treatment than during recovery (**Supplementary Figures S10**-**S14**). Oxidoreductase, hydrolase, and transferase were among the top identified enzymes (**Supplementary Figure S15**). These results suggest that high temperature activates several metabolic processes through the expression of numerous enzymes involved in alleviating the impact of heat stress not only during the stress period but also during recovery.

Based on Kyoto Encyclopedia of Genes and Genomes (KEGG) enrichment analysis (Kanehisa and Goto, 2000), pathways in the respiratory metabolism, namely, glycolysis and pentose phosphate pathway were enriched in all heat treatment comparisons at all timepoints, while citrate cycle and oxidative phosphorylation were enriched at 2 DAT and 1 DOR, respectively (**Supplementary Figures S16**-**S19**). Other related pathways were also enriched along the course of the treatment and during recovery period, and these include one carbon pool by folate (enriched at 1 and 2 DAT and 1 DOR), carbon fixation in photosynthetic organisms, cysteine and methionine metabolism (enriched at 1 and 2 DAT), glycerolipid and tryptophan metabolism (enriched at 2 DAT), fatty acid biosynthesis and pyruvate metabolism (enriched at 1 DOR), and fructose and mannose metabolism (enriched at 7 DOR). In the present study, most of the genes involved in these pathways were responsive to high temperature stress, with their expression declining with the onset of heat treatment, especially after 48 h (**Supplementary Figures S20**-**S27**).

#### 3.4.1 Heat treatment downregulated the transcript levels of most aliphatic and indolic GLSs synthetic genes

**Figure 3:**
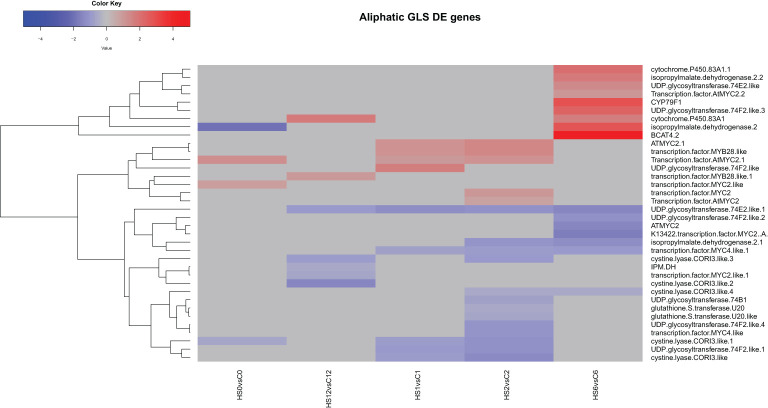
Heatmap showing differential expression of genes involved in the regulation or biosynthesis of aliphatic glucosinolates (GLS) in *Brassica napus*. A total of 13 genes encoding different GLS synthetic genes including different UDP glycosyltransferases and glutathione S-transferases were downregulated, while five genes encoding GLS-related transcription factors were upregulated at 2 DAT.

Analysis of the aliphatic GLS synthetic and regulatory genes showed that heat treatment had a significant effect on the expression of 18 genes at 2 DAT (**Figure 3**). A total of 13 genes encoding different GLS synthetic genes including different UDP glycosyltransferases and glutathione S-transferases were downregulated, while five genes encoding GLS-related transcription factors were upregulated. At 1 DOR, which marks 24 h of recovery, nine genes that were not differentially expressed under heat treatment were found upregulated. These genes include different cytochrome P450 genes, transcription factors, branched-chain aminotransferase 4.2, and isopropylmalate dehydrogenase 2.

**Figure 4:**
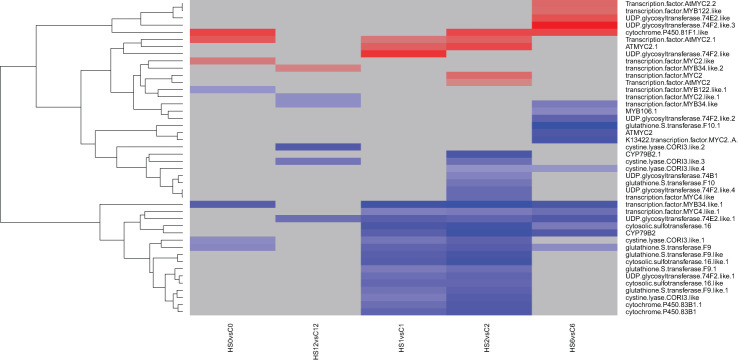
Heatmap showing differential expression of genes involved in the regulation or biosynthesis of indolic glucosinolates (GLS) in *Brassica napus*. A total of 16 and 23 out of 44 genes encoding different sulfotransferases, cytochrome P450 genes, UDP glycosyltransferases, glutathione S-transferases, and transcription factors were downregulated at 1 and 2 DAT, respectively.

Analysis of the indolic GLS synthetic and regulatory genes showed that heat treatment altered the expression of 16 and 23 out of 44 genes at 1 and 2 DAT, respectively (**Figure 4**). These genes include sulfotransferases, cytochrome P450 genes, UDP glycosyltransferases, glutathione S-transferases, and transcription factors. After 1 week recovery (7 DOR), most of the differentially expressed genes identified during heat treatment were no longer differentially expressed. This is similar to what was seen with the aliphatic GLS encoding genes. Interestingly, one transcript encoding UDP glycosyltransferase 74E2-like remained downregulated after 1 week of recovery.

#### 3.4.2 Heat treatment downregulated the transcript levels of most sulphur assimilation and transport genes

**Figure 5:**
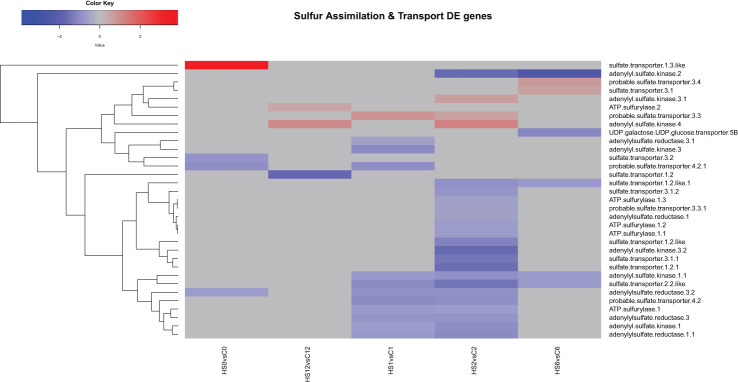
Heatmap showing differential expression of genes involved in the assimilation and transport of sulphur in *Brassica napus*. A total of 20 transcripts were downregulated in response to heat at either 1 or 2 DAT or both.

Analysis of the genes encoding sulphur assimilation and transport during heat treatment showed that 20 transcripts were downregulated in response to heat at either 1 or 2 DAT or both (**Figure 5**). One transcript encoding sulphate transporter was upregulated at 1 and 2 DAT, while two transcripts encoding adenylyl sulphate kinase was upregulated at 2 DAT only. Interestingly, after the end of heat treatment, only five transcripts remained downregulated at 1 DOR, and two transcripts encoding adenylyl sulphate kinase and ATP sulfurylase were upregulated at 7 DOR.

#### 3.4.3 Metabolic analysis revealed altered leaf concentration of aliphatic and indolic GLS in response to heat treatment

**Figure 6:**
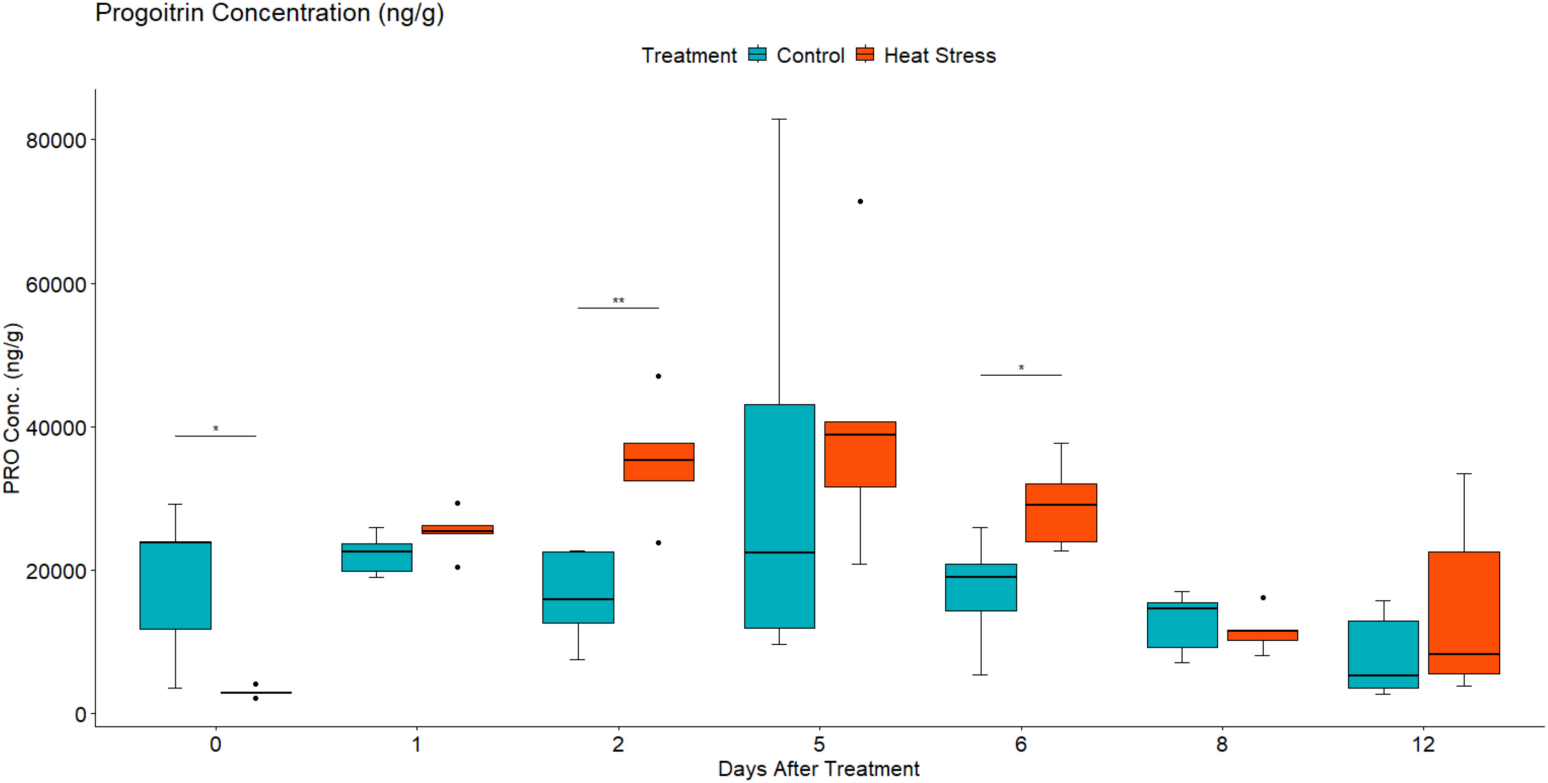
Leaf progroitin (PRO) concentration in ng/g of dry weight (DW) in *Brassica napus* plants subjected to heat treatment (30°C/24°C day/night) at 0–5 DAT (red) or maintained under control temperatures (20°C/14°C day/night) at all timepoints (blue). GLS concentrations were quantified using LC-MS. Data were analysed using two-way mixed ANOVA pairwise comparisons. Each box plot represents data from five replicates, showing the median (horizontal bar), 25th and 75th percentiles (the box), and the min and max values (whiskers), with outliers as dots. Significant differences between heat-treated and control plants at each timepoint are denoted by (*) for p-value ≤ 0.05 or (**) for p-value ≤ 0.01. Heat treatment significantly increased leaf concentration of aliphatic GLS Progoitrin.

**Figure 7:**
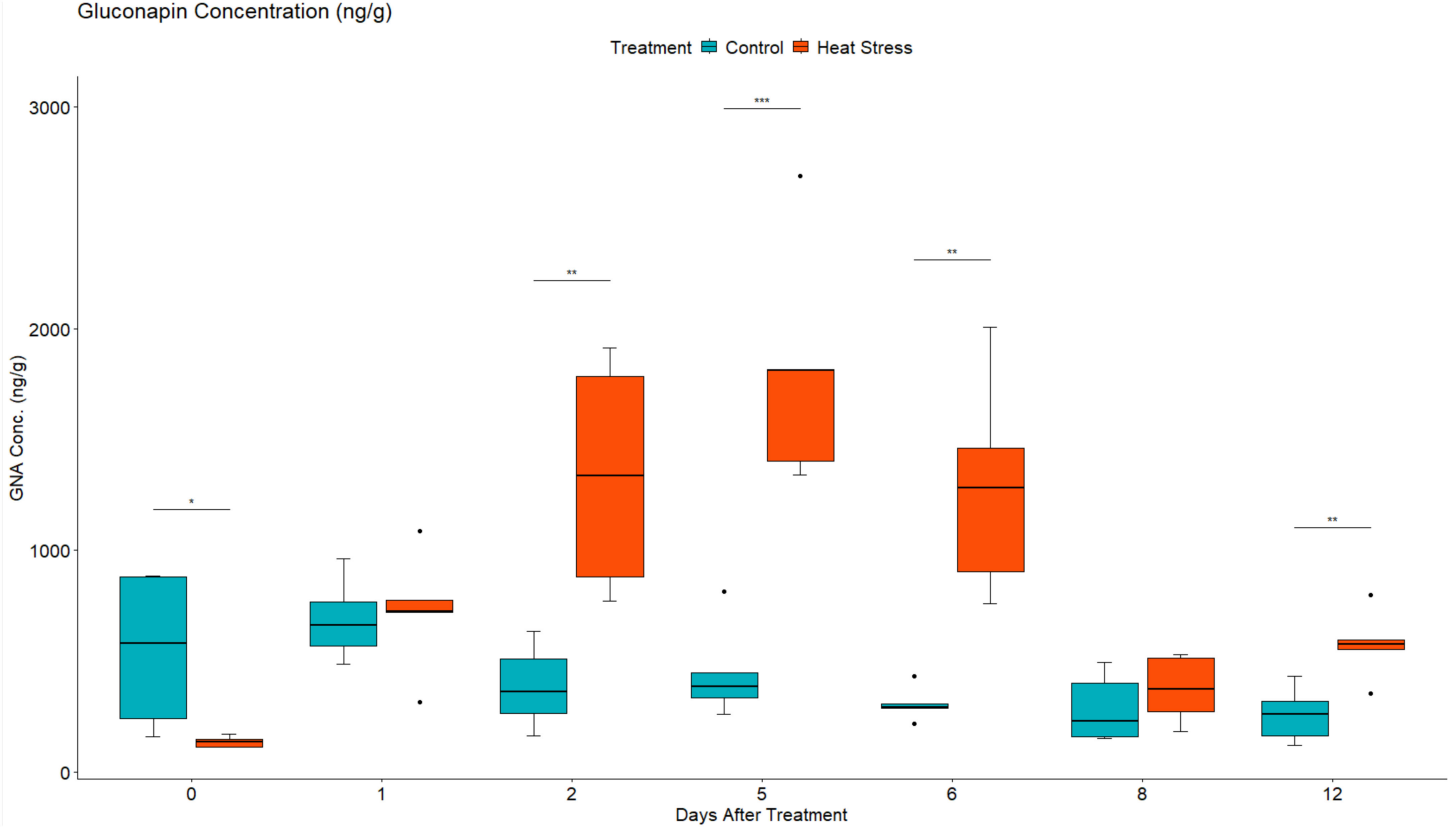
Leaf gluconapin (GNA) concentration in ng/g of dry weight (DW) in *Brassica napus* plants subjected to heat treatment (30°C/24°C day/night) at 0–5 DAT (red) or maintained under control temperatures (20°C/14°C day/night) at all timepoints (blue). GLS concentrations were quantified using LC-MS. Data were analysed using two-way mixed ANOVA pairwise comparisons. Each box plot represents data from five replicates, showing the median (horizontal bar), 25th and 75th percentiles (the box), and the min and max values (whiskers), with outliers as dots. Significant differences between heat-treated and control plants at each timepoint are denoted by (*) for p-value ≤ 0.05 or (**) for p-value ≤ 0.01 or (***) for p-value ≤ 0.001. Heat treatment significantly increased leaf concentration of aliphatic GLS gluconapin.

**Figure 8:**
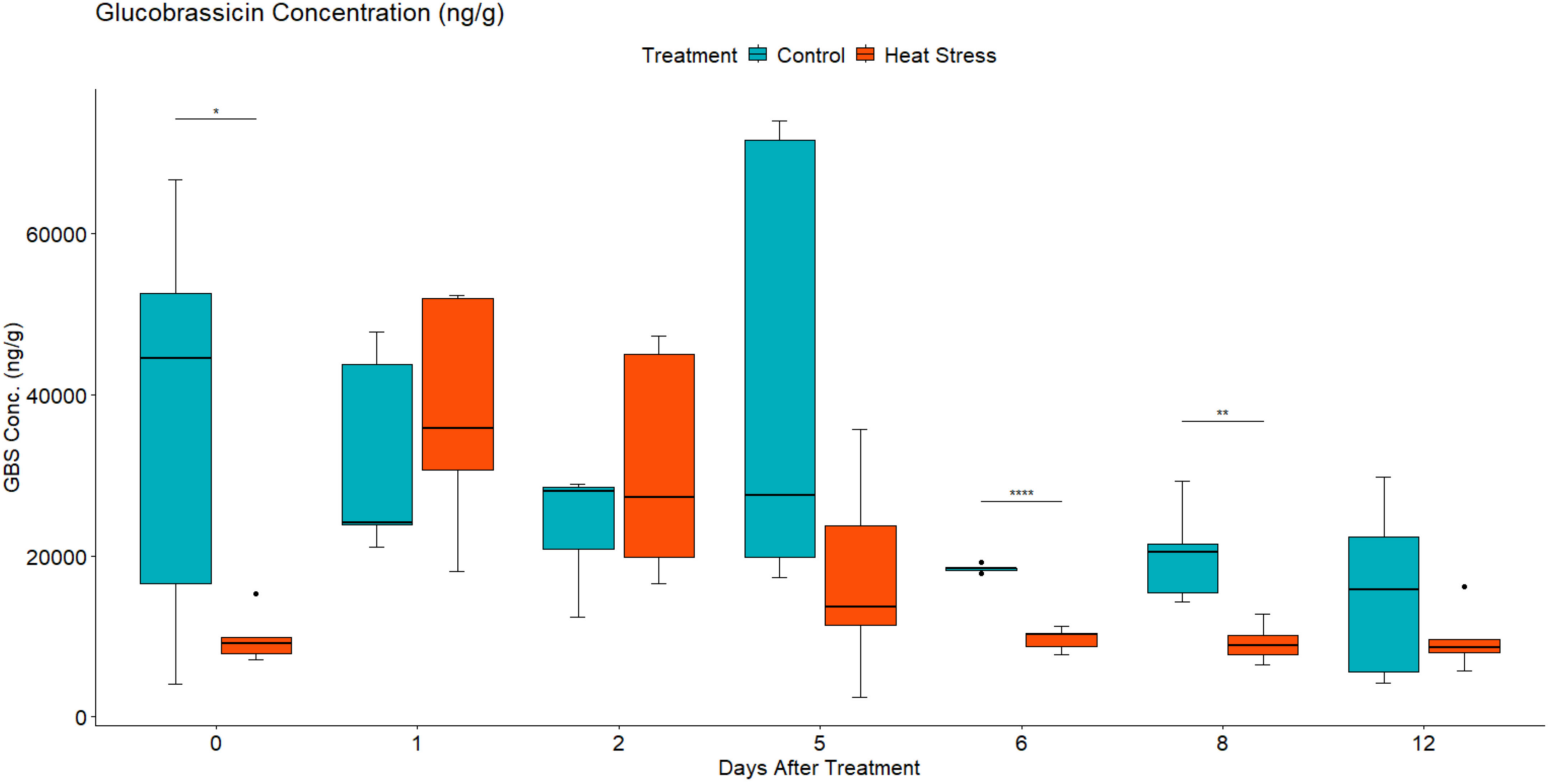
Leaf glucobrassicin (GBS) concentration in ng/g of dry weight (DW) in *Brassica napus* plants subjected to heat treatment (30°C/24°C day/night) at 0–5 DAT (red) or maintained under control temperatures (20°C/14°C day/night) at all timepoints (blue). GLS concentrations were quantified using LC-MS. Data were analysed using two-way mixed ANOVA pairwise comparisons. Each box plot represents data from five replicates, showing the median (horizontal bar), 25th and 75th percentiles (the box), and the min and max values (whiskers), with outliers as dots. Significant differences between heat-treated and control plants at each timepoint are denoted by (*) for p-value ≤ 0.05 or (**) for p-value ≤ 0.01 or (****) for p-value ≤ 0.0001. Heat treatment significantly decreased leaf concentration of the indolic GLS glucobrassicin.

Given the changes observed in the abundance of transcripts associated with GLS metabolism and sulphur assimilation and transport, the concentration of two aliphatic GLS progoitrin (PRO) and gluconapin (GNA), and one indole GLS glucobrassicin (GBS) were quantified using LC-MS. Their role in response to different abiotic stress factors has been previously reported (Ljubej et al., 2021; Jasper et al., 2020). Two-way mixed ANOVA pairwise comparisons showed a gradual increase in both PRO and GNA concentration in response to heat (**Figures 6**, **7**). This increase was significant at 2 DAT. After 5 days of the heat treatment, the concentration of GLS started to decline but remained higher in heat-treated plants than in the leaves of control plants. In contrast, the concentration GBS decreased in response to heat treatment (**Figure 8**). During recovery, the concentration of GBS continued to decrease with the difference being significant at 1 and 3 DOR. After 1 week of recovery, GBS concentration in heat-treated plants remained lower than that of the control.

#### 3.4.4 Heat treatment downregulated the transcript levels of most sugar transporter genes

**Figure 9:**
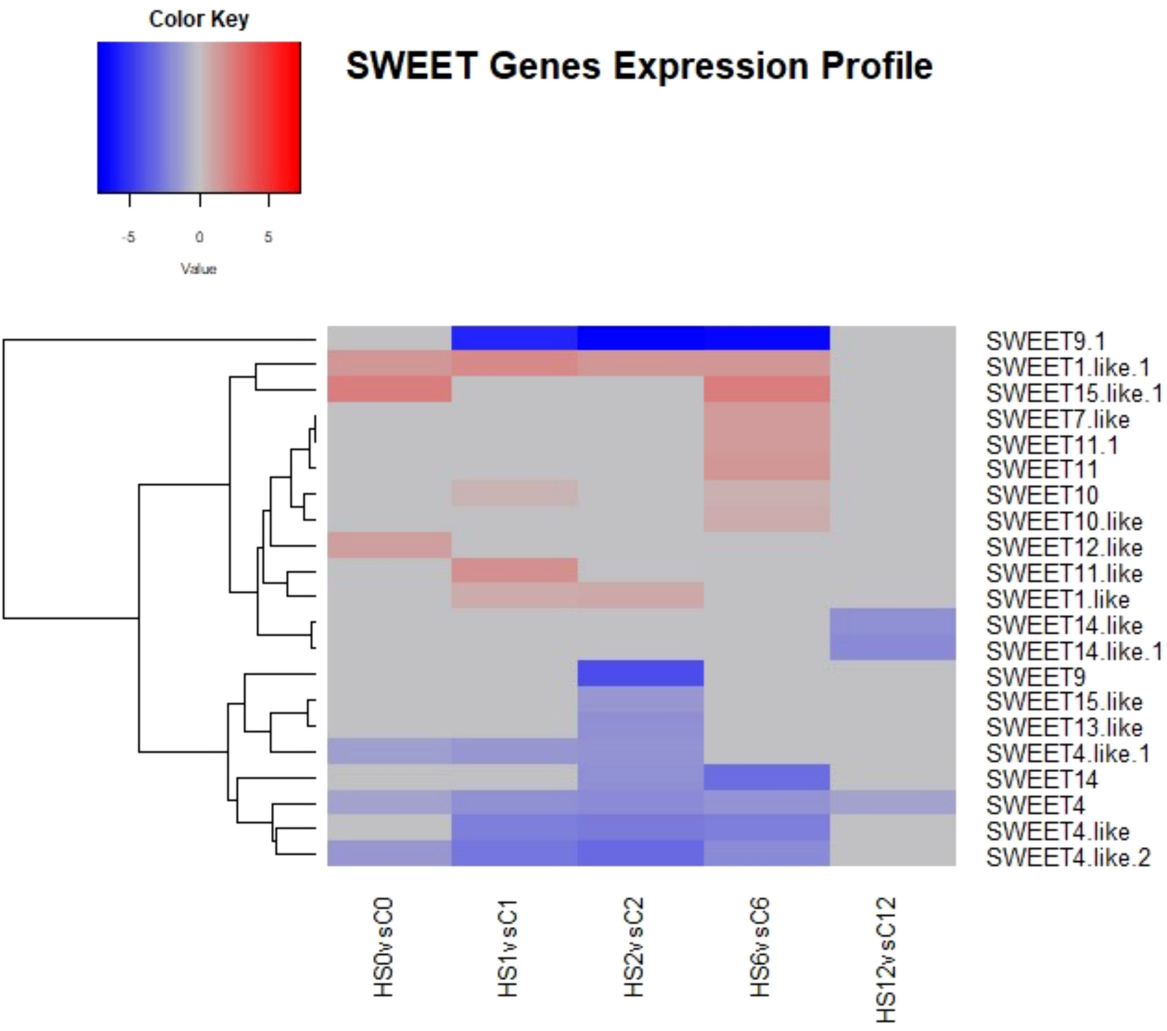
Heatmap showing differential expression of SWEET genes involved in sugar transport in *Brassica napus*. Heat stress significantly altered the expression of 21 SWEET genes with logFC ranged between −5 and 5.

To investigate the effect of heat stress on sugars transport and metabolism, analyses of genes encoding the SWEET and ERD6-like sugars transporters were undertaken. Compared to the control plants, heat stress significantly altered the expression of 21 SWEET genes with logFC ranged between −5 and 5 (**Figure 9**). These genes belonged to the phylogenetic clades II and III. Members of these clades predominantly transport hexose and sucrose, respectively. Nine of these transcripts were downregulated at 2 DAT, while two were upregulated. Interestingly, out of the 13 genes that were DE at 1 DOR, five upregulated genes were not DE during heat stress (were not affected by heat treatment) and became upregulated after the removal of stress (**Figure 9**).

**Figure 10:**
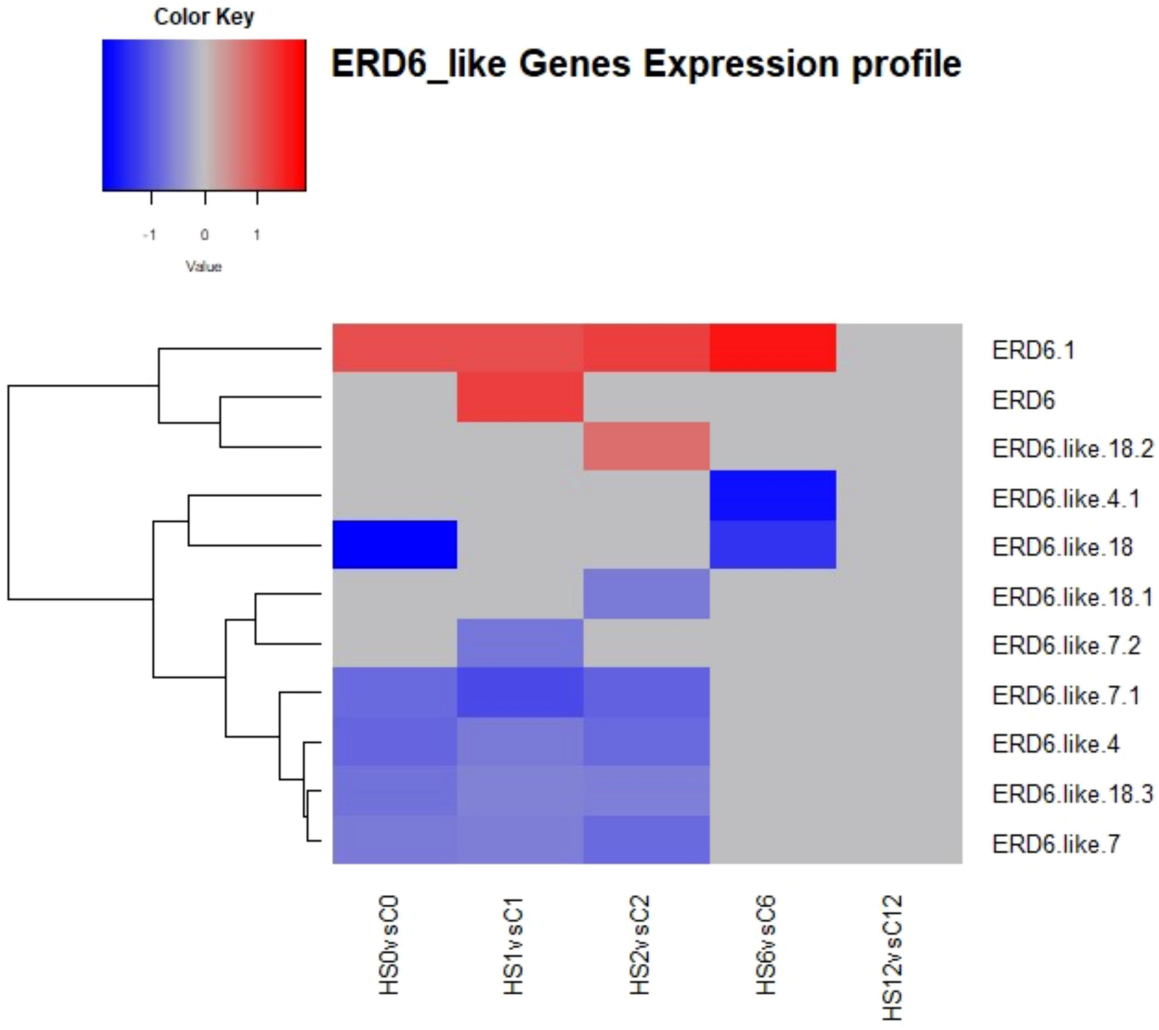
Heatmap showing differential expression of ERD-6 like genes involved in sugars transport in *Brassica napus*. At 2 DAT, five transcripts were downregulated, and two transcripts were upregulated.

Analysis of genes encoding the tonoplastic glucose symporters showed that 11 transcripts mapped to different ERD6-like genes (**Figure 10**). At 2 DAT, five were downregulated and two were upregulated.

In addition, one transcript mapping to a facilitated glucose transporter member 8-like showed a LogFC of −9.2 at 1 DAT and transcripts encoding sucrose transporters (SUCs) were found upregulated at 1 DAT and 1 DOR (**Supplementary Figure S28**). In addition to their significant role in phloem loading and unloading, these transporters are also involved in sugars influx into the cytosol. Furthermore, the present results showed that the expression of transcripts mapping to the sucrose-synthesizing enzyme, sucrose phosphate synthase (SPS) increased during heat treatment (**Supplementary Table S5**), while the expression of sucrose catalysing enzymes, namely, sucrose synthase and cell wall invertase significantly decreased (**Supplementary Table S6**).

#### 3.4.5 Metabolic analysis revealed an increase in leaf concentration of sugars in response to heat treatment

**Figure 11:**
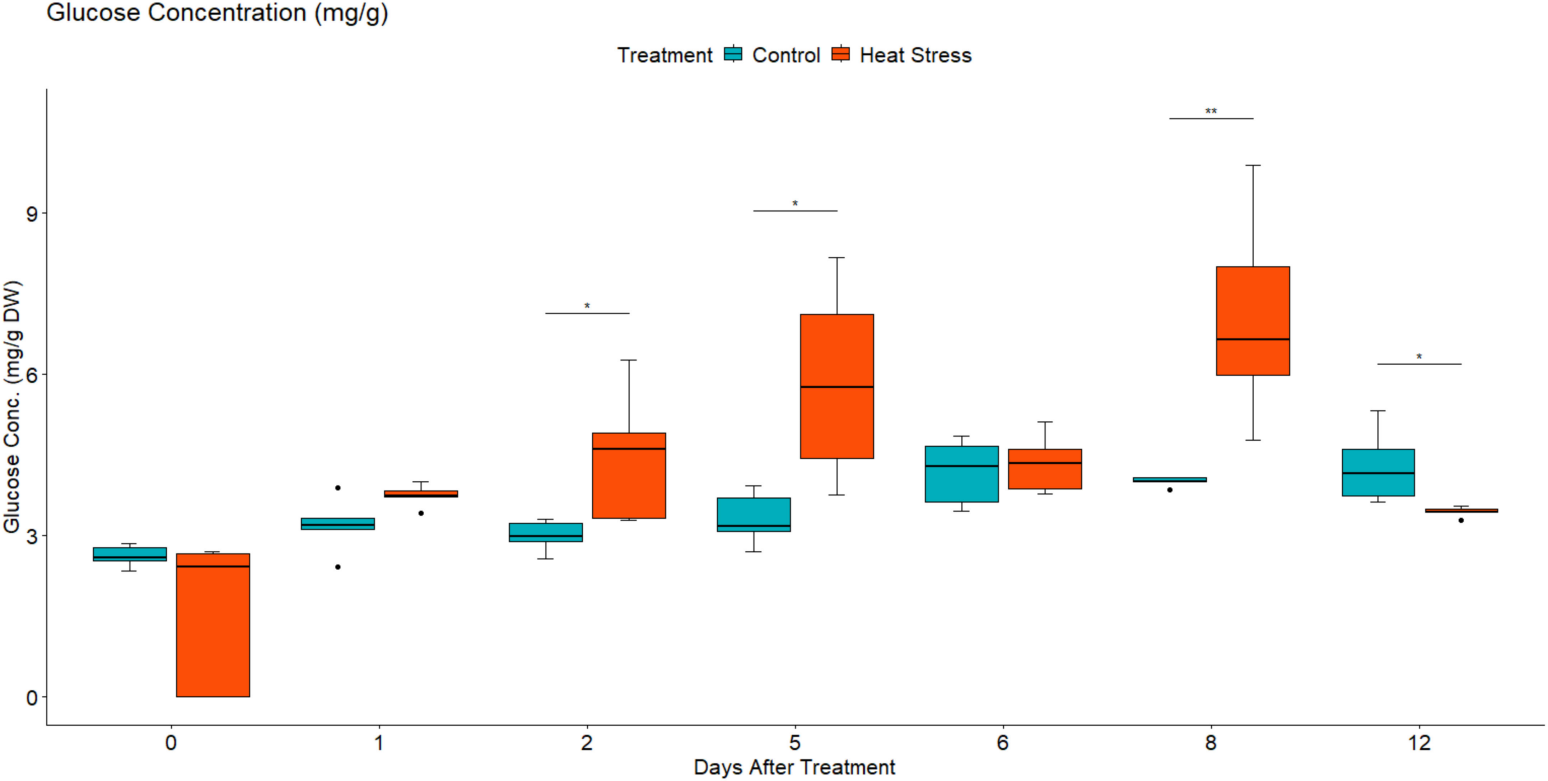
Leaf glucose concentration in mg/g of dry weight (DW) in *Brassica napus* plants subjected to heat treatment (30°C/24°C day/night) at 0–5 DAT (red) or maintained under control temperatures (20°C/14°C day/night) at all timepoints (blue). Concentration was quantified using HPLC. Data were analysed using two-way mixed ANOVA pairwise comparisons. Each box plot represents data from five replicates, showing the median (horizontal bar), 25th and 75th percentiles (the box), and the min and max values (whiskers), with outliers as dots. Significant differences between heat-treated and control plants at each timepoint are denoted by (*) for p-value ≤ 0.05 or (**) for p-value ≤ 0.01. Heat treatment significantly increased leaf concentration of glucose.

**Figure 12:**
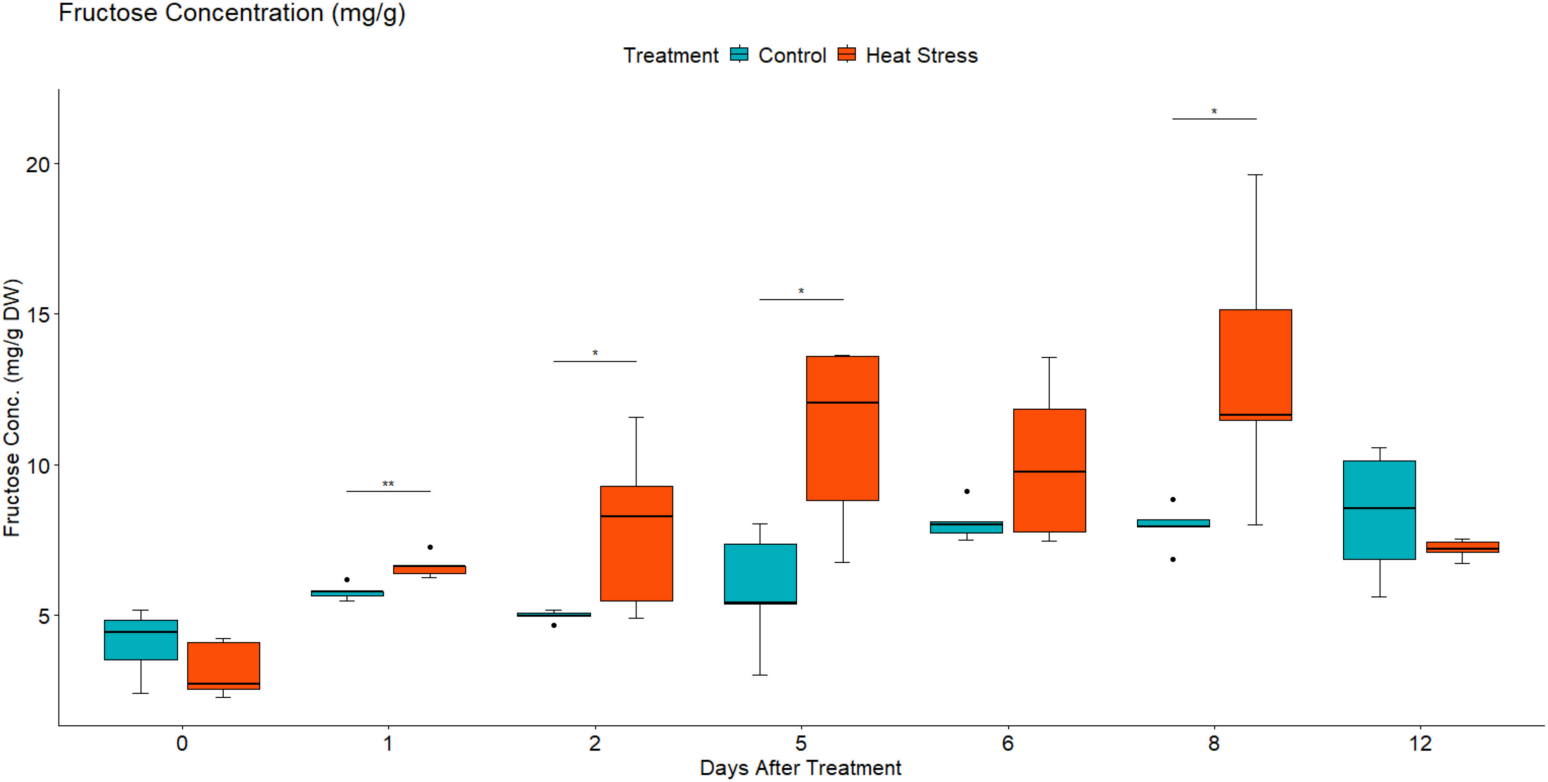
Leaf fructose concentration in mg/g of dry weight (DW) in *Brassica napus* plants subjected to heat treatment (30°C/24°C day/night) at 0–5 DAT (red) or maintained under control temperatures (20°C/14°C day/night) at all timepoints (blue). Concentration was quantified using HPLC. Data were analysed using two-way mixed ANOVA pairwise comparisons. Each box plot represents data from five replicates, showing the median (horizontal bar), 25th and 75th percentiles (the box), and the min and max values (whiskers), with outliers as dots. Significant differences between heat-treated and control plants at each timepoint are denoted by (*) for p-value ≤ 0.05 or (**) for p-value ≤ 0.01. Heat treatment significantly increased leaf concentration of fructose.

**Figure 13:**
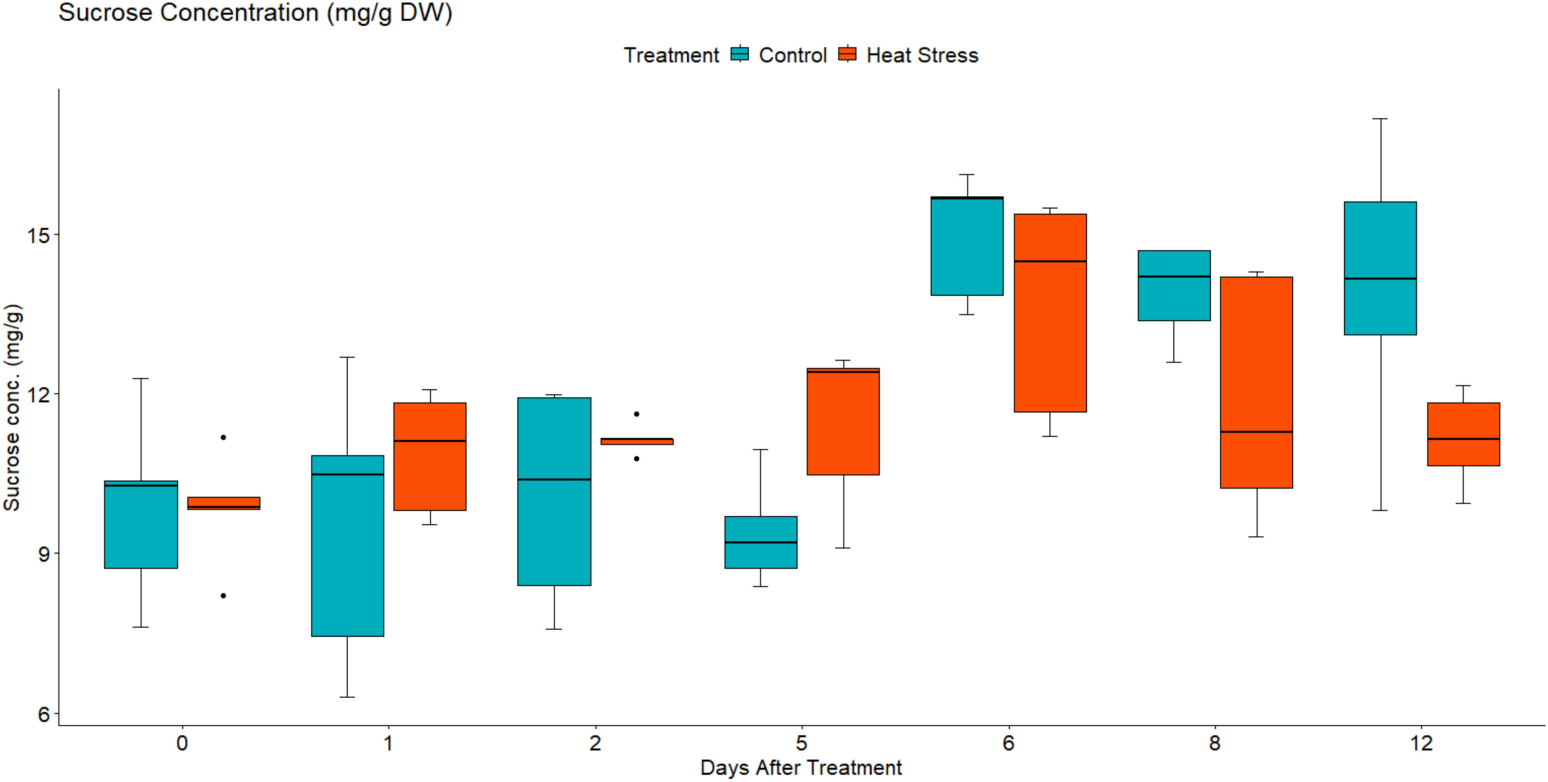
Leaf sucrose concentration in mg/g of dry weight (DW) in *Brassica napus* plants subjected to heat treatment (30°C/24°C day/night) at timepoints 0–5 (red) or maintained under control temperatures (20°C/14°C day/night) at all timepoints (blue). Concentration was quantified using HPLC. Data were analysed using two-way mixed ANOVA pairwise comparisons. Each box plot represents data from five replicates, showing the median (horizontal bar), 25th and 75th percentiles (the box) and the min and max values (whiskers), with outliers as dots.

To investigate the effect of heat stress on the sugar concentration in the leaves of *B. napus*, the concentration of fructose, glucose, and sucrose were quantified using high-performance liquid chromatography. Under control growth conditions, leaf fructose, glucose, and sucrose concentration increased slowly as the plants grew (**Figures 11**–**13**). In contrast, in the heat-treated plants, the leaf concentration of individual sugars increased. Two-way mixed ANOVA pairwise comparisons showed that the change in sugar concentration was significant during both heat treatment and recovery for glucose and fructose.

After the removal of heat stress, the leaf concentration of sucrose in the treated plants decreased gradually until it reached a lower concentration than that of the control plants at 3 DOR. In contrast, fructose and glucose concentration showed a slight decrease initially before they increased again significantly at 3 DOR. After 7 days of recovery, the content of fructose, glucose, and sucrose dropped substantially, reaching lower concentration than that of the control plants.

## 4 Discussion

To investigate the effect of prolonged high temperatures (heatwaves) on the growth and adaptability of *B. napus*, especially during its yield-determining reproductive stages, heat treatment experiment was designed in a way that mimic field temperature fluctuations. In the present study, *B. napus* plants were exposed to a gradual increase in temperature for a 6-day period, and samples were collected at different timepoints during treatment and recovery. The results showed a prevailing effect on the plants both physiologically and metabolically. Heat treatment negatively impacted seed weight and total plant biomass (**Supplementary Figure S4**), consistent with similar negative impacts documented in *B. napus* and in other plants such as *Arabidopsis* and wheat (Aksouh-Harradj et al., 2006; Zhang et al., 2017; Bheemanahalli et al., 2019). Bheemanahalli et al. (2019) reported a significant decrease in seed number (23%) and seed weight (34.6%) upon heat stress during flowering. Similarly, *Arabidopsis* plants exposed to heat stress at the bolting stage exhibited a reduction in silique length and increased sterility (Zhang et al., 2017). This suggests that heat stress during flowering irreversibly damages vital processes leading to reduced productivity. NIRS showed significant effects of heat treatment on fatty acid concentrations in seeds in response to heat (**Supplementary Figure S5**). Several studies have observed heat-induced alterations in lipid composition (Namazkar et al., 2016; Zhou et al., 2018; Zoong Lwe et al., 2021). For example, *B. napus* exposed to elevated temperature and CO_2_ levels showed a 77% reduction in linolenic acid content. Heat stress also significantly decreased other polyunsaturated fatty acids (Namazkar et al., 2016), highlighting the broader impact of heat on the plant’s lipid profile. Moreover, high night temperatures in *B. napus* have led to the overexpression of genes involved in fatty acid catabolism (Zhou et al., 2018). Different heat-tolerant and susceptible genotypes further displayed distinct heat-induced changes in lipid content (Narayanan et al., 2016, 2020), highlighting genotype-specific responses to heat stress. Because lipids and proteins are the major constituents of biological membranes, maintaining cellular homeostasis depends mainly on the dynamic nature of lipid composition (Zheng et al., 2011). This suggests an essential role of cellular membrane lipid remodelling under high temperature stress. Additionally, under periods of reduced carbon availability in plants, the conversion of lipids to organic acids provides an additional source of energy, thus contributing to the plants’ acclimation process (Yuenyong et al., 2019).

At the metabolomic level, heat treatment significantly altered the level of GLS and sugars and led to the differential expression of many genes involved in their synthesis, metabolism, and transport. Our study also demonstrated an increase of glucose, fructose, and sucrose concentration in leaf in during heat stress, with level slowly reaching, similar or slightly less levels to their control counterpart upon removal and recovery from the stressor. GLS and sugars are among the major secondary metabolites naturally occurring in *Brassica* species (Ayaz et al., 2006; Rao et al., 2021). Their primary site of synthesis occurs mainly in the leaves, from where they are transported to other parts of the plant (Pacheco-Sangerman et al., 2023; Ren et al., 2022). While reports on the sugar content of leaves are common (Ren et al., 2022; Zafar et al., 2022; Dellero et al., 2024), GLS have been thoroughly investigated in seeds (Wang et al., 2019; Tang et al., 2023; Jhingan et al., 2023), with fewer studies focusing on their presence in leaves (Rhee et al., 2020).

In this study, the levels of GLS and sugars were extracted and quantified from *B. napus* leaves, and their roles in heat stress response were investigated.

### 4.1 Heat stress resulted in differential expression of many genes involved in multiple functional and metabolic processes especially at 2 DAT

In this study, comparative transcriptome analysis between heat treatment and control identified 9,933 significantly differentially expressed transcripts (|log2(foldchange)|≥0.5, FDR ≤ 0.05) (**Supplementary Figure S8A**) with a balanced proportion of up- and downregulated genes in contrast HS0vsC0 (0 DAT), and more downregulated than upregulated genes in the rest of the contrasts were identified. This suggests that the impact of heat treatment on the plant’s transcriptome after 24 h of heat stress, resulted in downregulation of many genes involved in multiple functional and metabolic processes such as glycolysis, PPP, citrate cycle, and oxidative phosphorylation. In contrast, HS12vsC12 (7 DOR), although the proportion of downregulated genes was higher than the upregulated ones, the total number of DEG decreased dramatically as the plant started to restore its metabolic functions after 7 days of recovery.

Differential expression analysis showed that some of the genes encoding sugar transporters, nitrogen transport and storage, cell wall modification, and methylation were downregulated (**Supplementary Tables S8**, **S10**). In contrast, several genes mapping to small heat shock proteins (sHSP), other heat shock transcription factors, methylation, and redox balance-related genes were upregulated (**Supplementary Tables S7**, **S9**). Among the top DE transcripts were several transcripts that mapped to HSPs and other HSFs. Notably, members of the sHSP family, also known as the HSP20 family proteins, such as HSP17.6, HSP22, and HSP23.6, showed high expression levels throughout the heat treatment, with expression reaching a log2FC of 11 at 2 DAT. This protein family is the most abundantly produced in plants in response to heat stress, and their role in plant responses to various abiotic stresses has been established (Sun et al., 2020). Other studies also reported the involvement of HSFs in different biotic and abiotic stress (Mishra et al., 2002, 2019; Chowdhary et al., 2023). In *A. thaliana*, thermoprotection tests demonstrated the role of HSFA6b in thermotolerance acquisition (Huang et al., 2016). Moreover, Giorno et al. (2009) showed that HsfA2 and Hsp17-CII are induced under high temperature in tomato, which are responsible for activating protection mechanisms during heat stress.

### 4.2 DEGs showed enrichment of pathways related to respiratory metabolism

The analysis of KEGG-enriched pathways revealed the main underlying biochemical pathways altered in *B. napus* in response to heat stress (**Supplementary Figures S16**-**S19**). In accordance with other reports (Estravis-Barcala et al., 2021; Li et al., 2022), DEGs showed an enrichment of pathways related to respiratory metabolism, namely, glycolysis, citrate cycle, oxidative phosphorylation, and pentose phosphate pathway (PPP). At 2 DAT, the impact of heat stress on glycolysis was manifested by the downregulation of the key enzymes phosphoglucomutase, aldose 1-epimerase, hexokinase, glucose-6-phosphate isomerase, and 6-phosphofructokinase (**Supplementary Figure S21**). Moreover, most of the citrate cycle genes were also downregulated at 2 DAT (**Supplementary Figure S22**). In the electron transport chain (ETC), gene expression analysis showed that most of the involved genes were downregulated under heat treatment, except for succinate dehydrogenase (SDH), which was upregulated (**Supplementary Figure S23**). In mitochondrial metabolism, SDH also known as complex II, plays a central role in the citrate cycle and ETC (Huang and Millar, 2013). Additionally, SDH is thought to impact different components of the plant stress response, including stomatal conductance and ROS scavenging (Huang et al., 2019).

Although respiratory pathways have been found to have potential roles in adaptive response to abiotic stress, mainly through reducing the production of ROS (Van Dongen et al., 2011), under extreme conditions such as severe or prolonged heat stress, respiratory enzymes are deactivated, and proteins are denatured leading to culminating of ROS and a total breakdown of mitochondrial respiration (Scafaro et al., 2021). This negatively impacts oxygen and carbon fluxes and eventually leads to more severe yield penalty (Rasmusson et al., 2020). In line with this, results from heat stress experiment on seagrass showed that high midday temperature stress of 40°C was associated with significant decrease in biomass (George et al., 2018). Likewise, Impa et al. (2019) found that under high night temperature, wheat tolerant cultivar exhibited smaller reductions in biomass and lower rates of both net photosynthesis and respiration compared to other wheat cultivars (Impa et al., 2019).

In the PPP, the oxidative phase (OPPP) was mainly affected at 1 DAT, and this was indicated by the differential expression of glucose-6-phosphate dehydrogenase (G6PDH) and 6-phosphogluconate dehydrogenase (6PGDH) enzymes (**Supplementary Figure S24**). G6PDH and 6PGDH are the key enzymes in the OPPP. They catalyse the first and third steps of the pathway, respectively (Long et al., 2016), and have been reported to be associated with the response to various abiotic stresses in plants (Gong et al., 2012). Esposito (2016) reported the involvement of the OPPP in the early response to abiotic stress factors, forming a true metabolic sensor to oxidative stress. At 2 DAT, most of the oxidative and non-oxidative phase enzymes were downregulated, including transketolase (TK), transaldolase, and phosphofructokinase enzymes (**Supplementary Figure S25**). TK has a central role in primary metabolism, where the products of the involved reactions produce the precursors for nucleic acids biosynthesis, aromatic amino acids, and vitamins (Bi et al., 2013). Bi et al. (2013) showed that the cucumber TK gene (*CsTK*) was sensitive to temperature and light (Bi et al., 2013, 2019). Likewise, the transaldolase gene was found to be involved in the regulation of expression of genes involved in the ABA signalling pathway and the enzymes responsive to ROS (Rong et al., 2021). The results of this study add further evidence to the involvement of the PPP enzymes in the heat stress response in *B. napus*.

### 4.3 Heat stress altered GLS metabolism during treatment

At the transcriptomic level, heat treatment differentially affected the expression of GLS synthesis and regulatory genes (**Figures 3**, **4**). Several genes encoding different UDP glycosyltransferases and glutathione S-transferases appeared downregulated, while others encoding GLS-related transcription factors were upregulated, suggesting that these genes might have participated at different stages of the stress response.

The influence of heat stress on individual GLS was further evaluated through metabolomic analysis of two aliphatic GLS [progoitrin (PRO) and gluconapin (GNA)] and one indolic GLS [glucobrassicin (GBS)]. The role of these GLS in response to different abiotic stress factors has been previously reported (Jasper et al., 2020; Ljubej et al., 2021). In the current study, the concentration of PRO and GNA significantly increased in response to heat stress before it started to decline at 5 DAT (**Figures 6**, **7**). This decline could have resulted from membrane damage caused by prolonged exposure to heat, leading to GLS degradation. In addition, the removal of heat stress after 5 DAT could also be another factor for the decline in GLS content. In addition to its primary role in response to plant pathogen interaction (Hopkins et al., 2009; Touw et al., 2020), GLS accumulation in response to heat stress has also been previously reported (Valente Pereira et al., 2002; Martínez-Ballesta et al., 2013; Jasper et al., 2020). When plants are stressed, growth is reduced, and carbon utilisation is predominantly diverted towards the production of secondary metabolites. As part of the plant defence mechanism, GLS play important role as osmoprotective compounds (Martínez-Ballesta et al., 2013), where their concentration increases in response to stress, providing protection against oxidative damage. At 30°C/15°C (day/night) temperature regime, *Brassica oleracea* seedlings had significantly higher GLS concentration than plants cultivated at lower temperatures (22°C/15°C and 18°C/12°C) (Valente Pereira et al., 2002). Similarly, an *Arabidopsis* GLS mutant experienced a remarkable decline in growth and development and was found to be less heat tolerant than wild-type plants upon exposure to high temperature (Ludwig-Müller et al., 2000).

In contrast, indolic glucosinolate GBS showed a downward trend where concentration decreased gradually in response to heat (**Figure 8**). Similar results have been found where GBS concentration was reduced in response to heat treatment (Bohinc and Trdan, 2012). In addition, during short-term high-temperature stress, the transcript level of the indolic glucosinolates synthetic genes was predominantly downregulated (Rao et al., 2021). Several studies reported that indole GLS are much more sensitive to heat treatment (Bones and Rossiter, 2006; Bohinc and Trdan, 2012) and can demonstrate more thermal degradation than aliphatic GLS at lower temperatures (Orlemans et al., 2006).

The results of our study indicate that the concentration of GLS in the leaves of *B. napus* is influenced by high-temperature stress, which was previously reported in other *Brassica* species (Rask et al., 2000; Bohinc and Trdan, 2012). It also shows that different groups of GLS (aliphatic versus indolic) differ in their response to heat stress. As reported by Ljubej et al. (2021), the different accumulation trends of these groups could correspond to different protective mechanisms driven by individual GLS with diverse chemical structures and bioactivities (Ljubej et al., 2021). These mechanisms may prioritise energy towards aliphatic GLS production, which are known to play crucial roles in ROS mitigation (Martínez-Ballesta et al., 2013). Additionally, the reduced availability of GLS precursors, such as nitrogen and sulphur, under stressful conditions may influence the allocation of GLS across plant tissues. In response, plants might break down their GLS and utilise the released sulphur to support primary metabolic processes, such as protein synthesis, within specific organs (Brown et al., 2003; Martínez-Ballesta et al., 2013). This plasticity highlights GLS players in balancing stress resilience and adaptation. By selectively modifying GLS content, plants can fine-tune their responses to stress, meeting immediate defence demands while ensuring long-term survival and reproductive success.

### 4.4 Heat stress altered the expression of sulphur assimilation and transport genes

In the current study, the transcript levels of sulphur assimilation and transport genes were significantly downregulated especially at 2 DAT (**Figure 5**). This included several adenylyl sulphate kinases and reductases, ATP sulphurylase (ATPS), and sulphate transporter genes. These genes are important for the activation, catalysis, and transport of sulphur compounds (Capaldi et al., 2015). Moreover, enrichment pathway analysis showed that cysteine and methionine metabolism pathway was among the top significantly enriched pathways at 1 and 2 DAT (**Supplementary Figures S16**, **S17**). In this pathway, 11 transcripts mapping to S-adenosylmethionine synthetase and 11 transcripts mapping to homocysteine S-methyltransferases were downregulated (**Supplementary Figure S20**). These enzymes are among the key enzymes that control the methionine pool (Capaldi et al., 2015). Given that GLS compounds are rich in sulphur, assimilation of sulphur is closely associated with GLS biosynthesis. Cysteine, which is the terminal metabolite in sulphur assimilation, acts as a sulphur donor for methionine, a precursor for aliphatic GLS. This renders cysteine an important intersection point between sulphur assimilation and GLS biosynthesis (Rao et al., 2021). Such proximity between sulphur and these amino acids makes cysteine and methionine metabolism pathway directly related to GLS biosynthesis.

### 4.5 Heat stress altered sugar metabolism during treatment and recovery periods

In the present study, heat treatment altered carbohydrate concentration as the amounts of glucose, fructose, and sucrose increased with heat treatment (**Figures 11**-**13**). At the transcriptional level, the starch hydrolysing enzyme alpha-amylase was induced. Conversely, the starch-synthesizing enzymes such as phosphoglucomutase (PGM), AGPase, and hexokinase were inhibited (**Supplementary Figure S21**, **S27**). Likewise, heat induced the upregulation of sucrose-synthesizing enzymes and the downregulation of sucrose-hydrolysing enzymes such as cell wall invertase and sucrose synthase (**Supplementary Tables S5**, **S6**). These results suggest that heat-induced starch hydrolysis and promoted sucrose synthesis. Furthermore, heat stress downregulated many sugars transporters such as SWEET and ERD6-like (**Figures 9**, **10**) and led to sugar accumulation in the leaves. Several studies have shown that various environmental stresses significantly impact sugar metabolism and transport in plants (Xalxo et al., 2020; Mathan et al., 2020). For instance, drought and salinity stress have been reported to increase sucrose content in both leaf and root tissues of rice (Mathan et al., 2020). Similarly, heat-stressed moth bean seedlings exhibited a notable accumulation of total sugars and proline across different genotypes (Harsh et al., 2016).

Heat stress not only impairs carbohydrate assimilation but also reduces sugar export from leaves (Julius et al., 2017). Marias et al. (2017) observed increased glucose and fructose levels in the leaves of *Coffea arabica* under heat stress. In maize, high temperatures decreased the 13C export rate from ear leaves, leading to enhanced growth of vegetative parts but reduced grain yield (Suwa et al., 2010). It is proposed that SWEET transporters efflux sucrose into the phloem apoplast, while SUT transporters import sucrose into the sieve element-companion cell complex (Osorio et al., 2014). During heat stress, leaves are the first tissues to sense and suffer from heat damage, and they utilise sugars to scavenge reactive oxygen species (ROS) and alleviate oxidative stress. Additionally, sugars serve as an energy source in high-respiration environments, helping maintain cell–water balance and membrane integrity through osmotic adjustments (Burke, 2007). Given the critical role of sugars in stress responses, the findings of this study, combined with previous observations, suggest that heat stress inhibits sugars export by SWEETs and/or its import by SUTs in heat-stressed leaves, resulting in sugars accumulation. Thereby, maintaining leaf sugar content through reducing source to sink export is considered a tolerance strategy (Kaushal et al., 2013). It is also worth noting that the decrease in sink demand due to growth limitation could also contribute to the accumulation of sugars in the source leaves under stressful conditions (Hummel et al., 2010; Lemoine et al., 2013).

## 5 Conclusion

Through a comprehensive transcriptomic and metabolomic analysis, this study brings evidence for the effect of high temperature stress and, in particular, heatwaves on different functional and metabolic pathways, highlighting its role in the plant stress defence system. More specifically, our results indicated that heat treatment:

1. inhibited important metabolic pathways such as respiratory metabolism, namely, glycolysis, pentose phosphate pathway, citrate cycle, and oxidative phosphorylation especially after 48 h of heat treatment;
2. reduced yield and plant biomass and altered seed composition;
3. altered sugar and glucosinolate levels in leaves;
4. induced the expression of 9,933 genes, which were differentially regulated during heat treatment and recovery. Most of the top up- and downregulated genes involved in key biological processes such as heat shock proteins, cellular processes regulation, transcription factors, cell wall remodelling, sugar and secondary metabolites transport, and metabolism.

Altogether, this study is an attempt to tackle the question of how plants face and adapt to heat stress and to provide insights into the impact of a future warmer climate on plants especially during reproductive stages. At the transcriptomic level, the key DEGs identified in this study could serve as important biomarkers that can be utilised by breeding programs to select cultivars with stronger resistance to heat. In particular, the identified genes could be further characterised and used as genetic markers for marker assisted selection, where plant breeders could select plant performance based on the composition of these genetic biomarkers rather than through waiting for the plants’ phenotypic performance, thus accelerating traditional crop breeding for stress tolerance traits. In addition, the identified genes could form a target for genome editing techniques such as the clustered regularly interspaced short palindromic repeat (CRISPR)/CRISPR-associated protein 9 (Cas9), an approach that has already been explored in crop improvement programs especially for rapid development of abiotic stress-tolerant crops (Zafar et al., 2020). This can be applied by modifying sensitive or negatively regulating genes and activating positively regulated genes involved in stress response pathways to enhance the plant’s ability to withstand stress (Kumar et al., 2023). Finally, this study represents an important step towards developing an understanding of the heat stress response and tolerance mechanisms, a knowledge that could be transferred to other plants.

## Data Availability

The datasets presented in this study can be found in online repositories. The names of the repository/repositories and accession number(s) can be found below: https://www.ncbi.nlm.nih.gov/, SUB14296957.

## References

[B1] Aksouh-Harradj N. Campbell L. Mailer R. (2006). Canola response to high and moderately high temperature stresses during seed maturation. Can. J. Plant Sci. 86, 967–980. doi: 10.4141/P05-130

[B2] Andrews S. (2012). FastQC: a quality control tool for high throughput sequence data. Available online at: http://www.bioinformatics.babraham.ac.uk/projects/fastqc (Accessed October 01, 2024).

[B3] Angadi S. V. Cutforth H. W. Miller P. R. McConkey B. G. Entz M. H. Brandt S. A.. (2000). Response of three Brassica species to high temperature stress during reproductive growth. Can. J. Plant Sci. 80, 693–701. doi: 10.4141/P99-152

[B4] (2019). OmicsBox – Bioinformatics Made Easy, BioBam Bioinformatics. Available online at: https://www.biobam.com/omicsbox (Accessed October 10, 2024).

[B5] Ayaz F. A. Glew R. H. Millson M. Huang H. S. Chuang L. T. Sanz C.. (2006). Nutrient contents of kale (*Brassica oleraceae* L. var. acephala DC.). Food Chem. 96, 572–579. doi: 10.1016/j.foodchem.2005.03.011

[B6] Bell L. Oruna-Concha M. J. Wagstaff C. (2015). Identification and quantification of glucosinolate and flavonol compounds in rocket salad (*Eruca sativa*, *Eruca vesicaria* and *Diplotaxis tenuifolia)* by LC–MS: Highlighting the potential for improving nutritional value of rocket crops. Food Chem. 172, 852–861. doi: 10.1016/j.foodchem.2014.09.116 25442630 PMC4245720

[B7] Bheemanahalli R. Sunoj V. S. J. Saripalli G. Prasad P. V. V. Balyan H. S. Gupta P. K.. (2019). Quantifying the impact of heat stress on pollen germination, seed set, and grain filling in spring wheat. Crop Sci. 59, 684–696. doi: 10.2135/cropsci2018.05.0292

[B8] Bi H. Li F. Wang H. Ai X. (2019). Overexpression of transketolase gene promotes chilling tolerance by increasing the activities of photosynthetic enzymes, alleviating oxidative damage and stabilizing cell structure in *Cucumis sativus* L. Physiologia Plantarum 167, 502–515. doi: 10.1111/ppl.12903 30548278

[B9] Bi H. Wang M. Dong X. Ai X. (2013). Cloning and expression analysis of transketolase gene in Cucumis sativus L. Plant Physiol. Biochem. 70, 512–521. doi: 10.1016/j.plaphy.2013.06.017 23860231

[B10] Bohinc T. Trdan S. (2012). Environmental factors affecting the glucosinolate content in Brassicaceae. J. Food Agric. Environ. 10, 357–360.

[B11] Bones A. M. Rossiter J. T. (2006). The enzymic and chemically induced decomposition of glucosinolates. Phytochemistry 67, 1053–1067. doi: 10.1016/j.phytochem.2006.02.024 16624350

[B12] Brown P. D. Tokuhisa J. G. Reichelt M. Gershenzon J. (2003). Variation of glucosinolate accumulation among different organs and developmental stages of *Arabidopsis thaliana* . Phytochemistry 62, 471–481. doi: 10.1016/S0031-9422(02)00549-6 12620360

[B13] Burke J. J. (2007). Evaluation of source leaf responses to water-deficit stresses in cotton using a novel stress bioassay. Plant Physiol. 143, 108121. doi: 10.1104/pp.106.087783 PMC1761985 17071650

[B14] Capaldi F. R. Gratão P. L. Reis A. R. Lima L. W. Azevedo R. A.. (2015). Sulfur Metabolism and Stress Defense Responses in Plants. Tropical Plant Biology. 8 (3–4), 60–73. doi: 10.1007/s12042-015-9152-1

[B15] Chowdhary A. A. Mishra S. Mehrotra S. Upadhyay S. K. Bagal D. Srivastava V. (2023). Plant transcription factors: An overview of their role in plant life. In Upadhyay S. K. (Ed.) Plant Transcription Factors, 3–20. doi: 10.1016/b978-0-323-90613-5.00003-0

[B16] Dellero Y. Berardocco S. Bouchereau A. (2024). U-13C-glucose incorporation into source leaves of *Brassica napus* highlights light-dependent regulations of metabolic fluxes within central carbon metabolism. J. Plant Physiol. 292, 154162. doi: 10.1016/j.jplph.2023.154162 38103478

[B17] Dikšaitytė A. Viršilė A. Žaltauskaitė J. Januškaitienė I. Juozapaitienė G. (2019). Growth and photosynthetic responses in *Brassica napus* differ during stress and recovery periods when exposed to combined heat, drought and elevated CO_2_ . Plant Physiol. Biochem. 142, 59–72. doi: 10.1016/j.plaphy.2019.06.026 31272036

[B18] Esposito S. (2016). Nitrogen assimilation, abiotic stress and glucose 6-phosphate dehydrogenase: The full circle of reductants. Plants (Basel Switzerland) 5, 24. doi: 10.3390/plants5020024 27187489 PMC4931404

[B19] Estravis-Barcala M. Heer K. Marchelli P. Ziegenhagen B. Arana M. V. Bellora N. (2021). Deciphering the transcriptomic regulation of heat stress responses in *Nothofagus pumilio* . PloS One 16, e0246615. doi: 10.1371/journal.pone.0246615 33784314 PMC8009359

[B20] George R. Gullström M. Mangora M. M. Mtolera M. S. P. Björk M. (2018). High midday temperature stress has stronger effects on biomass than on photosynthesis: A mesocosm experiment on four tropical seagrass species. Ecol. Evol. 8, 4508–4517. doi: 10.1002/ece3.3952 29760891 PMC5938440

[B21] Giorno F. Wolters-Arts M. Grillo S. Scharf K.-D. Vriezen W. H. Mariani C. (2009). Developmental and heat stress-regulated expression of HsfA2 and small heat shock proteins in tomato anthers. J. Exp. Bot. 61, 453–462. doi: 10.1093/jxb/erp316 19854799 PMC2803211

[B22] Glaubitz U. Li X. Schaedel S. Erban A. Sulpice R. Kopka J.. (2017). Integrated analysis of rice transcriptomic and metabolomic responses to elevated night temperatures identifies sensitivity- and tolerance-related profiles. Plant Cell Environ. 40, 121–137. doi: 10.1111/pce.12850 27761892

[B23] Goel K. Kundu P. Sharma P. Zinta G. (2023). Thermosensitivity of pollen: a molecular perspective. Plant Cell Rep. 42, 843–857. doi: 10.1007/s00299-023-03003-y 37029819

[B24] Gong H. Chen G. Li F. Wang X. Hu Y. Bi Y. (2012). Involvement of G6PDH in heat stress tolerance in the calli from *Przewalskia tangutica* and *Nicotiana tabacum* . Biol. plantarum 56, 422–430. doi: 10.1007/s10535-012-0072-8

[B25] Harsh A. Sharma Y. K. Joshi U. Rampuria S. Singh G. Kumar S.. (2016). Effect of short-term heat stress on total sugars, proline and some antioxidant enzymes in moth bean (*Vigna aconitifolia*). Ann. Agric. Sci. 61, 57–64. doi: 10.1016/j.aoas.2016.02.001

[B26] Hasanuzzaman M. Nahar K. Alam M. M. Fujita M. (2014). Modulation of antioxidant machinery and the methylglyoxal detoxification system in selenium-supplemented *Brassica napus* seedlings confers tolerance to high temperature stress. Biol. Trace Element Res. 161, 297–307. doi: 10.1007/s12011-014-0120-7 25249068

[B27] Hedhly A. (2011). Sensitivity of flowering plant gametophytes to temperature fluctuations. Environ. Exp. Bot. 74, 9–16. doi: 10.1016/j.envexpbot.2011.03.016

[B28] Hinojosa L. Matanguihan J. B. Murphy K. M. (2019). Effect of high temperature on pollen morphology, plant growth and seed yield in quinoa (*Chenopodium quinoa* Willd.). J. Agron. Crop Sci. 205, 33–45. doi: 10.1111/jac.2019.205.issue-1

[B29] Hopkins R. J. Van Dam N. M. Van Loon J. J. (2009). Role of glucosinolates in insect-plant relationships and multitrophic interactions. Annu. Rev. Entomol. 54, 57–83. doi: 10.1146/annurev.ento.54.110807.090623 18811249

[B30] Huang R. Liu Z. Xing M. Yang Y. Wu X. Liu H.. (2019). Heat stress suppresses *Brassica napus* seed oil accumulation by inhibition of photosynthesis and BnWRI1 pathway. Plant Cell Physiol. 60, 1457–1470. doi: 10.1093/pcp/pcz052 30994920

[B31] Huang S. Millar A. H. (2013). Succinate dehydrogenase: the complex roles of a simple enzyme. Curr. Opin. Plant Biol. 16, 344–349. doi: 10.1016/j.pbi.2013.02.007 23453781

[B32] Huang Y. C. Niu C. Y. Yang C. R. Jinn T. L. (2016). The heat stress factor HSFA6b connects ABA signalling and ABA-mediated heat responses. Plant Physiol. 172, 1182–1199. doi: 10.1104/pp.16.00860 27493213 PMC5047099

[B33] Hummel I. Pantin F. Sulpice R. Piques M. Rolland G. Dauzat M.. (2010). Arabidopsis plants acclimate to water deficit at low cost through changes of carbon usage: an integrated perspective using growth, metabolite, enzyme, and gene expression analysis. Plant Physiol. 154, 357–372. doi: 10.1104/pp.110.157008 20631317 PMC2938159

[B34] Ikram M. Chen J. Xia Y. Li R. Siddique K. H. M. Guo P. (2022). Comprehensive transcriptome analysis reveals heat-responsive genes in flowering Chinese cabbage (*Brassica campestris* L. ssp. chinensis) using RNA sequencing. Front. Plant Sci. 13. doi: 10.3389/fpls.2022.1077920 PMC9755508 36531374

[B35] Impa S. M. Sunoj V. S. J. Krassovskaya I. Bheemanahalli R. Obata T. Jagadish S. V. K. (2019). Carbon balance and source-sink metabolic changes in winter wheat exposed to high night-time temperature. Plant Cell Environ. 42, 1233–1246. doi: 10.1111/pce.13488 30471235

[B36] Ismaili A. Salavati A. Mohammadi P. P. (2015). A comparative proteomic analysis of responses to high-temperature stress in hypocotyl of canola (*Brassica napus* L.). Protein Pept. Lett. 22, 285–299. doi: 10.2174/0929866521666141124102755 25420948

[B37] Jagadish S. K. Way D. A. Sharkey T. D. (2021). Plant heat stress: concepts directing future research. Plant Cell Environ. 44, 1992–2005. doi: 10.1111/pce.14050 33745205

[B38] Jasper J. Wagstaff C. Bell L. (2020). Growth temperature influences postharvest glucosinolate concentrations and hydrolysis product formation in first and second cuts of rocket salad. Postharvest Biol. Technol. 163, 111157. doi: 10.1016/j.postharvbio.2020.111157 32362723 PMC7104888

[B39] Jhingan S. Harloff H. J. Abbadi A. Welsch C. Blümel M. Tasdemir D.. (2023). Reduced glucosinolate content in oilseed rape (*Brassica napus* L.) by random mutagenesis of *BnMYB28* and *BnCYP79F1* genes. Sci. Rep. 13, 2344. doi: 10.1038/s41598-023-28661-6 36759657 PMC9911628

[B40] Jin B. Li W. Jing W. Jiang K. Z. Yang W. Jiang X. X.. (2011). The effect of experimental warming on leaf functional traits, leaf structure and leaf biochemistry in *Arabidopsis thaliana* . BMC Plant Biol. 11, 35. doi: 10.1186/1471-2229-11-35 21329528 PMC3045891

[B41] Julius B. T. Leach K. A. Tran T. M. Mertz R. A. Braun D. M. (2017). Sugar transporters in plants: new insights and discoveries. Plant Cell Physiol. 58, 1442–1460. doi: 10.1093/pcp/pcx090 28922744

[B42] Kanehisa M. Goto S. (2000). KEGG: Kyoto encyclopedia of genes and genomes. Nucleic Acids Res. 28, 27–30. doi: 10.1093/nar/28.1.27 10592173 PMC102409

[B43] Kaushal N. Awasthi R. Gupta K. Gaur P. Siddique K. H. M. Nayyar H. (2013). Heat-stress-induced reproductive failures in chickpea (*Cicer arietinum*) are associated with impaired sucrose metabolism in leaves and anthers. Funct. Plant Biol. 40, 1334–1349. doi: 10.1071/FP13082 32481199

[B44] Koscielny C. B. Hazebroek J. Duncan R. W. (2018). Phenotypic and metabolic variation among spring *Brassica napus* genotypes during heat stress. Crop Pasture Sci. 69, 284–295. doi: 10.1071/CP17259

[B45] Kourani M. Mohareb F. Rezwan F. I. Anastasiadi M. Hammond J. P. (2022). Genetic and physiological responses to heat stress in *Brassica napus* . Front. Plant Sci. 13. doi: 10.3389/fpls.2022.832147 PMC9016328 35449889

[B46] Kumar M. Prusty M. R. Pandey M. K. Singh P. K. Bohra A. Guo B.. (2023). Application of CRISPR/Cas9-mediated gene editing for abiotic stress management in crop plants. Front. Plant Sci. 14). doi: 10.3389/fpls.2023.1157678 PMC10153630 37143874

[B47] Lamaoui M. Jemo M. Datla R. Bekkaoui F. (2018). Heat and drought stresses in crops and approaches for their mitigation. Front. Chem. 6. doi: 10.3389/fchem.2018.00026 PMC5827537 29520357

[B48] Lemoine R. La Camera S. Atanassova R. Dédaldéchamp F. Allario T. Pourtau N.. (2013). Source-to-sink transport of sugar and regulation by environmental factors. Front. Plant Sci. 24. doi: 10.3389/fpls.2013.00272 PMC3721551 23898339

[B49] Li M. Li J. Zhang R. Lin Y. Xiong A. Tan G.. (2022). Combined analysis of the metabolome and transcriptome to explore heat stress responses and adaptation mechanisms in celery (*Apium graveolens* L.). Int. J. Mol. Sci. 23, 3367. doi: 10.3390/ijms23063367 35328788 PMC8950972

[B50] Ljubej V. Radojčić Redovniković I. Salopek-Sondi B. Smolko A. Roje S.. (2021). Chilling and freezing temperature stress differently influence glucosinolates content in *Brassica oleracea* var. *acephala* . Plants (Basel) 10, 1305. doi: 10.3390/plants10071305 34199146 PMC8309204

[B51] Lohani N. Singh M. B. Bhalla P. L. (2020). High temperature susceptibility of sexual reproduction in crop plants. J. Exp. Bot. 71, 555–568. doi: 10.1093/jxb/erz426 31560053

[B52] Long X. He B. Fang Y. Tang C. (2016). Identification and characterization of the glucose-6-phosphate dehydrogenase gene family in the para rubber tree, *Hevea brasiliensis* . Front. Plant Sci. 7. doi: 10.3389/fpls.2016.00215 PMC4766392 26941770

[B53] Love M. I. Huber W. Anders S. (2014). Moderated estimation of fold change and dispersion for RNA-seq data with DESeq2. Genome Biol. 15, 550. doi: 10.1186/s13059-014-0550-8 25516281 PMC4302049

[B54] Marias D. Meinzer F. C. Still C. (2017). Impacts of leaf age and heat stress duration on photosynthetic gas exchange and foliar nonstructural carbohydrates in *Coffea arabica* . Ecol. Evol. 7, 1297–1310. doi: 10.1002/ece3.2017.7.issue-4 28303198 PMC5306013

[B55] Martínez-Ballesta M. del C. Moreno D. A. Carvajal M. (2013). The physiological importance of glucosinolates on plant response to abiotic stress in Brassica. Int. J. Mol. Sci. 14, 11607–11625. doi: 10.3390/ijms140611607 23722664 PMC3709749

[B56] Mathan J. Singh A. Ranjan A. (2020). Sucrose transport in response to drought and salt stress involves ABA-mediated induction of OsSWEET13 and OsSWEET15 in rice. Physiologia Plantarum 171, 620–637. doi: 10.1111/ppl.13210 32940908

[B57] Met Office UK temperature, rainfall and sunshine time series. Available online at: https://www.metoffice.gov.uk/research/climate/maps-and-data/uk-temperature-rainfall-and-sunshine-time-series (Accessed January 10, 2023).

[B58] Mishra S. Chowdhary A. A. Mehrotra S. Srivastava V. (2019). Function of plant heat shock transcription factors in abiotic stress. In Upadhyay S. K. (Ed.), Energy Environ. Sustainability, 113–126. doi: 10.1007/978-981-15-0690-1_6

[B59] Mishra S. K. Tripp J. Winkelhaus S. Tschiersch B. Theres K. Nover L.. (2002). In the complex family of heat stress transcription factors, HsfA1 has a unique role as master regulator of thermotolerance in tomato. Genes Dev. 16, 1555–1567. doi: 10.1101/gad.228802 12080093 PMC186353

[B60] Müller J. L. Krishna P. Foreiter C. (2000). A glucosinolate mutant of *Arabidopsis* is thermosensitive and defective in cytosolic Hsp90 expression after heat stress. Plant Physiol. 123, 949–958. doi: 10.1104/pp.123.3.949 10889243 PMC59057

[B61] Namazkar S. Stockmarr A. Frenck G. Egsgaard H. Terkelsen T. Mikkelsen T.. (2016). Concurrent elevation of CO_2_, O_3_ and temperature severely affects oil quality and quantity in rapeseed. J. Exp. Bot. 67, 4117–4125. doi: 10.1093/jxb/erw180 27222513 PMC5301921

[B62] Narayanan S. Tamura P. J. Roth M. R. Prasad P. V. V. Welti R. (2016). Wheat leaf lipids during heat stress: I. High day and night temperatures result in major lipid alterations. Plant Cell Environ. 39, 787–803. doi: 10.1111/pce.12649 26436679 PMC5102054

[B63] Narayanan S. Zoong-Lwe Z. S. Gandhi N. Welti R. Fallen B. Smith J. R.. (2020). Comparative lipidomic analysis reveals heat stress responses of two soybean genotypes differing in temperature sensitivity. Plants 9, 457. doi: 10.3390/plants9040457 32260392 PMC7238245

[B64] Orlemans K. Barrett D. M. Bosch Suades C. Verkerk R. Dekker M. (2006). Thermal degradation of glucosinolates in red cabbage. Food Chem. 95, 19–29. doi: 10.1016/j.foodchem.2004.12.013

[B65] Osorio S. Ruan Y. L. Fernie A. R. (2014). An update on source-to-sink carbon partitioning in tomato. Front. Plant Sci. 5. doi: 10.3389/fpls.2014.00516 PMC4186278 25339963

[B66] Pacheco-Sangerman F. Gómez-Merino F. C. PeraltaSánchez M. Alcántar-González G. Trejo-Téllez L. I. (2023). Glucosinolates: Structure, classification, biosynthesis and functions in higher plants. Agro Productividad 16 (4), 107–114. doi: 10.32854/agrop.v16i3.2567

[B67] Pasini F. Verardo V. Caboni M. F. D’Antuono L. F. (2012). Determination of glucosinolates and phenolic compounds in rocket salad by HPLC-DAD-MS: Evaluation of *Eruca sativa Mill.* And *Diplotaxis tenuifolia* L. genetic resources. Food Chem. 133, 1025–1033. doi: 10.1016/j.foodchem.2012.01.021

[B68] Rao S.-Q. Chen X.-Q. Wang K.-H. Zhu Z.-J. Yang J. Zhu B. (2021). Effect of short-term high temperature on the accumulation of glucosinolates in *Brassica rapa* . Plant Physiol. Biochem. 161, 222–233. doi: 10.1016/j.plaphy.2021.02.013 33639590

[B69] Rask L. Andréasson E. Ekbom B. Eriksson S. Pontoppidan B. Meijer J. (2000). Myrosinase: gene family evolution and herbivore defense in Brassicaceae. Plant Mol. Biol. 42, 93–113. doi: 10.1023/A:1006380021658 10688132

[B70] Rasmusson L. M. Buapet P. George R. Gullström M. Gunnarsson P. C. B. Björk M. (2020). Effects of temperature and hypoxia on respiration, photorespiration, and photosynthesis of seagrass leaves from contrasting temperature regimes. J. Norkko (Ed.), ICES. J. Marine Sci. 77, 2056–2065. doi: 10.1093/icesjms/fsaa093

[B71] Ren Y. Zhu J. Zhang H. Lin B. Hao P. Hua S. (2022). Leaf Carbohydrate Metabolism Variation Caused by Late Planting in Rapeseed (*Brassica napus* L.) at Reproductive Stage. Plants 11, 1696. doi: 10.3390/plants11131696 35807649 PMC9268982

[B72] Rhee J.-H. Choi S. Lee J.-E. Hur O.-S. Ro N.-Y. Hwang A.-J.. (2020). Glucosinolate content in brassica genetic resources and their distribution pattern within and between inner, middle, and outer leaves. Plants 9, 1421. doi: 10.3390/plants9111421 33114129 PMC7690824

[B73] Rong Y. Li T. Liu X. Shi S. Wang X. Tu P. (2021). A transaldolase from Aquilaria Sinensis involves in ABA-mediated seed germination and root growth. doi: 10.21203/rs.3.rs-234062/v1

[B74] Scafaro A. P. Fan Y. Posch B. C. Garcia A. Coast O. Atkin O. K. (2021). Responses of leaf respiration to heatwaves. Plant Cell Environ. 44, 2090–2101. doi: 10.1111/pce.14018 33534189

[B75] Seneviratne S. I. Nicholls N. Easterling D.Goodess C. Kanae S. Kossin J.. (2012). “Changes in climate extremes and their impacts on the natural physical environment,” in Managing the Risks of Extreme Events and Disasters to Advance Climate Change Adaptation. Ed. Field C. B. (Cambridge University Press, Cambridge, UK, and New York, NY, USA), 109–230. A Special Report of Working Groups I and II of the Intergovernmental Panel on Climate Change (IPCC).

[B76] Seth R. Maritim T. K. Parmar R. Sharma R. K. (2021). Underpinning the molecular programming attributing heat stress associated thermotolerance in tea (*Camellia sinensis* (L.) O. Kuntze). Hortic. Res. 8. doi: 10.1038/s41438-021-00532-z PMC8087774 33931616

[B77] Song J. M. Guan Z. Hu J. Guo C. Yang Z. Wang S.. (2020). Eight high-quality genomes reveal pan-genome architecture and ecotype differentiation of *Brassica napus* . Nat. Plants 6, 34–45. doi: 10.1038/s41477-019-0577-7 31932676 PMC6965005

[B78] Sun F. Fan G. Hu Q. Zhou Y. Guan M. Tong C.. (2017). The high quality genome of *Brassica napus* cultivar ‘ZS11’ reveals the introgression history in semi-winter morphotype. Plant J. 92, 452–468. doi: 10.1111/tpj.13669 28849613

[B79] Sun X. Zhu J. Li X. Li Z. Han L. Luo H. (2020). AsHSP26.8a, a creeping bentgrass small heat shock protein integrates different signaling pathways to modulate plant abiotic stress response. BMC Plant Biol. 20, 184. doi: 10.1186/s12870-020-02369-5 32345221 PMC7189581

[B80] Suwa R. Hakata H. Hara H. El-Shemy H. A. Adu-Gyamfi J. J. Nguyen N. T.. (2010). High temperature effects on photosynthate partitioning and sugar metabolism during ear expansion in maize (Zea mays L.) genotypes. Plant Physiol. Biochem. 48, 124–130. doi: 10.1016/j.plaphy.2009.12.010 20106675

[B81] Tang Y. Zhang G. Jiang X. Shen S. Guan M. Tang Y.. (2023). Genome-wide association study of glucosinolate metabolites (mGWAS) in *Brassica napus* L. Plants 12, 639. doi: 10.3390/plants12030639 36771722 PMC9921834

[B82] Touw A. J. Verdecia Mogena A. Maedicke A. Sontowski R. van Dam N. M. Tsunoda T. (2020). Both biosynthesis and transport are involved in glucosinolate accumulation during root-herbivory in *Brassica rapa* . Front. Plant Sci. 10. doi: 10.3389/fpls.2019.01653 PMC6970201 31998341

[B83] USDA. (2025). Oilseeds: World markets and trade. United States Department of Agriculture, Foreign Agricultural Service. https://apps.fas.usda.gov/psdonline/circulars/oilseeds.pdf

[B84] Valente Pereira F. M. V. Rosa E. Fahey J. W. Stephenson K. K. Carvalho R. Aires A. (2002). Influence of temperature and ontogeny on the Levels of glucosinolates in broccoli (*Brassica oleracea* Var. italica) sprouts and their effect on the induction of mammalian phase 2 enzymes. J. Agric. Food Chem. 50, 6239–6244. doi: 10.1021/jf020309x 12358509

[B85] Van Dongen J. T. Gupta K. J. Ramírez-Aguilar S. J. Araújo W. L. Nunes-Nesi A. Fernie A. R. (2011). Regulation of respiration in plants: A role for alternative metabolic pathways. J. Plant Physiol. 168, 1434–1443. doi: 10.1016/j.jplph.2010.11.004 21185623

[B86] Wahid A. Gelani S. Ashraf M. Foolad M. R. (2007). Heat tolerance in plants: An overview. Environ. Exp. Bot. 61, 199–223. doi: 10.1016/j.envexpbot.2007.05.011

[B87] Wang L. Ma K. B. Lu Z. G. Ren S. X. Jiang H. R. Cui J. W.. (2020). Differential physiological, transcriptomic and metabolomic responses of Arabidopsis leaves under prolonged warming and heat shock. BMC Plant Biol. 20. doi: 10.1186/s12870-020-2292-y PMC7036190 32087683

[B88] Wang J. Yu H. Zhao Z. Sheng X. Shen Y. Gu H. (2019). Natural Variation of Glucosinolates and Their Breakdown Products in Broccoli (*Brassica oleracea* var. italica) Seeds. J. Agric. Food Chem. 67, 12528–12537. doi: 10.1021/acs.jafc.9b06533 31631662

[B89] Way D. A. Yamori W. (2014). Thermal acclimation of photosynthesis: On the importance of adjusting our definitions and accounting for thermal acclimation of respiration. Photosynthesis Res. 119, 89–100. doi: 10.1007/s11120-013-9873-7 23812760

[B90] WeatherOnline Max temperature London - Observations 05.2017 interval: 02 Weeks | United Kingdom Weather History. Available online at: https://www.weatheronline.co.uk/weather/maps/city (Accessed January 10, 2023).

[B91] Xalxo R. Yadu B. Chandra J. Chandrakar V. Keshavkant S. (2020). Alteration in carbohydrate metabolism modulates thermotolerance of plant under heat stress. In Wani S. H. Wani S. S. Bhat A. S. A. (Eds.), Heat Stress Tolerance Plants, 77–115. doi: 10.1002/9781119432401.ch5

[B92] Yu E. Fan C. Yang Q. Li X. Wan B. Dong Y.. (2014). Identification of heat responsive genes in *Brassica napus* siliques at the seed-filling stage through transcriptional profiling. PloS One 9, e101914. doi: 10.1371/journal.pone.0101914 25013950 PMC4094393

[B93] Yuenyong W. Sirikantaramas S. Qu L. J. Buaboocha T. (2019). Isocitrate lyase plays important roles in plant salt tolerance. BMC Plant Biol. 19, 472. doi: 10.1186/s12870-019-2086-2 31694539 PMC6833277

[B94] Zafar I. Hussain A. I. Fatima T. Abdullah Alnasser S. M. Ahmad A. (2022). Inter-varietal variation in phenolic profile, sugar contents, antioxidant, anti-proliferative and antibacterial activities of selected brassica species. Appl. Sci. 12, 5811. doi: 10.3390/app12125811

[B95] Zafar S. A. Zaidi S. S. E. A. Gaba Y. Singla-Pareek S. L. Dhankher O. P. Li X.. (2020). Engineering abiotic stress tolerance via CRISPR/Cas-mediated genome editing. J. Exp. Bot. 71, 470–479. doi: 10.1093/jxb/erz476 31644801

[B96] Zandalinas S. I. Rivero R. M. Martínez V. Gómez-Cadenas A. Arbona V. (2016). Tolerance of citrus plants to the combination of high temperatures and drought is associated to the increase in transpiration modulated by a reduction in abscisic acid levels. BMC Plant Biol. 16, 1–16. doi: 10.1186/s12870-016-0791-7 27121193 PMC4848825

[B97] Zhang S. S. Yang H. Ding L. Song Z. T. Ma H. Chang F.. (2017). Tissue-specific transcriptomics reveals an important role of the unfolded protein response in maintaining fertility upon heat stress in Arabidopsis. Plant Cell 29, 1007–1023. doi: 10.1105/tpc.16.00916 28442596 PMC5466030

[B98] Zheng G. Tian B. Zhang F. Tao F. Li W. (2011). Plant adaptation to frequent alterations between high and low temperatures: Remodelling of membrane lipids and maintenance of unsaturation levels. Plant Cell Environ. 34, 1431–1442. doi: 10.1111/j.1365-3040.2011.02341.x 21486310 PMC3980542

[B99] Zhou L. Yan T. Chen X. Li Z. Wu D. Hua S.. (2018). Effect of high night temperature on storage lipids and transcriptome changes in developing seeds of oilseed rape. J. Exp. Bot. 69, 1721–1733. doi: 10.1093/jxb/ery004 29420740 PMC5888911

[B100] Zoong Lwe Z. Sah S. Persaud L. Li J. Gao W. Reddy K. R.. (2021). Alterations in the leaf lipidome of Brassica carinata under high-temperature stress. BMC Plant Biol. 21, 404. doi: 10.1186/s12870-021-03189-x 34488625 PMC8419912

