# Supplementary material for: Prolonged heat stress in Brassica napus during flowering negatively impacts yield and alters glucosinolate and sugars metabolism
Source: Front Plant Sci. 2025 May 9;16:1507338. doi: 10.3389/fpls.2025.1507338 (PMC12098335; doi:10.3389/fpls.2025.1507338)
Supplement: Supplementary file 1 [file DataSheet1.docx]

**Prolonged heat stress in *Brassica napus* during flowering negatively impacts yield and alters glucosinolate and sugar metabolism**

Supplementary Figures and Tables:


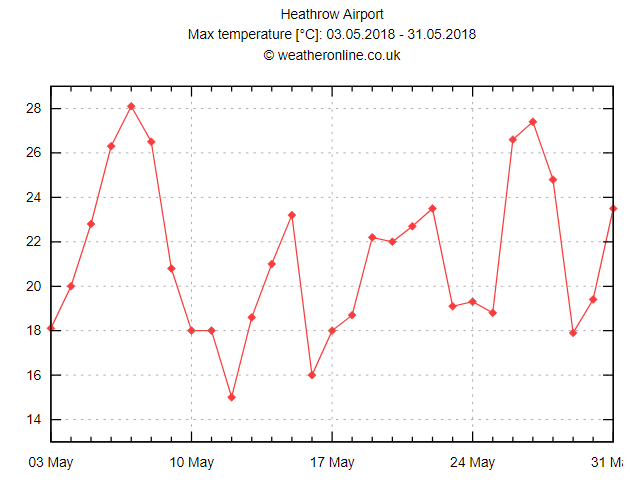

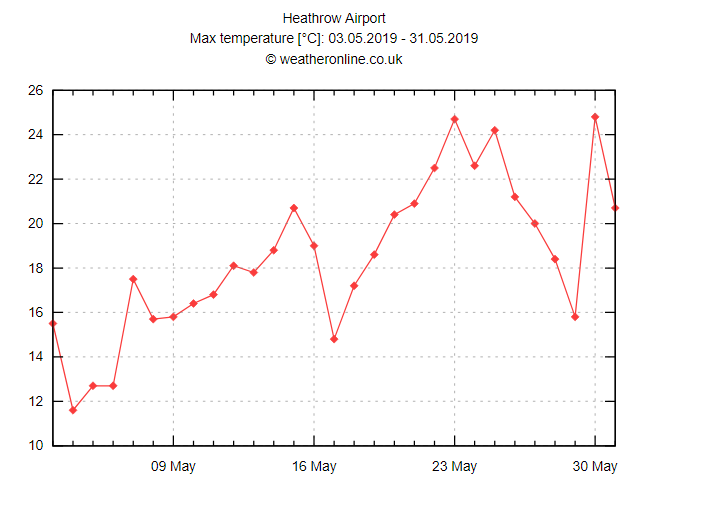

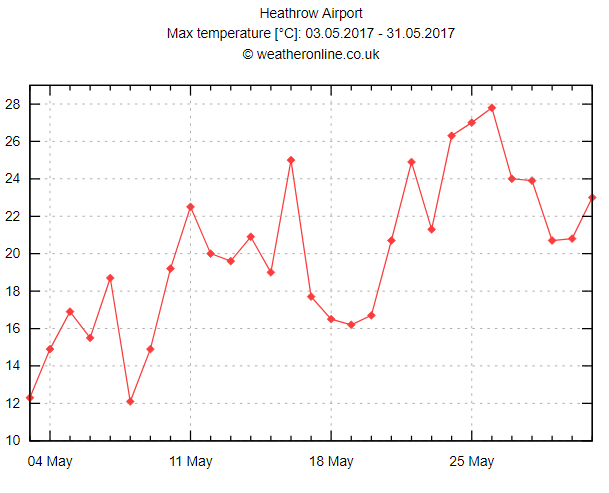

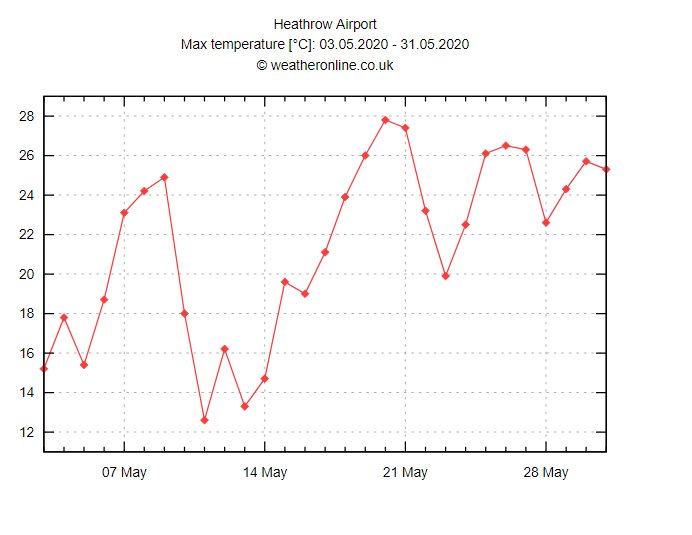


Figure S1. Maximum daily temperature (°C) recorded at Heathrow airport during May 2017, May 2018, May 2019 and May 2020. Records show an increase in the frequency of temperature surges during May.


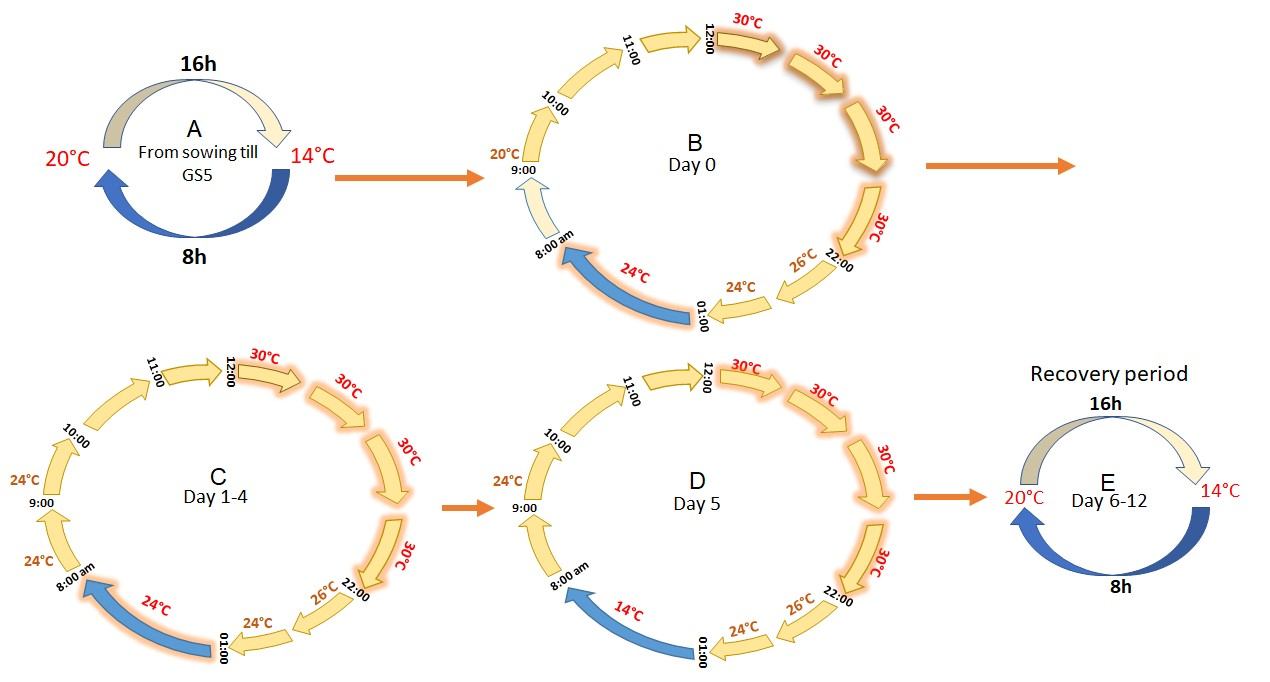


Figure S2. Heat treatment experimental design. To mimic a heatwave, the temperature in the heat treatment cabinet was increased gradually from 20 °C to 30 °C between 9:00 h and 12:00 h. The temperature was held at 30 °C until 22:00 h (B), before it was dropped gradually and maintained at 24 °C until 8:00 h of the next day (B). This cycle of gradual increase (day) and decrease (night) of temperature was held for the next 5 days (C). On the 6^th^ day of treatment, the temperature was gradually decreased to 20 °C/14 °C day/night cycle (D) and held for a recovery period of 7 days (E).

Figure S3. Percentage of aligned reads per sample. Around 90%- 95.49% total reads per sample were mapped to the B. napus cv. Westar v0 reference genome.


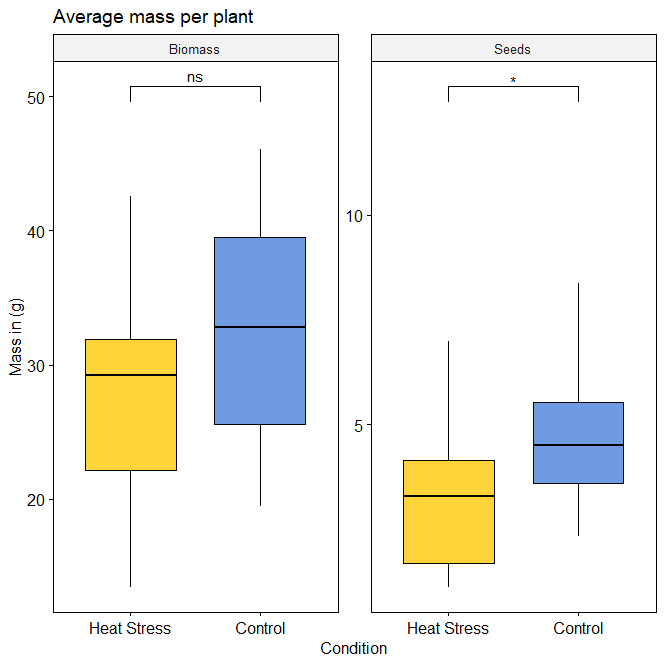


Figure S4. Above ground total biomass and seed yield per plant at maturity. Heat-treated plants (yellow) exhibited less total biomass and seed weight relative to control plants (blue).

*

**


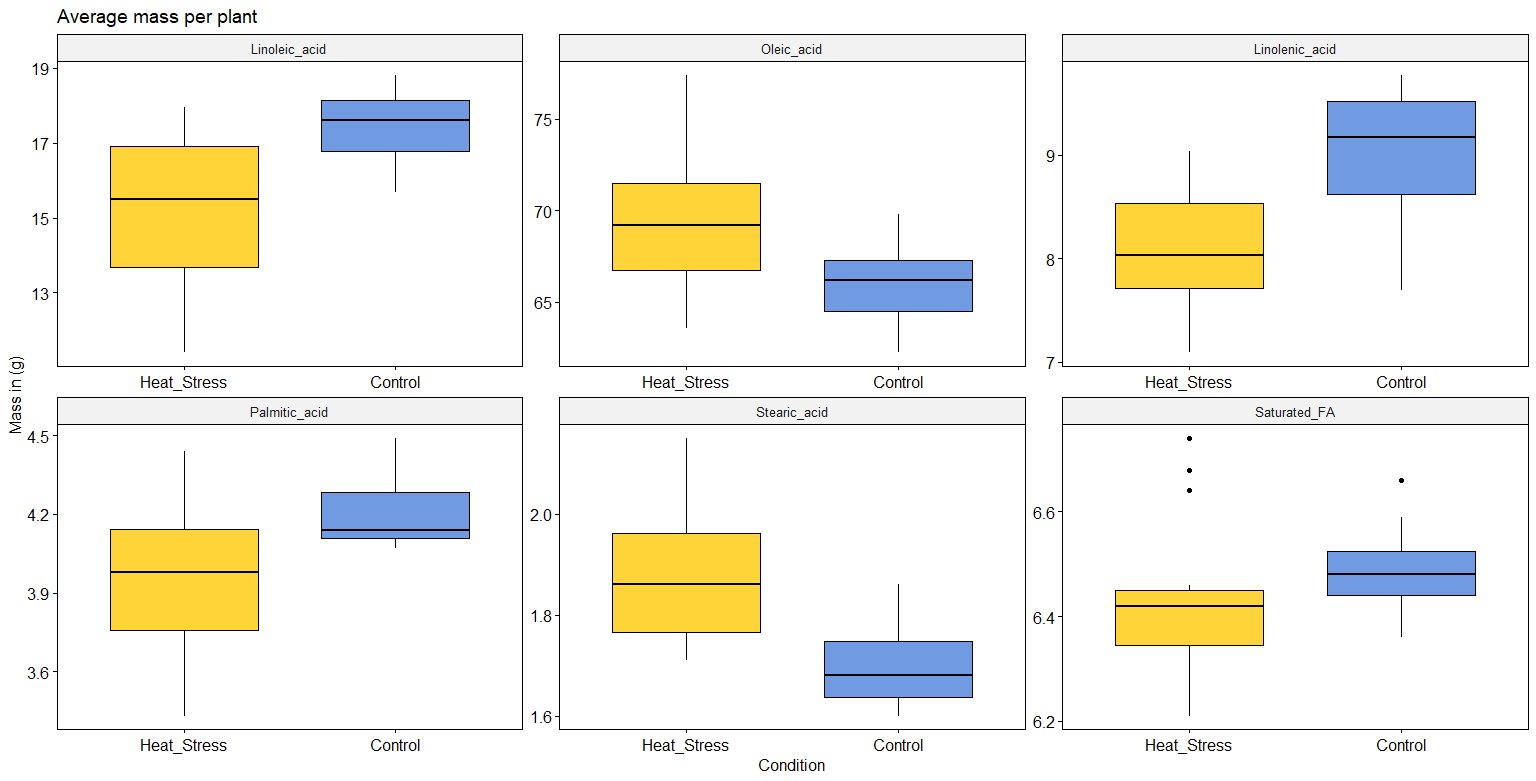


**

*

***

***

*

Figure S5. Fatty acid content in seeds per plant. Seeds from heat-treated plants (yellow) exhibited significant decrease in Linoleic Acid, Linolenic Acid and Palmitic Acid concentrations, but an increase in Oleic Acid and Stearic Acid concentrations compared to seeds from control plants (blue).


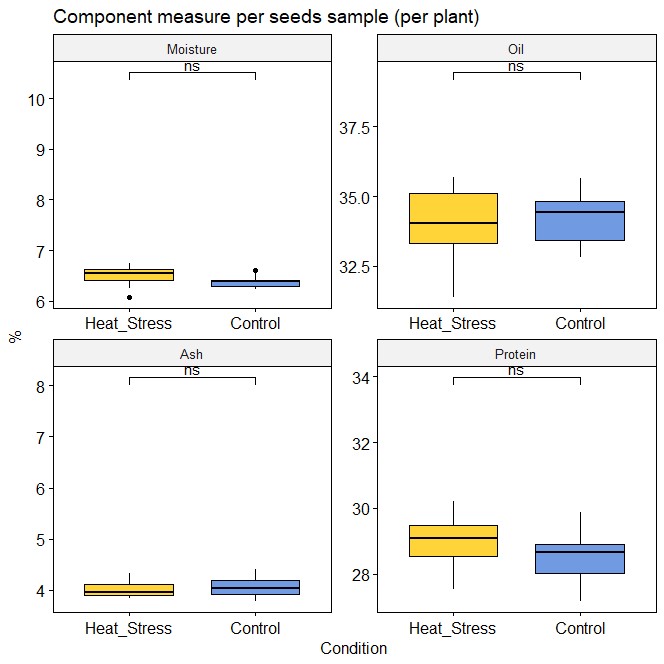


Figure S6. Moisture, oil, ash and protein content in seeds per plant.


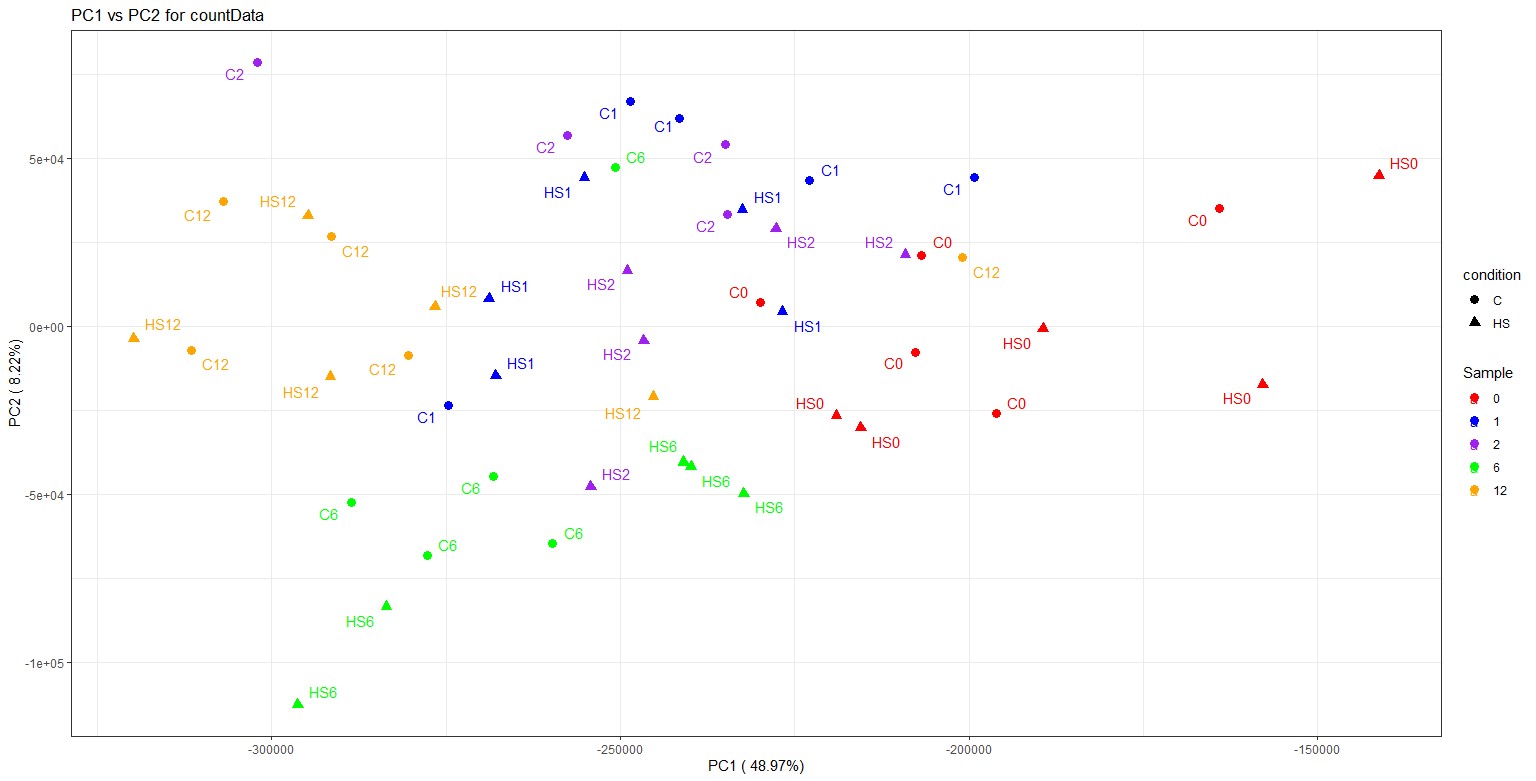


Figure S7. Principle component analysis of the control and heat treatment samples: Samples distribution in PC1 and PC2.


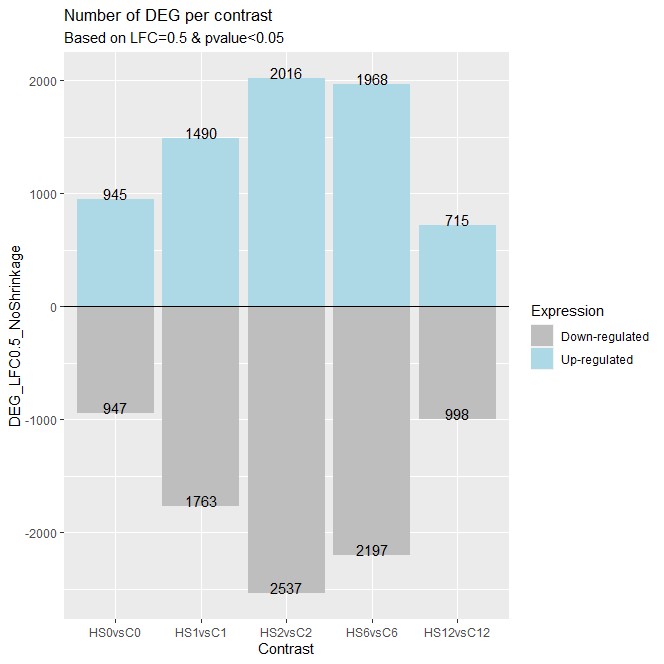

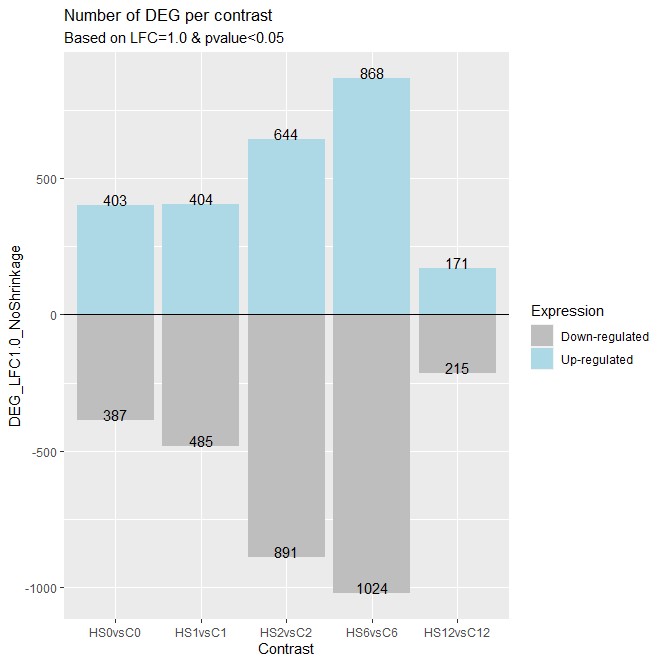


Figure S8. Pairwise comparison of the number of DEG between heat treatment and control at each timepoint based on LFC =0.5 (left) & LFC = 1.0 (right) and p-value ≤0.05. HS0vsC0, HS1vsC1, HS2vsC2, HS6vsC6, and HS12vsC12 contrasts correspond to 0, 1, 2 days after treatment (DAT) & 7 days of recovery (DOR) respectively.

Figure S9. Correlation analysis of the results obtained from RNA-seq and qt-PCR: HSP20 and SS5 exhibited similar expression profiles between RT-qPCR and RNA-seq data with correlation coefficients of r=0.86, and r=0.68 respectively.


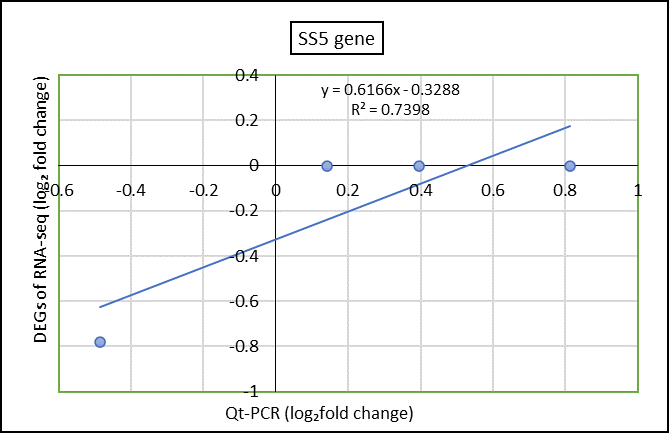

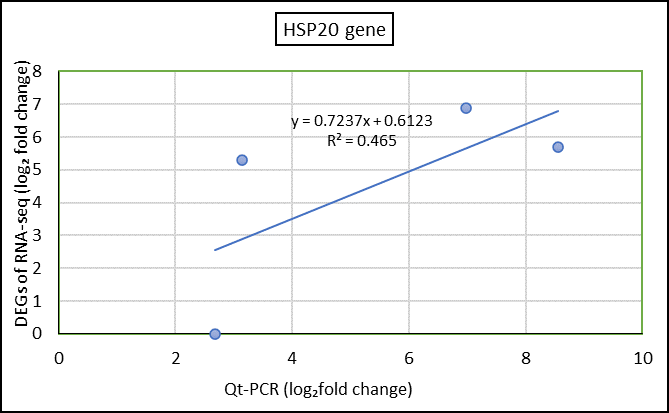


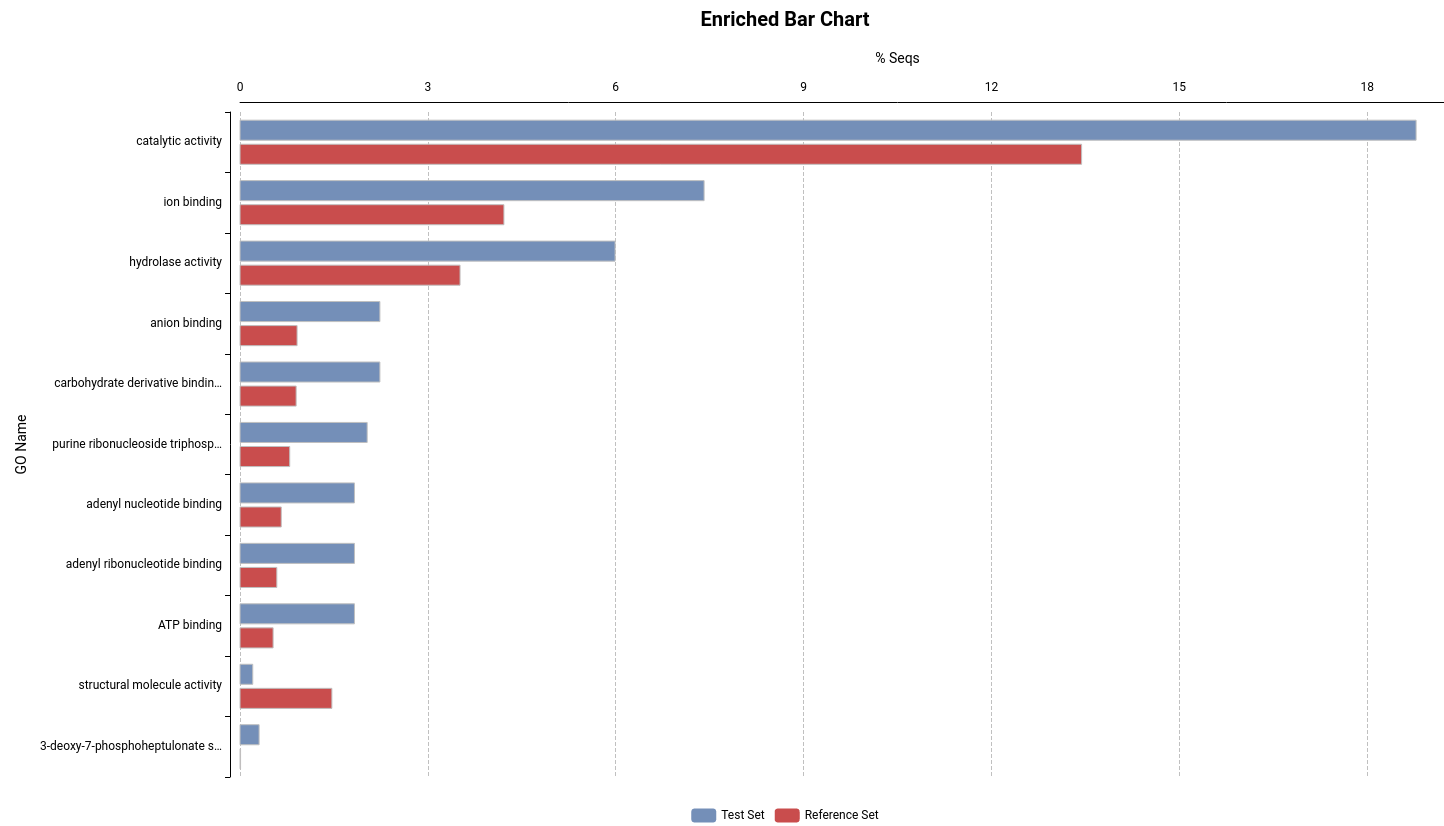


Figure S10. Enriched GO terms at 0 DAT, as identified by GO enrichment analysis.


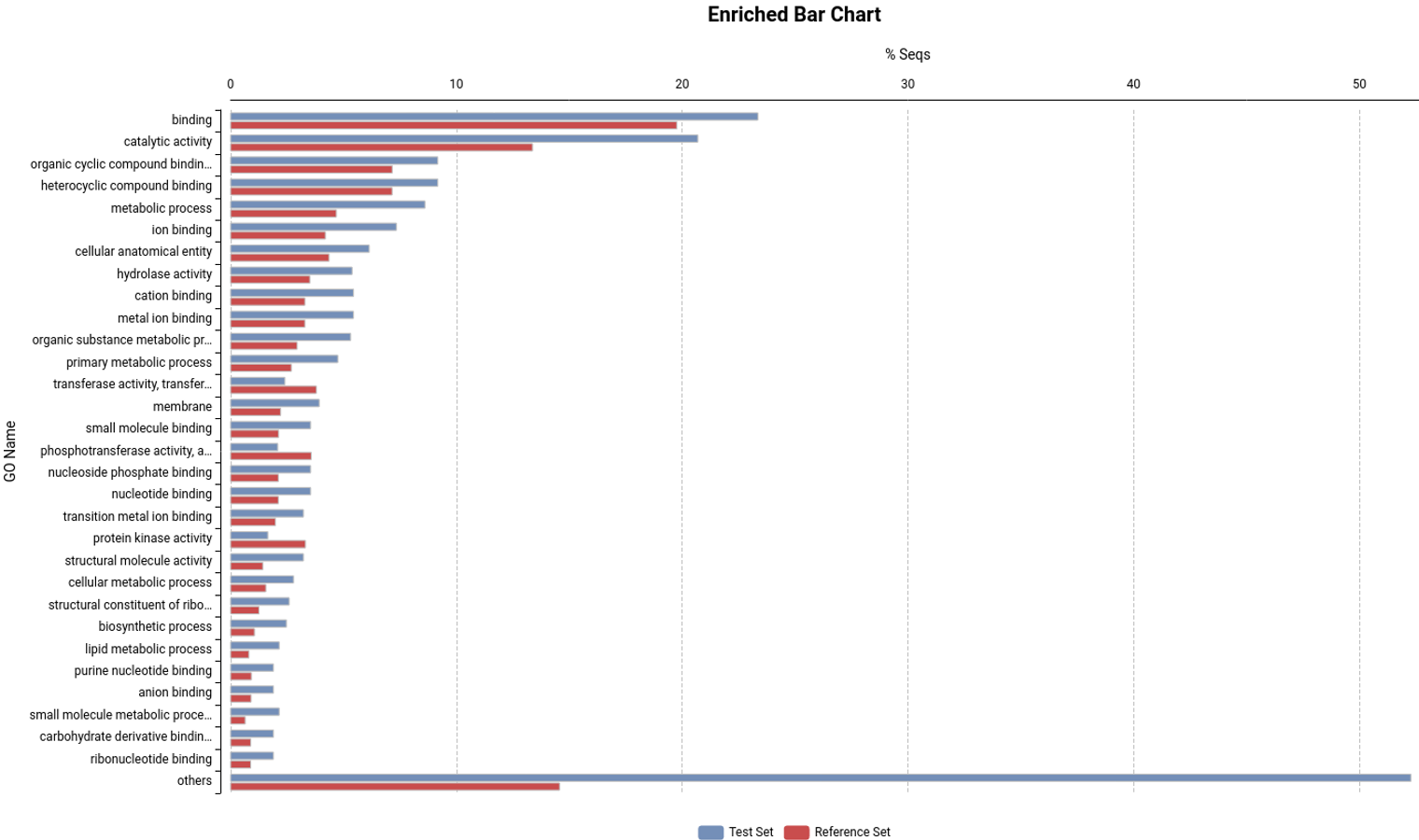


Figure S11. Enriched GO terms at 1 DAT, as identified by GO enrichment analysis.


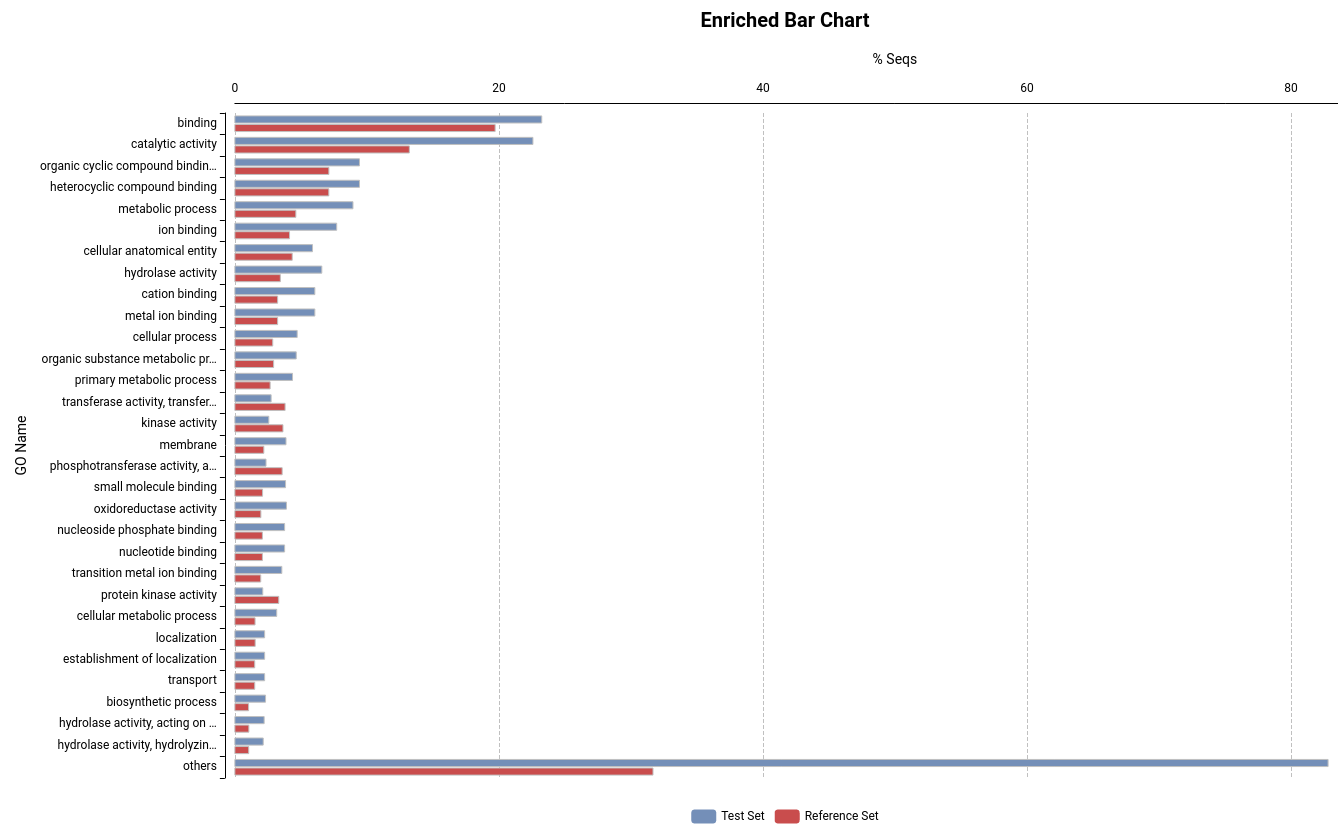


Figure S12. Enriched GO terms at 2 DAT, as identified by GO enrichment analysis.


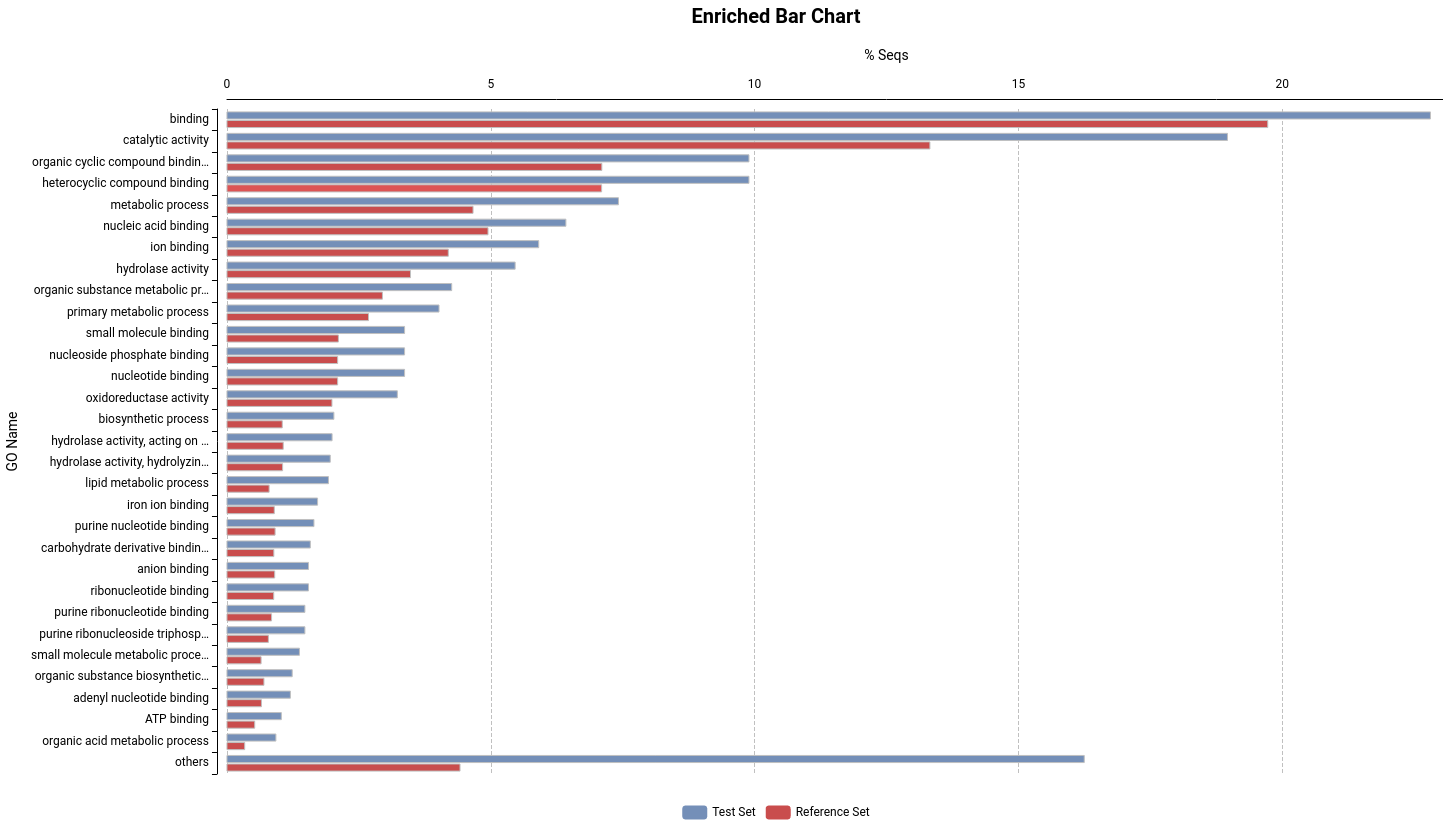


Figure S13. Enriched GO terms at 1 DOR, as identified by GO enrichment analysis.


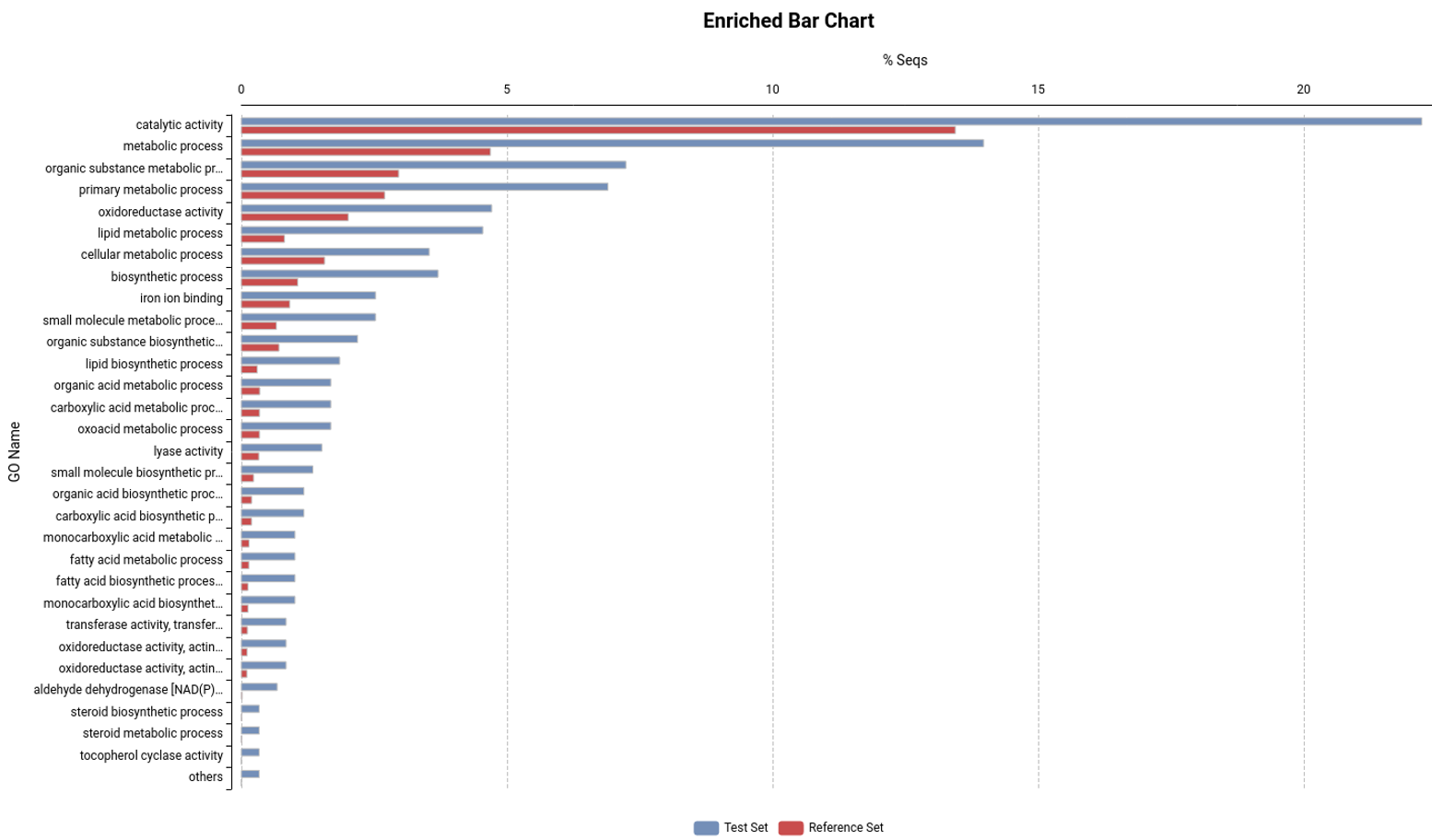


Figure S14. Enriched GO terms at 7 DOR, as identified by GO enrichment analysis.

Figure S7. GO terms distribution for the biological processes (BP), molecular functions (MF) and cellular compartments

(CC) at different timepoints as identified by GO enrichment analysis.

Figure S15. Enzyme code distribution at different timepoints as identified by GO enrichment analysis.


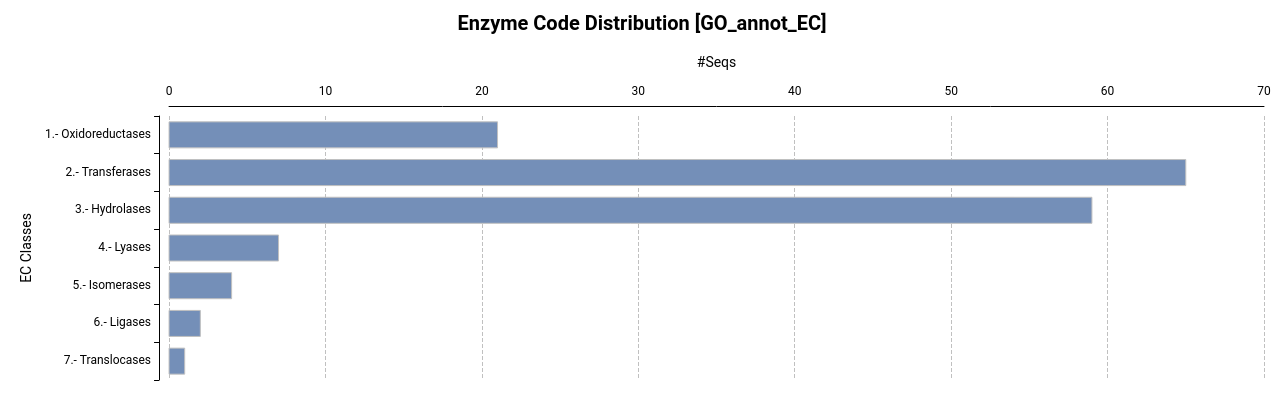

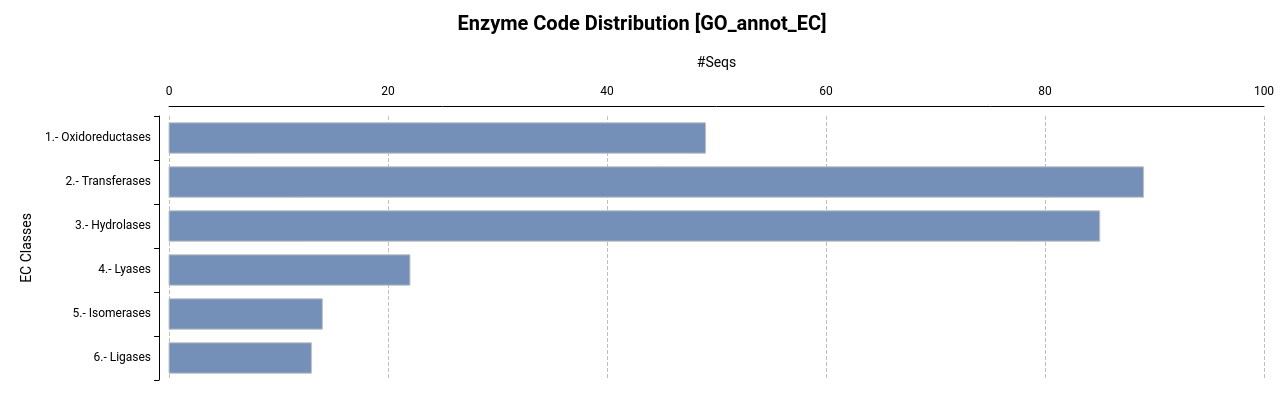

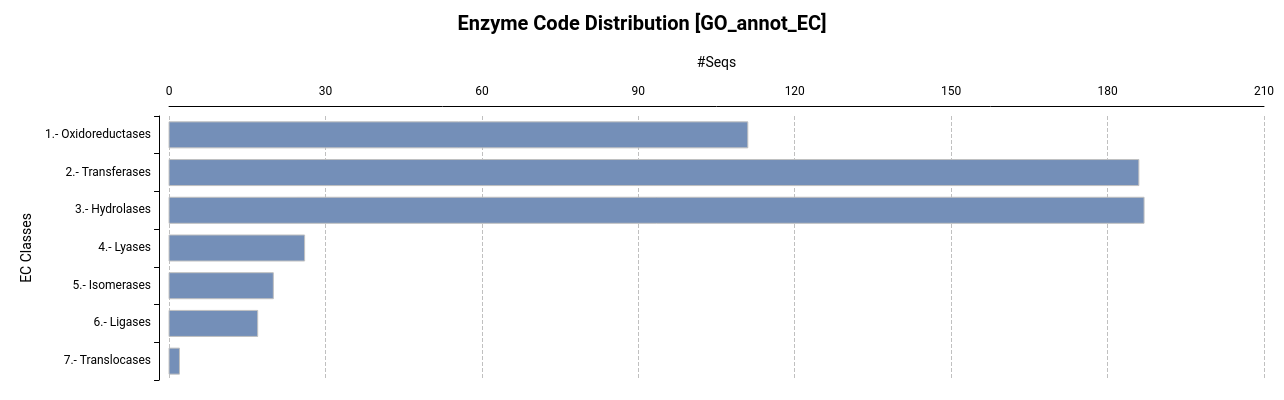

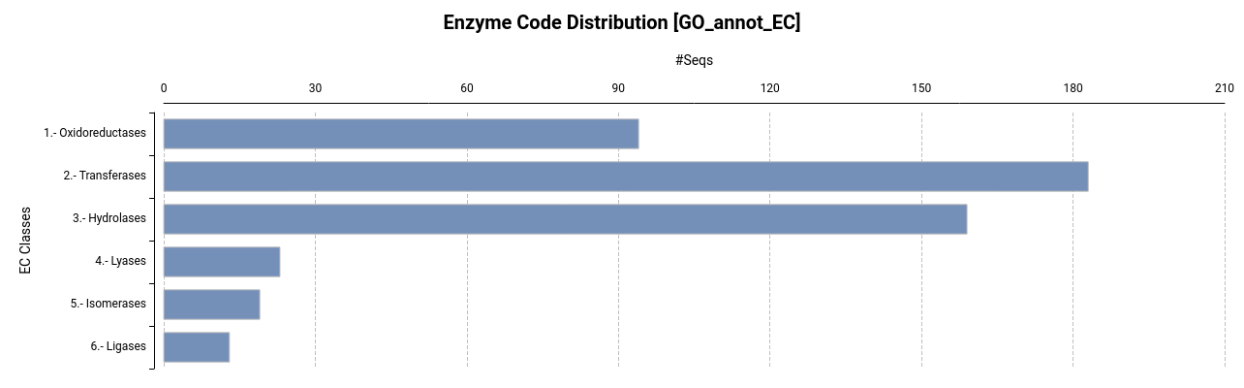

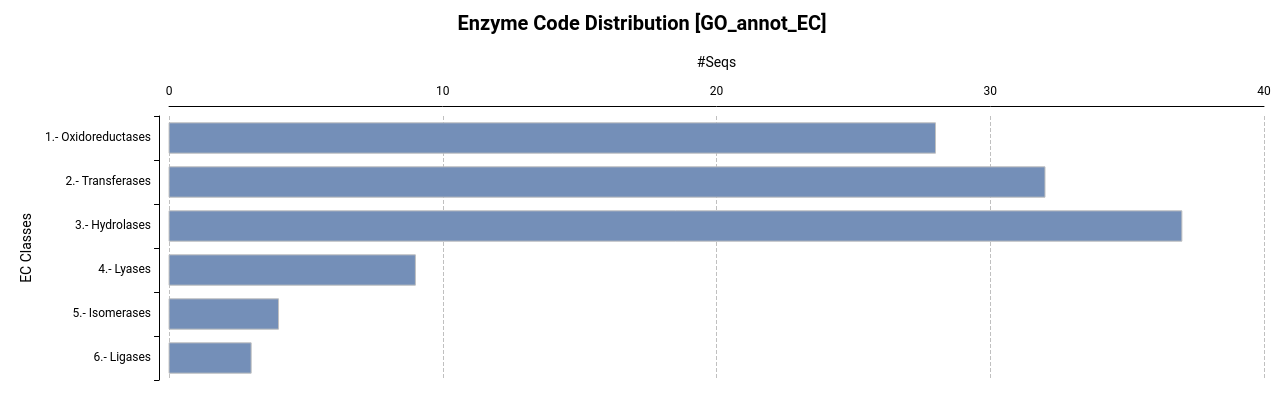


**Timepoint 0**

**Timepoint 1**

**Timepoint 2**

**Timepoint 6**

**Timepoint 12**


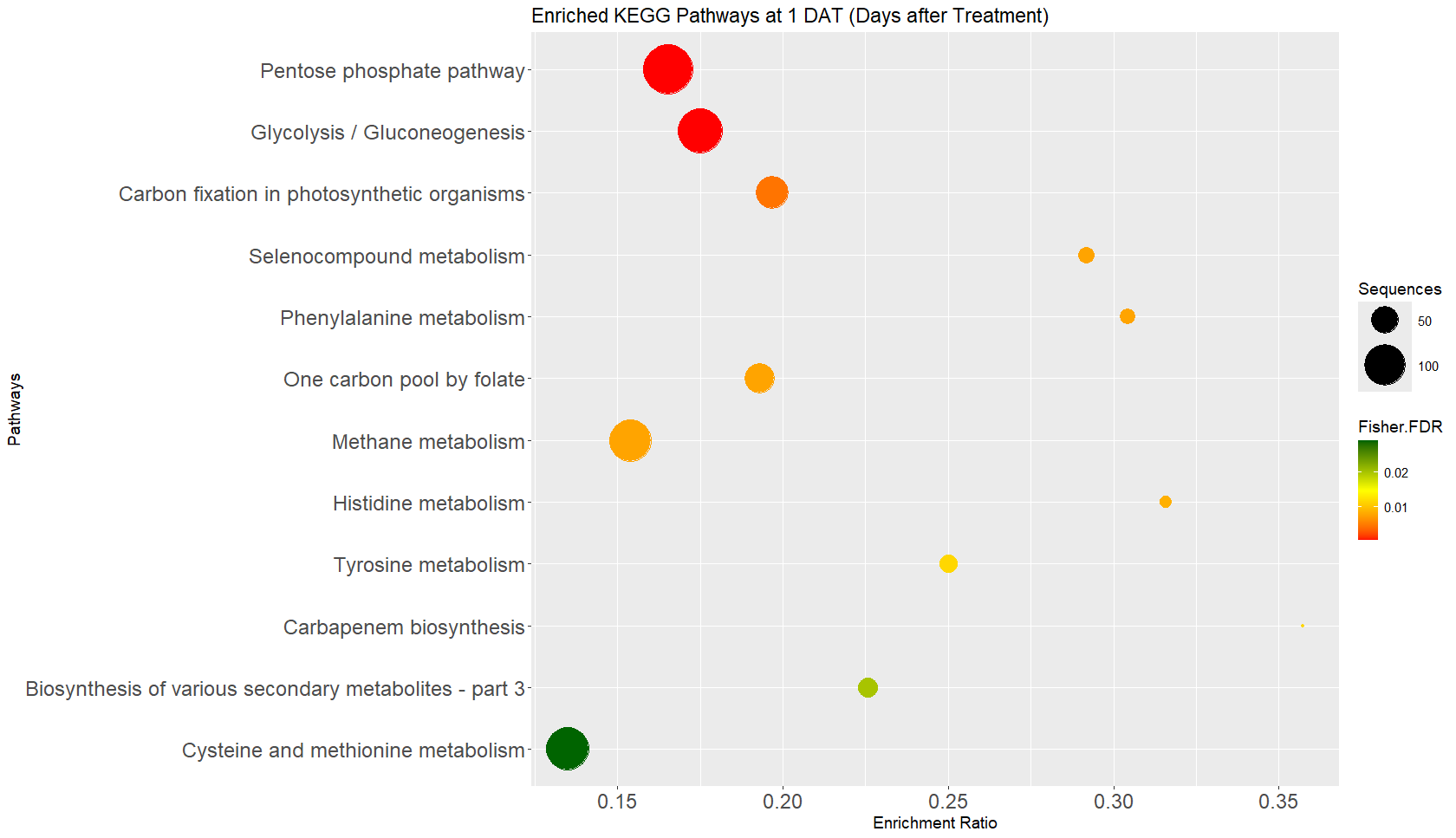


Figure S16. The top KEGG pathways and their corresponding number of genes at 1 DAT at p-value ≤ 0.05. The x-axis represents the enrichment factor. The y-axis represents KEGG pathways. The circle represents the number of genes mapped to pathways and the red to green bar indicates the significance.


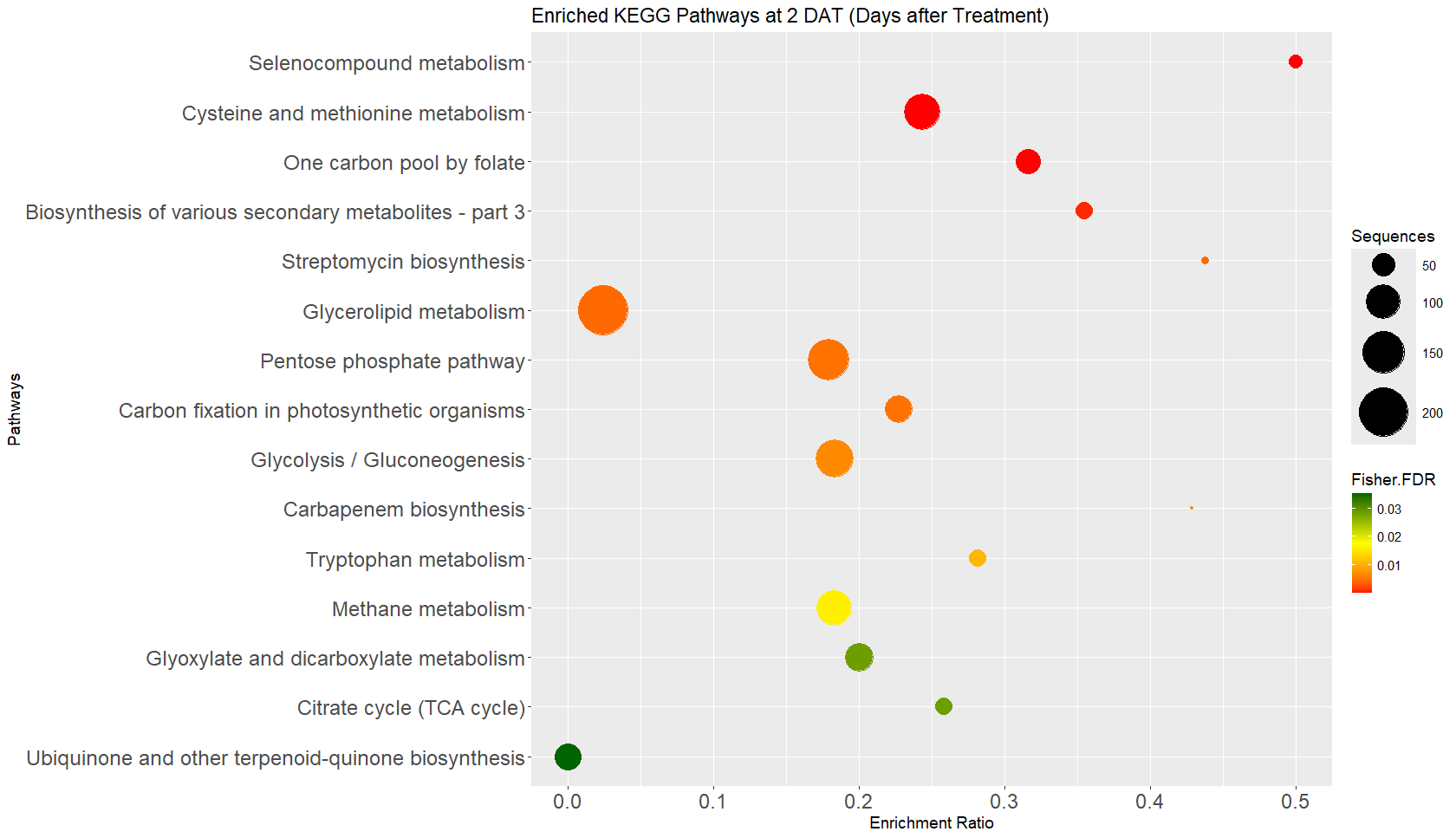


Figure S17. The top KEGG pathways and their corresponding number of genes at 2 DAT at p-value ≤ 0.05. The x-axis represents the enrichment factor. The y-axis represents KEGG pathways. The circle represents the number of genes mapped to pathways and the red to green bar indicates the significance.


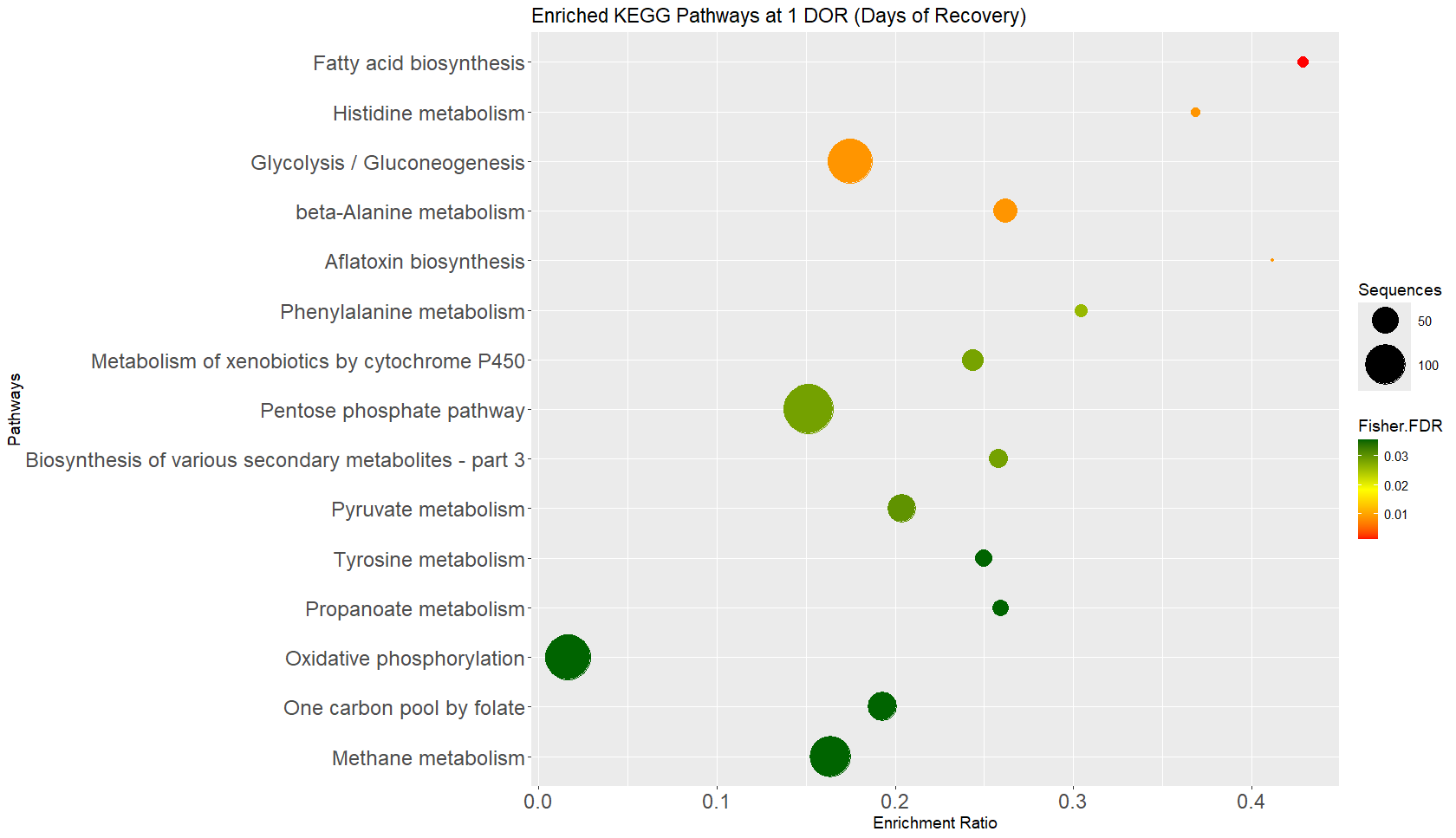


Figure S18. The top KEGG pathways and their corresponding number of genes at 1 DOR at p-value ≤ 0.05. The x-axis represents the enrichment factor. The y-axis represents KEGG pathways. The circle represents the number of genes mapped to pathways and the red to green bar indicates the significance.


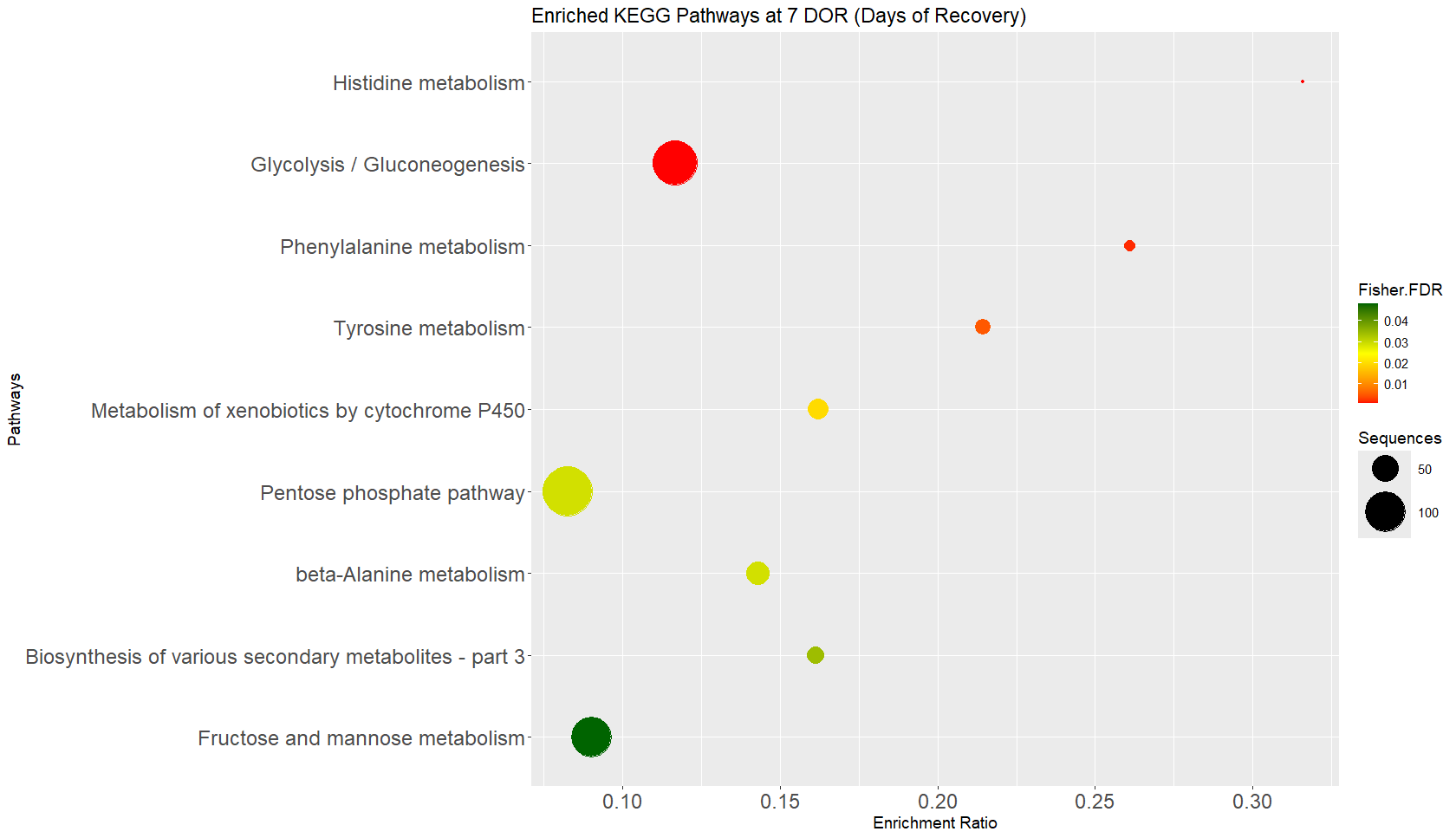


Figure S19. The top KEGG pathways and their corresponding number of genes at 7 DOR at p-value ≤ 0.05. The x-axis represents the enrichment factor. The y-axis represents KEGG pathways. The circle represents the number of genes mapped to pathways and the red to green bar indicates the significance.


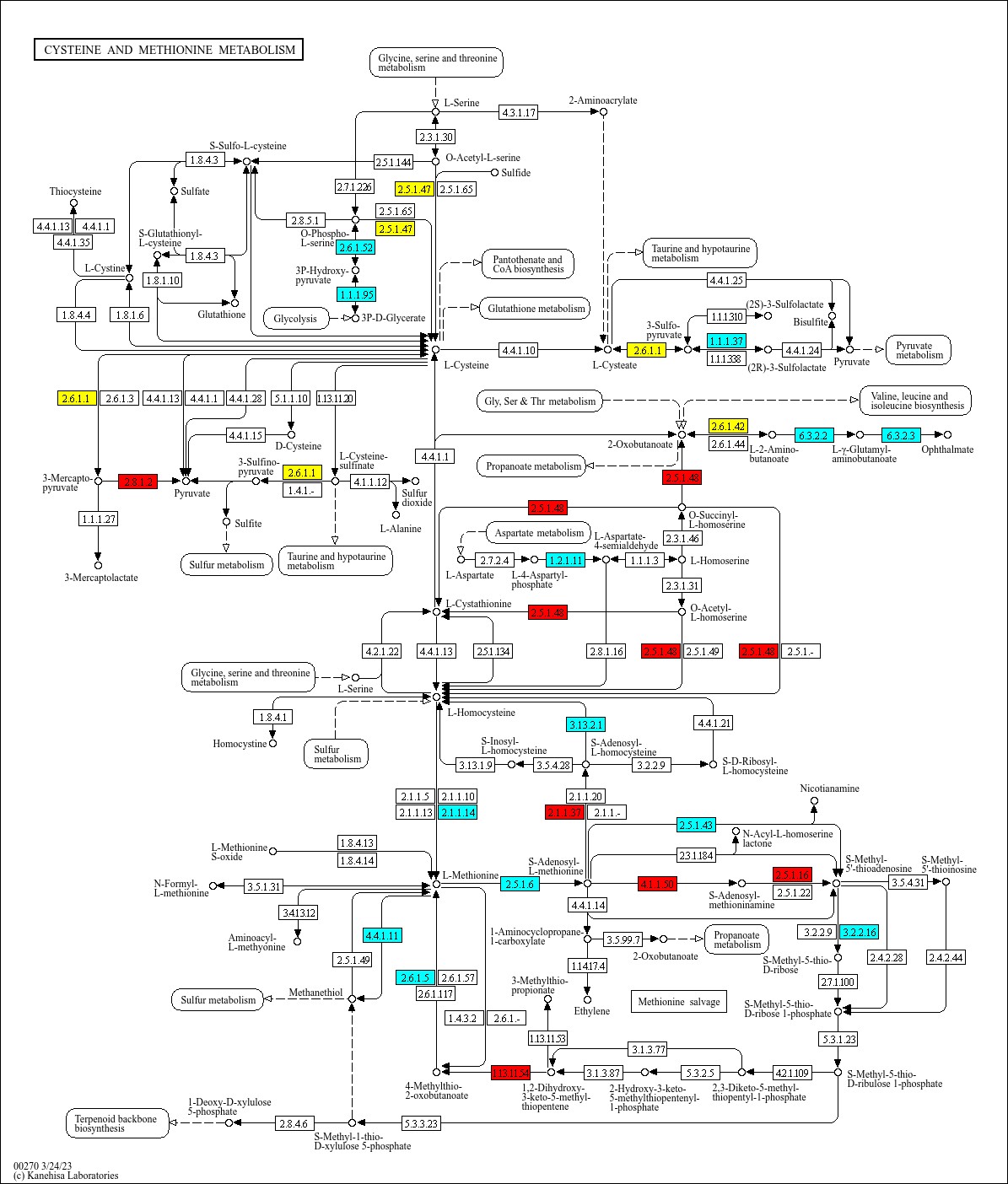


Figure S20. KEGG pathway for Cysteine and Methionine metabolism at 2 DAT.

*Blue indicates down regulated genes; red indicates up regulated genes and yellow indicates a combination of both—some genes are upregulated, while others are downregulated.


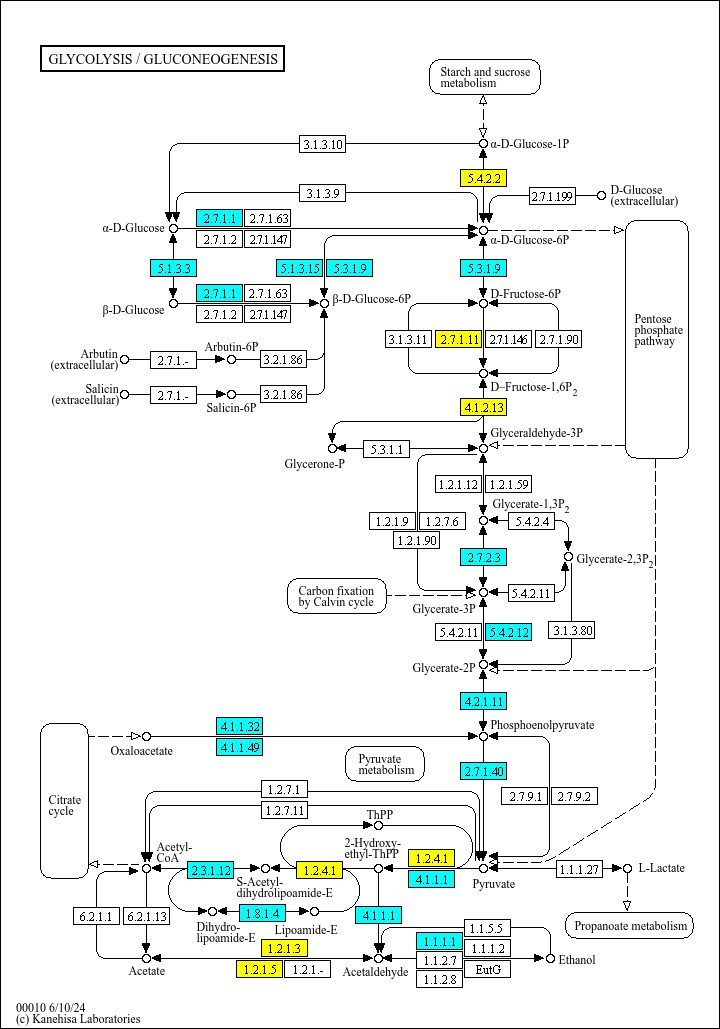


Figure S21. KEGG pathway for Glycolysis at 2 DAT. *Blue indicates down regulated genes and yellow indicates a combination of both—some genes are upregulated, while others are downregulated.


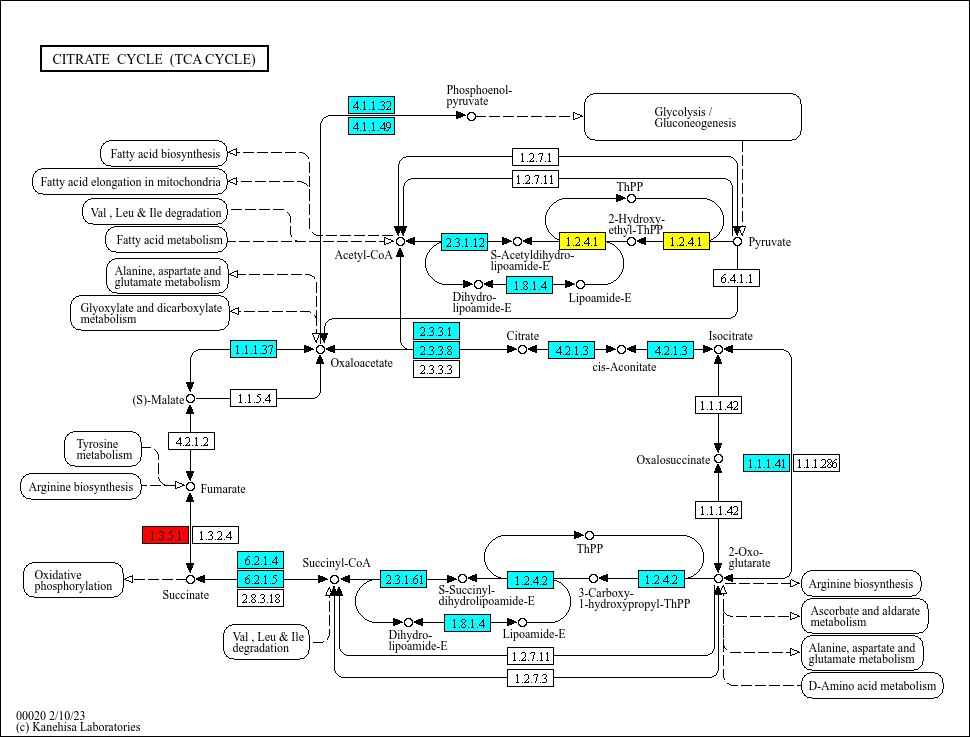


Figure S22. KEGG pathway for Citrate Cycle at 2 DAT. *Blue indicates down regulated genes; red indicates up regulated genes and yellow indicates a combination of both—some genes are upregulated, while others are downregulated.


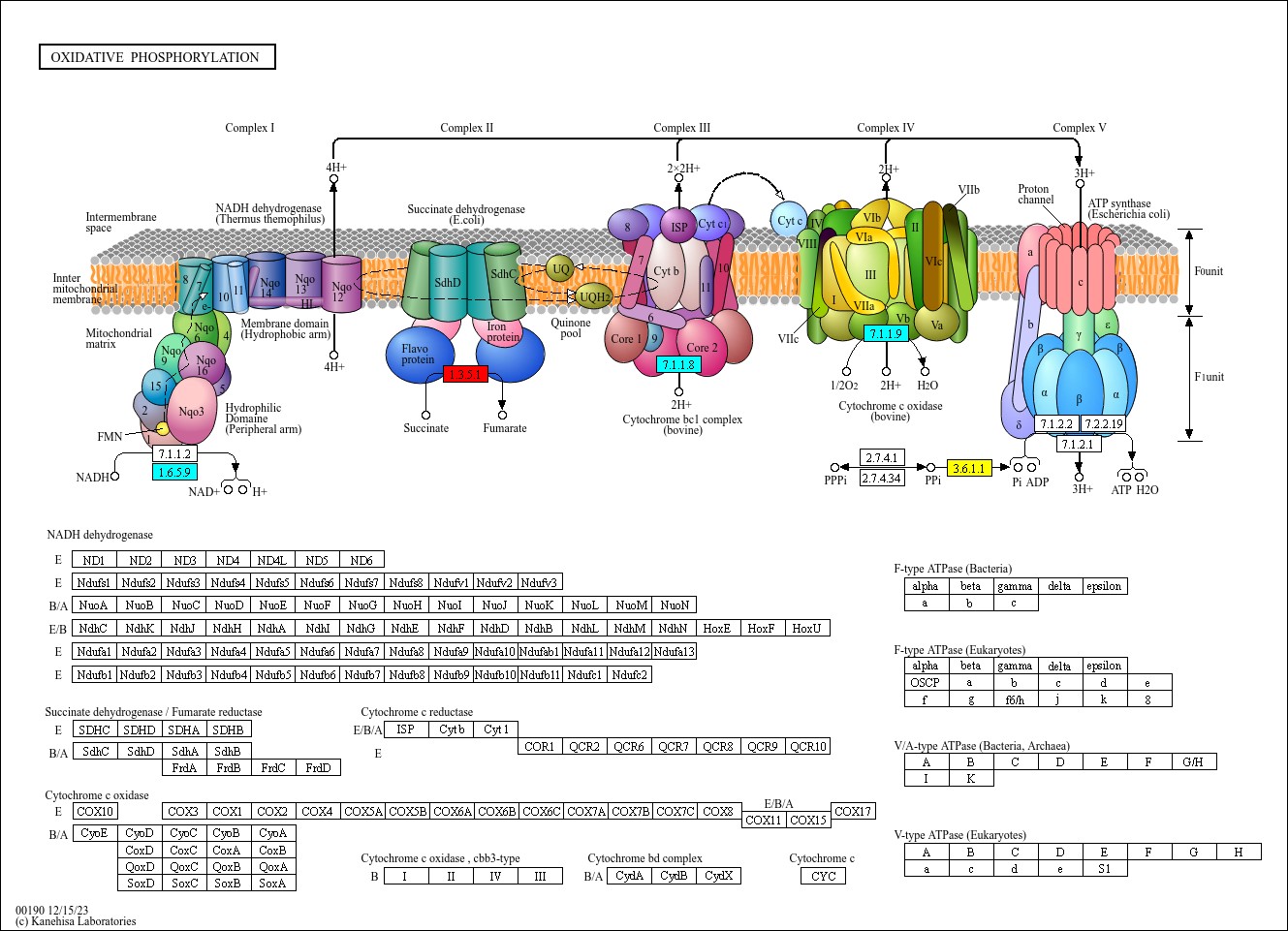
Figure S23. KEGG pathway for Oxidative Phosphorylation at 2 DAT. *Blue indicates down regulated genes; red indicates up regulated genes and yellow indicates a combination of both—some genes are upregulated, while others are downregulated.


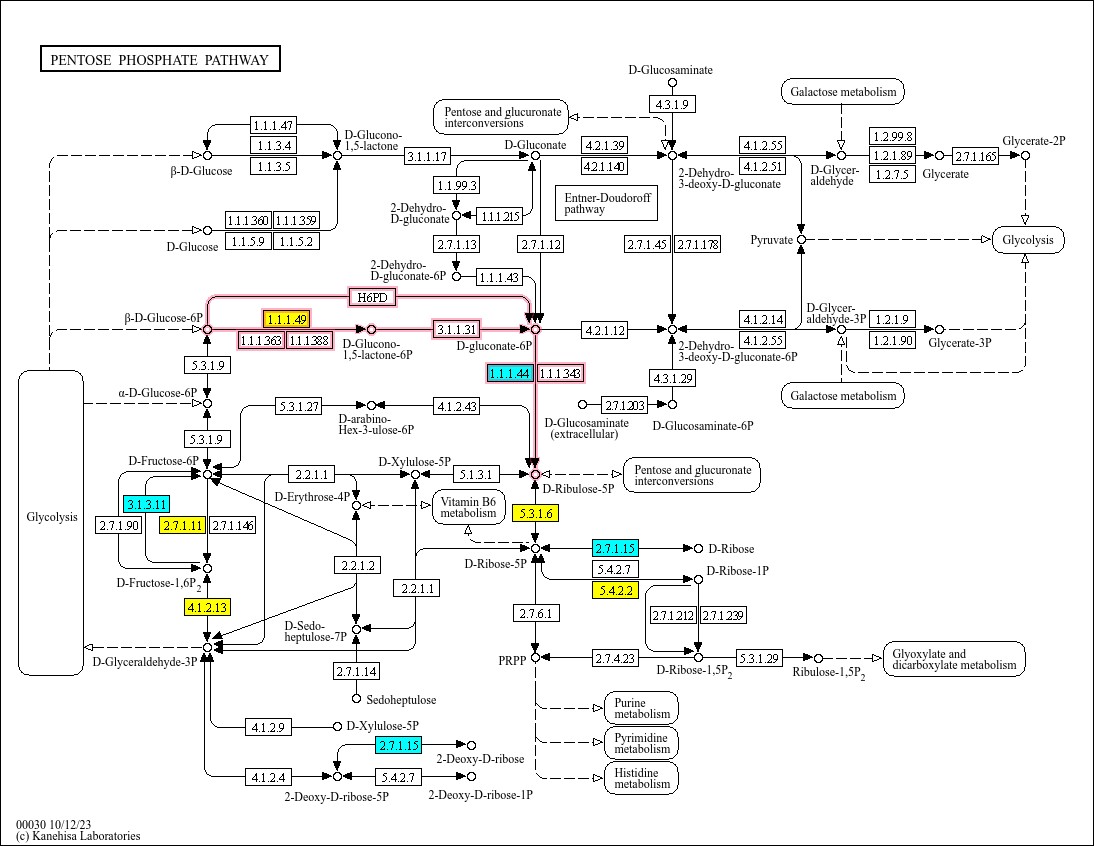


Figure S24. KEGG pathway for Pentose Phosphate Pathway (Oxidative branch) at 1 DAT. *Blue indicates down regulated genes, and yellow indicates a combination of both—some genes are upregulated, while others are downregulated.


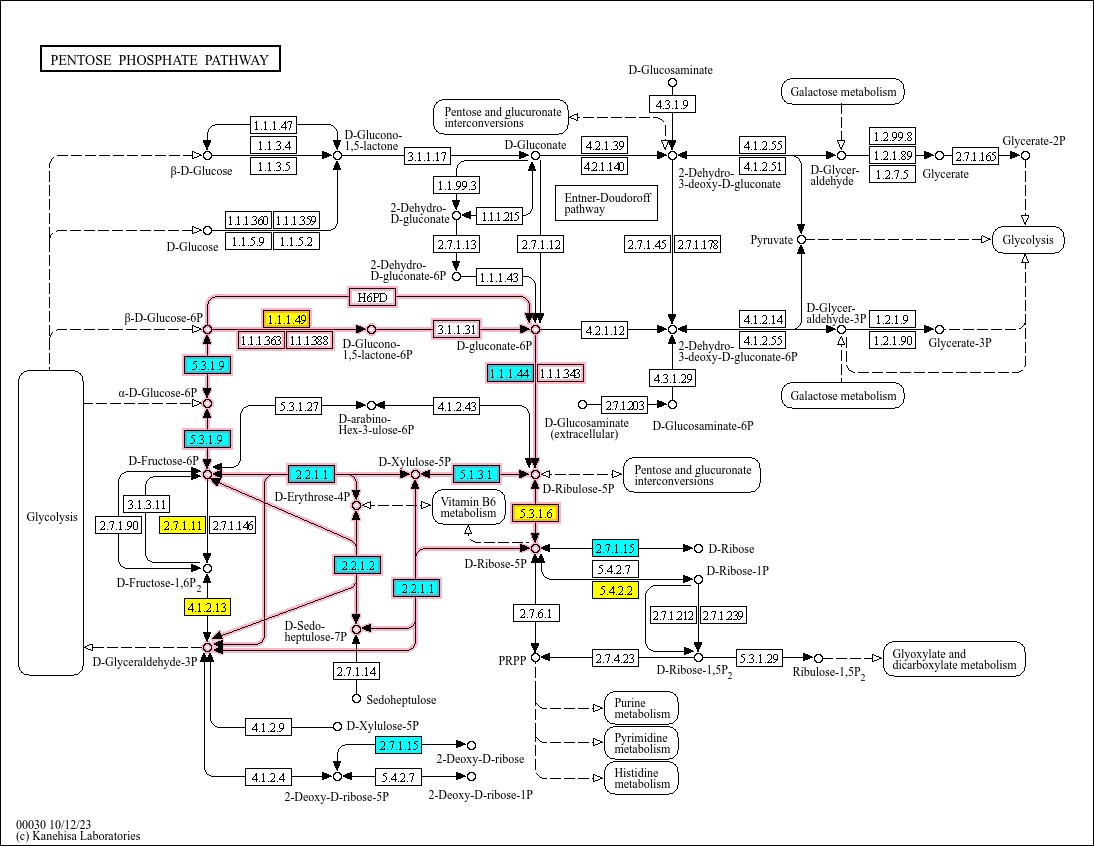


Figure S25. KEGG pathway for Pentose Phosphate Pathway (Oxidative and Non- Oxidative branch) at 2 DAT. *Blue indicates down regulated genes and yellow indicates a combination of both—some genes are upregulated, while others are downregulated.


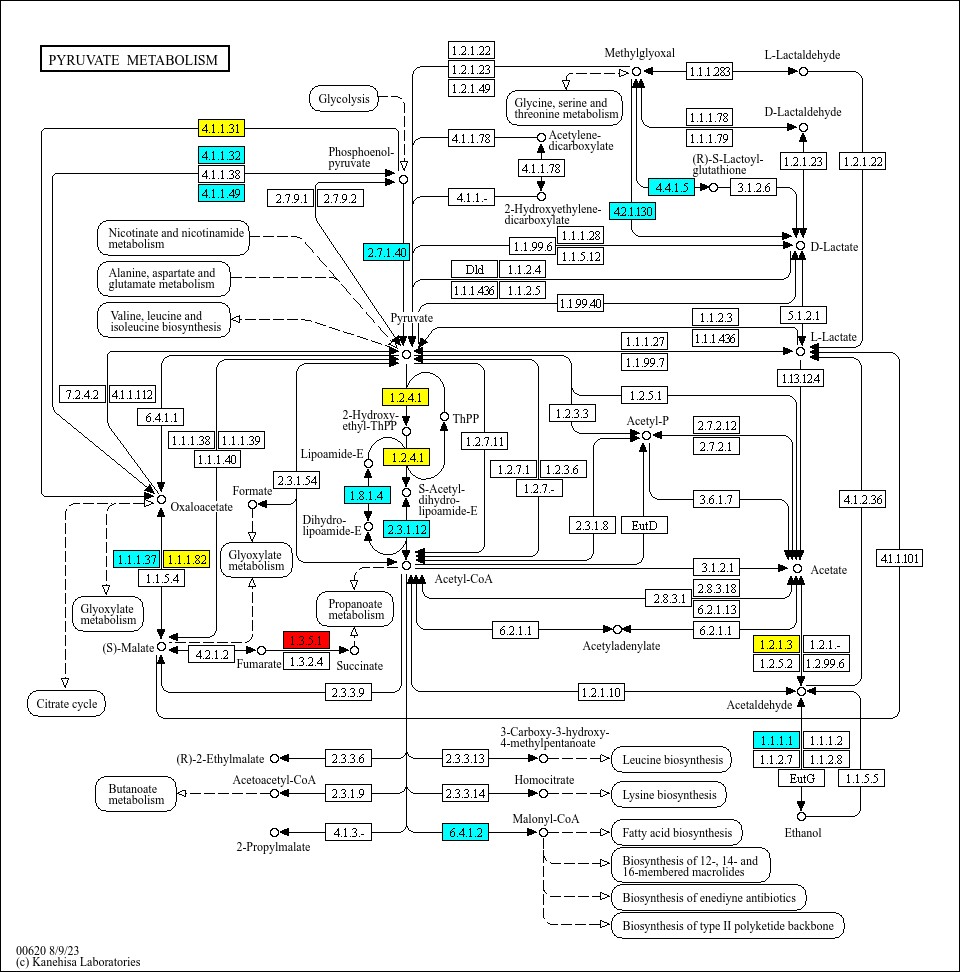
Figure S26. KEGG pathway for Pyruvate Pathway at 2 DAT. *Blue indicates down regulated genes; red indicates up regulated genes and yellow indicates a combination of both—some genes are upregulated, while others are downregulated


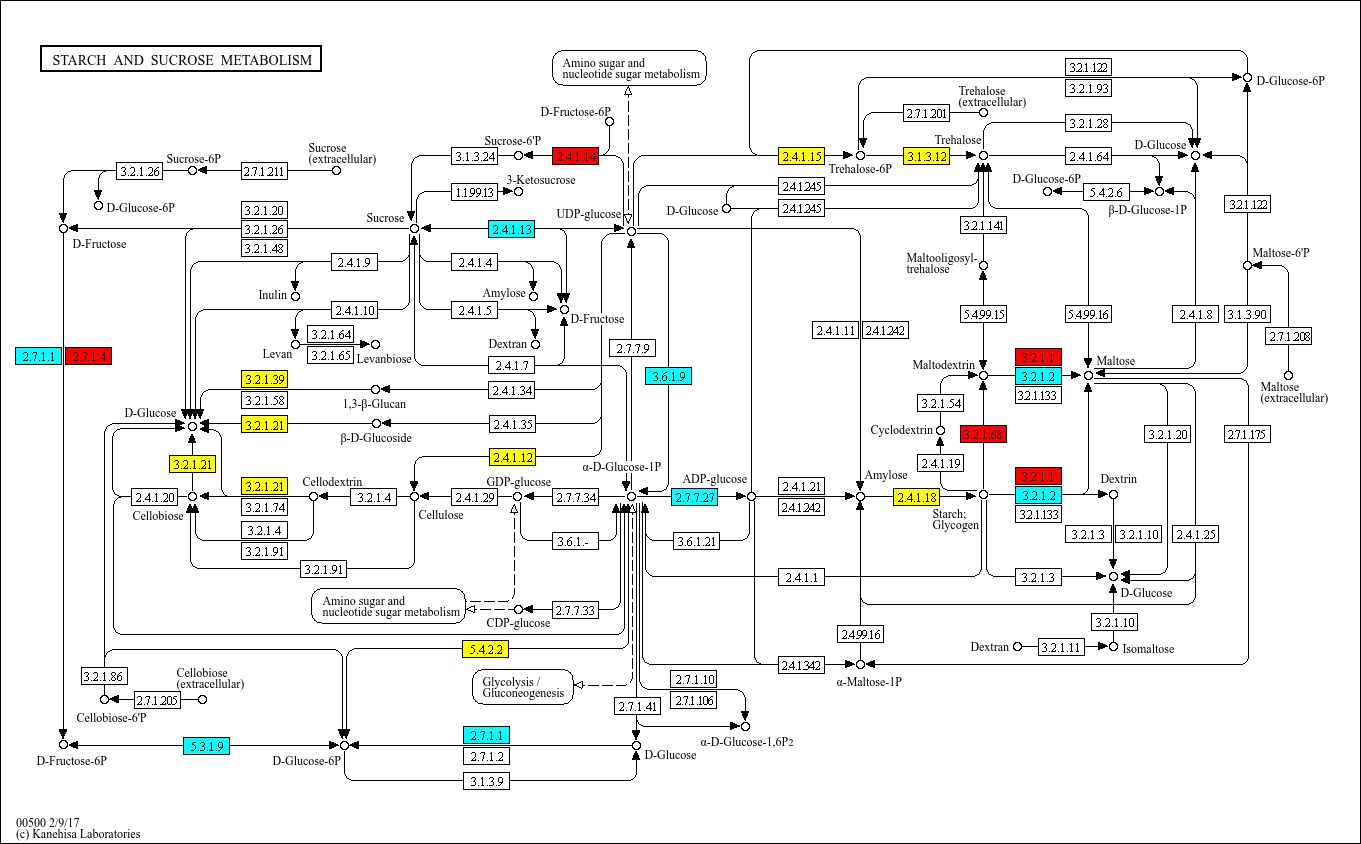
Figure S27. KEGG pathway for Starch and Sucrose metabolism at 2 DAT. *Blue indicates down regulated genes; red indicates up regulated genes, and yellow indicates a combination of both—some genes are upregulated, while others are downregulated.


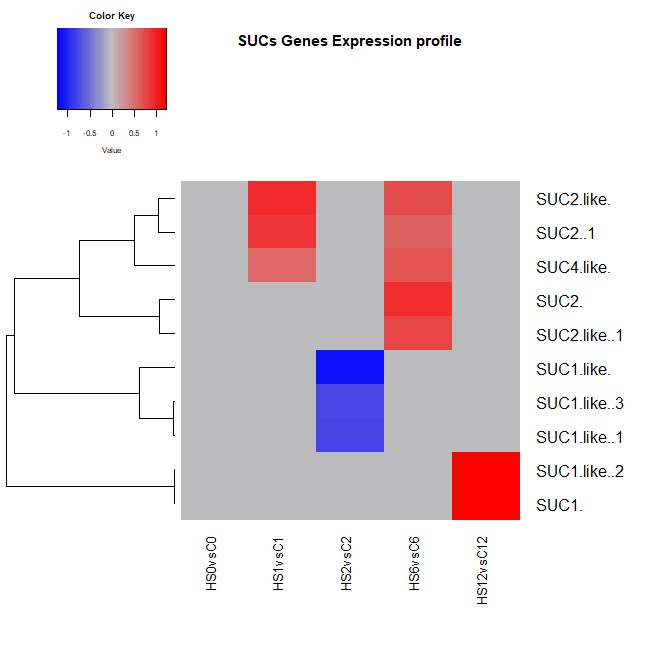


Figure S28. Heatmaps of differentially expressed Sucrose transporters genes (SUCs) at each pairwise comparison. Some transcripts encoding SUCs were found up regulated at 1 DAT and 1 & 7 DOR, while some were down regulated at 2 DAT.

Table S1. Data Quality Summary

| **Sample ID** | **Treatment Day** | **Condition** | **Tissue** | **Library ID** | **Raw reads** | **Raw data** | **Effective(%)** | **Error(%)** | **Q20(%)** | **Q30(%)** | **GC(%)** |
| --- | --- | --- | --- | --- | --- | --- | --- | --- | --- | --- | --- |
| **D0C1** | **0** | **Control** | **Flower Buds** | **R11** | **42546892** | **6.4** | **98.24** | **0.03** | **96.74** | **91.33** | **43.59** |
| **D0C2** | **0** | **Control** | **Flower Buds** | **R12** | **43146158** | **6.5** | **98.22** | **0.03** | **96.35** | **90.41** | **43.11** |
| **D0C3** | **0** | **Control** | **Flower Buds** | **R13** | **48200214** | **7.2** | **98.23** | **0.03** | **96.63** | **91.05** | **43.06** |
| **D0C4** | **0** | **Control** | **Flower Buds** | **R14** | **47204032** | **7.1** | **98.31** | **0.03** | **95.99** | **89.72** | **43.62** |
| **D0C5** | **0** | **Control** | **Flower Buds** | **R15** | **46625810** | **7** | **98.33** | **0.03** | **96.13** | **89.91** | **43.29** |
| **D0HS1** | **0** | **Heat stress** | **Flower Buds** | **R16** | **48823460** | **7.3** | **98.42** | **0.03** | **96.11** | **89.91** | **43.61** |
| **D0HS2** | **0** | **Heat stress** | **Flower Buds** | **R17** | **42361588** | **6.4** | **98.21** | **0.03** | **95.72** | **89.14** | **43.52** |
| **D0HS3** | **0** | **Heat stress** | **Flower Buds** | **R18** | **47472104** | **7.1** | **98.3** | **0.03** | **96.54** | **90.88** | **43.36** |
| **D0HS4** | **0** | **Heat stress** | **Flower Buds** | **R19** | **40668522** | **6.1** | **98.3** | **0.03** | **96.35** | **90.47** | **43.35** |
| **D0HS5** | **0** | **Heat stress** | **Flower Buds** | **R20** | **44058576** | **6.6** | **98.2** | **0.03** | **96.1** | **89.98** | **43.34** |
| **D1C1** | **1** | **Control** | **Flower Buds** | **R21** | **44407298** | **6.7** | **98.53** | **0.03** | **96.46** | **90.65** | **44.78** |
| **D1C2** | **1** | **Control** | **Flower Buds** | **R22** | **42572118** | **6.4** | **98.55** | **0.03** | **96.4** | **90.57** | **45.12** |
| **D1C3** | **1** | **Control** | **Flower Buds** | **R23** | **45707162** | **6.9** | **98.54** | **0.03** | **96.65** | **91.14** | **44.99** |
| **D1C4** | **1** | **Control** | **Flower Buds** | **R24** | **39779066** | **6** | **98.55** | **0.03** | **96.25** | **90.28** | **44.6** |
| **D1C5** | **1** | **Control** | **Flower Buds** | **R25** | **48614394** | **7.3** | **98.21** | **0.03** | **96.3** | **90.47** | **44.96** |
| **D1HS1** | **1** | **Heat stress** | **Flower Buds** | **R26** | **45844218** | **6.9** | **98.37** | **0.03** | **96.43** | **90.59** | **43.9** |
| **D1HS2** | **1** | **Heat stress** | **Flower Buds** | **R27** | **47542486** | **7.1** | **98.21** | **0.03** | **96.07** | **89.82** | **44.54** |
| **D1HS3** | **1** | **Heat stress** | **Flower Buds** | **R28** | **47371258** | **7.1** | **98.46** | **0.03** | **96.52** | **90.88** | **44.61** |
| **D1HS4** | **1** | **Heat stress** | **Flower Buds** | **R29** | **46407612** | **7** | **98.58** | **0.03** | **96.58** | **91.05** | **44.59** |
| **D1HS5** | **1** | **Heat stress** | **Flower Buds** | **R30** | **42705194** | **6.4** | **98.7** | **0.03** | **95.71** | **89.03** | **44.59** |
| **D2C1** | **2** | **Control** | **Flower Buds** | **R31** | **48709190** | **7.3** | **98.45** | **0.03** | **96.39** | **90.54** | **45.57** |
| **D2C2** | **2** | **Control** | **Flower Buds** | **R32** | **50232808** | **7.5** | **98.68** | **0.03** | **96.24** | **90.25** | **45.61** |
| **D2C3** | **2** | **Control** | **Flower Buds** | **R33** | **42394356** | **6.4** | **98.72** | **0.03** | **96.86** | **91.66** | **45.25** |
| **D2C5** | **2** | **Control** | **Flower Buds** | **R35** | **49019624** | **7.4** | **98.26** | **0.03** | **96.62** | **90.98** | **45.2** |
| **D2HS1** | **2** | **Heat stress** | **Flower Buds** | **R36** | **43939042** | **6.6** | **98.46** | **0.03** | **96.65** | **91.08** | **45.12** |
| **D2HS2** | **2** | **Heat stress** | **Flower Buds** | **R37** | **45232502** | **6.8** | **98.6** | **0.03** | **96.83** | **91.45** | **44.77** |
| **D2HS3** | **2** | **Heat stress** | **Flower Buds** | **R38** | **41291490** | **6.2** | **98.43** | **0.03** | **96.6** | **90.94** | **44.93** |
| **D2HS4** | **2** | **Heat stress** | **Flower Buds** | **R39** | **44141240** | **6.6** | **98.52** | **0.03** | **96.57** | **90.87** | **44.94** |
| **D2HS5** | **2** | **Heat stress** | **Flower Buds** | **R40** | **40421822** | **6.1** | **98.54** | **0.03** | **96.48** | **90.68** | **44.36** |
| **D6C1** | **6** | **Control** | **Flower Buds** | **R41** | **45618730** | **6.8** | **98.3** | **0.03** | **96.09** | **89.94** | **43.28** |
| **D6C2** | **6** | **Control** | **Flower Buds** | **R42** | **47279800** | **7.1** | **98.36** | **0.03** | **96.88** | **91.63** | **43.5** |
| **D6C3** | **6** | **Control** | **Flower Buds** | **R43** | **49797014** | **7.5** | **98.42** | **0.03** | **96.2** | **90.13** | **43.04** |
| **D6C4** | **6** | **Control** | **Flower Buds** | **R44** | **48275232** | **7.2** | **98.45** | **0.03** | **95.79** | **89.21** | **43.72** |
| **D6C5** | **6** | **Control** | **Flower Buds** | **R45** | **41275426** | **6.2** | **98.51** | **0.03** | **95.91** | **89.47** | **43.53** |
| **D6HS1** | **6** | **Heat stress** | **Flower Buds** | **R46** | **45090682** | **6.8** | **98.26** | **0.03** | **96.04** | **89.81** | **43.4** |
| **D6HS2** | **6** | **Heat stress** | **Flower Buds** | **R47** | **46214376** | **6.9** | **97.73** | **0.03** | **96.51** | **90.89** | **43.35** |
| **D6HS3** | **6** | **Heat stress** | **Flower Buds** | **R48** | **44791070** | **6.7** | **98.28** | **0.03** | **96.38** | **90.48** | **43.85** |
| **D6HS4** | **6** | **Heat stress** | **Flower Buds** | **R49** | **43154676** | **6.5** | **98.09** | **0.03** | **95.9** | **89.59** | **43.06** |
| **D6HS5** | **6** | **Heat stress** | **Flower Buds** | **R50** | **48451812** | **7.3** | **98.31** | **0.03** | **96.35** | **90.49** | **43.31** |
| **D12C1** | **12** | **Control** | **Flower Buds** | **R51** | **42706732** | **6.4** | **98.61** | **0.03** | **96.19** | **90.2** | **45.62** |
| **D12C2** | **12** | **Control** | **Flower Buds** | **R52** | **45100386** | **6.8** | **98.52** | **0.03** | **97.7** | **93.57** | **45.32** |
| **D12C3** | **12** | **Control** | **Flower Buds** | **R53** | **49761388** | **7.5** | **98.61** | **0.03** | **97.43** | **92.94** | **45.52** |
| **D12C4** | **12** | **Control** | **Flower Buds** | **R54** | **55353976** | **8.3** | **98.67** | **0.03** | **97.45** | **92.98** | **45.8** |
| **D12C5** | **12** | **Control** | **Flower Buds** | **R55** | **43229896** | **6.5** | **98.7** | **0.03** | **97.49** | **93** | **45.51** |
| **D12HS1** | **12** | **Heat stress** | **Flower Buds** | **R56** | **39276294** | **5.9** | **98.68** | **0.03** | **97.39** | **92.84** | **44.61** |
| **D12HS1** | **12** | **Heat stress** | **Flower Buds** | **R57** | **43958400** | **6.6** | **98.8** | **0.03** | **97.61** | **93.31** | **44.4** |
| **D12HS2** | **12** | **Heat stress** | **Flower Buds** | **R58** | **49089438** | **7.4** | **98.67** | **0.03** | **97.43** | **92.92** | **44.69** |
| **D12HS3** | **12** | **Heat stress** | **Flower Buds** | **R59** | **43623028** | **6.5** | **98.71** | **0.03** | **97.54** | **93.2** | **44.56** |
| **D12HS4** | **12** | **Heat stress** | **Flower Buds** | **R60** | **42641344** | **6.4** | **98.62** | **0.03** | **97.47** | **93.04** | **44.27** |

Table S2. PCR timepoints tested per gene

| **Timepoints**  **Genes** | **0 (baseline)** | **1 DAT (24 hrs of HS*)** | **2 DAT (48 hrs of HS)** | **5 DAT (5 days of HS)** | **1 DOR (1 day of recovery)** | **7 DOR (7 days of recovery)** |
| --- | --- | --- | --- | --- | --- | --- |
| **ACTIN** | ✓ | ✓ | ✓ | ✓ | ✓ | ✓ |
| **SS5** | ✓ | X | ✓ | ✓ | ✓ | ✓ |
| **HSP20** | ✓ | X | ✓ | ✓ | ✓ | ✓ |

* HS: Heat Stress

Table S3. List of primer sequences of 3 selected genes and reference gene used for the qt-PCR analysis

| **Primer name** | **Forward primer sequence ((5' to 3')** | **Reverse primer sequence ((5' to 3')** |
| --- | --- | --- |
| **BnaSS5** | GGAGAAAACGCAGGGAAAG | TCAGGCAAACCAAGAACATC |
| **BnaHSP20** | CAAGGAGTATCAGCCAGGTG | TAGAGGGCATCGTCTTTCTC |
| **BnaACTIN** | TGGGTTTGCTGGTGACGAT | TGCCTAGGACGACCAACAATACT |

Table S4.Correlation analysis of the results obtained from RNA-seq and qt-PCR for SS5, HSP20 & BRCA1 genes at different timepoints.

| **Log2 qt-PCR & RNA-seq values** | | | | |
| --- | --- | --- | --- | --- |
|  | **SS5** | | **HSP20** | |
| **Timepoints** | **Qt-PCR** | **RNA-seq** | **Qt-PCR** | **RNA-seq** |
| **HS0vsC0** | 0.141107 | NA* | 6.981295 | 7.6 |
| **HS1vsC1** |  | NA |  | 7.3 |
| **HS2vsC2** | -0.48492 | -1.1 | 8.550429 | 6.1 |
| **HS5vsC5** | 0.163089 |  | 5.673896 |  |
| **HS6vsC6** | 0.814462 | NA | 3.146389 | 6.2 |
| **HS12vsC12** | 0.397126 | NA | 2.671107 | NA |
|  |  |  |  |  |
| **R** | 0.860104 |  | 0.681909 |  |
| **R2** | 0.73978 |  | 0.464999 |  |

NA*: Not DE at this contrast

Table S5. Differential expression data of Sucrose synthesizing enzyme (Sucrose Phosphate Synthase). HS0vsC0, HS1vsC1, HS2vsC2, HS6vsC6 & HS12vsC12 correspond to pairwise comparison of expression between heat stress and control at 0 DAT, 1 DAT, 2 DAT, 1 DOR & 7 DOR respectively.

| **Gene** | **HS0vsC0** | **HS1vsC1** | **HS2vsC2** | **HS6vsC6** | **HS12vsC12** | **Seq. Description** | **Enz. Code** |
| --- | --- | --- | --- | --- | --- | --- | --- |
| BnaA06G0367000WE | NA | 0.69 | NA | 0.9 | 0.7 | Probable sucrose-phosphate synthase 2 | EC:2.4.1.14 |
| BnaC02G0093600WE | 0.5 | 0.76 | 0.77 | NA | NA | Sucrose-phosphate synthase 1-like | EC:2.4.1.14 |
| BnaC07G0352200WE | 0.8 | 1.1 | 1.0 | 1.4 | 0.94 | Probable sucrose-phosphate synthase 2 | EC:2.4.1.14 |
| BnaA02G0084400WE | NA | -0.5 | NA | NA | NA | sucrose-phosphate synthase 1-like | EC:2.4.1.14 |
| BnaA10G0171700WE | -0.52 | NA | NA | NA | NA | sucrose-phosphate synthase 1 | EC:2.4.1.14 |

Table S6. Differential expression data of Sucrose hydrolysing enzymes (Cell Wall Invertase and Sucrose Synthase)

| **Gene** | **HS0vsC0** | **HS1vsC1** | **HS2vsC2** | **HS6vsC6** | **HS12vsC12** | **Seq. Description** | **Enz. Code** |
| --- | --- | --- | --- | --- | --- | --- | --- |
| BnaA03G0187500WE | -1.1 | NA | -1.2 | NA | NA | CWINV4-1; beta-fructofuranosidase | EC:3.2.1.26 |
| BnaC01G0375500WE | NA | NA | NA | 1.7 | NA | CWINV1; beta-fructofuranosidase | EC:3.2.1.26 |
| BnaC03G0139900WE | NA | NA | -1.7 | NA | NA | CWINV4-1; beta-fructofuranosidase | EC:3.2.1.26 |
| BnaC05G0441400WE | 0.9 | NA | NA | 1.5 | NA | CWINV5-2; beta-fructofuranosidase | EC:3.2.1.26 |
| BnaA05G0141800WE | NA | NA | -1.19 | NA | NA | Sucrose synthase 5 | EC:2.4.1.13 |
| BnaA07G0333600WE | NA | NA | -1.45 | NA | NA | Sucrose synthase 6 | EC:2.4.1.13 |
| BnaA09G0016500WE | NA | NA | NA | -1.04 | NA | Sucrose synthase 3 | EC:2.4.1.13 |
| BnaC06G0143400WE | NA | NA | -1.16 | NA | NA | Sucrose synthase 5 | EC:2.4.1.13 |
| BnaA10G0167800WE | NA | NA | NA | NA | -0.8 | Sucrose synthase -1 like | EC:2.4.1.13 |

Table S7. Log2FC of top 30 up regulated genes at 1 DAT, as obtained by DESeq2 pipeline. HS0vsC0, HS1vsC1, HS2vsC2, HS6vsC6 & HS12vsC12 correspond to pairwise comparison of expression between heat stress and control at 0 DAT, 1 DAT, 2 DAT, 1 DOR & 7 DOR respectively.

| **Gene** | **HS0vsC0** | **HS1vsC1** | **HS2vsC2** | **HS6vsC6** | **HS12vsC12** | **Gene description** |
| --- | --- | --- | --- | --- | --- | --- |
| Bnascaffold2770G0000100WE | NA* | 19.39967 | 22.95662 | -22.2903 | NA | Probable ATP-dependent DNA helicase CHR12; K11647 SWI/SNF-related matrix-associated actin-dependent regulator of chromatin subfamily A member 2/4 [EC:3.6.4.-] |
| BnaC03G0236200WE | 10.02693 | 10.83592 | 11.02688 | 5.902732 | NA | HSP22; 22.0 kDa heat shock protein; K13993 HSP20 family protein |
| BnaA06G0312000WE | 7.112808 | 7.788084 | 6.901468 | NA | NA | 23.6 kDa heat shock protein, mitochondrial-like; K13993 HSP20 family protein |
| BnaA01G0068500WE | 7.6943 | 7.31251 | 6.126235 | 6.231749 | NA | 23.6 kDa heat shock protein, mitochondrial-like; K13993 HSP20 family protein |
| BnaC06G0410600WE | 5.130505 | 6.54705 | 7.802509 | 4.717313 | 1.577222 | Developmentally-regulated G-protein 2; K06944 uncharacterized protein |
| BnaC02G0043400WE | 7.053362 | 5.568559 | 6.309569 | 4.491351 | NA | 17.6 kDa class II heat shock protein; K13993 HSP20 family protein |
| BnaC05G0190700WE | 4.426428 | 5.552314 | 5.204933 | 3.399992 | NA | P23; uncharacterized protein OsI_027940-like; K15730 cytosolic prostaglandin-E synthase [EC:5.3.99.3] |
| BnaA06G0177800WE | 6.467447 | 5.331956 | 4.962772 | 5.620651 | NA | BcHSP, BcHSP17.6; 17.4 kDa class I heat shock protein; K13993 HSP20 family protein |
| BnaA09G0429400WE | 4.483177 | 5.324329 | 5.65562 | 4.467147 | NA | P23; uncharacterized protein OsI_027940-like; K15730 cytosolic prostaglandin-E synthase [EC:5.3.99.3] |
| BnaA10G0226400WE | 4.759718 | 5.144102 | 4.922522 | 3.192107 | NA | 17.6 kDa class II heat shock protein-like; K13993 HSP20 family protein |
| BnaA08G0073800WE | 4.06132 | 4.74478 | 4.643646 | 3.274426 | 2.133371 | Zinc finger protein ZPR1-like; K06874 zinc finger protein |
| Bnascaffold3043G0000200WE | 4.538567 | 4.42343 | 4.780174 | 3.693743 | NA | 17.6 kDa class II heat shock protein; K13993 HSP20 family protein (A) |
| BnaC09G0508300WE | 4.005874 | 4.341482 | 3.705517 | 2.137473 | NA | Elongation factor 1-beta 1-like; K03232 elongation factor 1-beta |
| BnaC08G0070600WE | 3.191109 | 4.279984 | 3.789431 | 2.480893 | 1.294645 | Zinc finger protein ZPR1-like; K06874 zinc finger protein |
| BnaC06G0424500WE | 3.258597 | 3.727361 | 3.330465 | 2.497579 | NA | Chaperone protein ClpB1; K03695 ATP-dependent Clp protease ATP-binding subunit ClpB |
| BnaA03G0160300WE | NA | 3.433512 | 2.662603 | NA | NA | Potassium transporter 1; K03549 KUP system potassium uptake protein |
| BnaC06G0380500WE | NA | 3.284288 | 5.346548 | 3.869077 | 5.648205 | Hypothetical protein; K17803 methyltransferase OMS1, mitochondrial [EC:2.1.1.-] |
| BnaC01G0386900WE | 3.477662 | 3.17598 | 3.315892 | 2.845148 | NA | Probable mediator of RNA polymerase II transcription subunit 37c; K03283 heat shock 70kDa protein 1/8 |
| BnaA07G0329100WE | 3.517076 | 3.114972 | 3.111277 | 3.094163 | NA | Developmentally regulated G-protein 2; K06944 uncharacterized protein |
| BnaA07G0341000WE | 2.983552 | 2.990282 | 2.760906 | 2.650909 | NA | Chaperone protein ClpB1; K03695 ATP-dependent Clp protease ATP-binding subunit ClpB |
| BnaC04G0385500WE | 2.891313 | 2.92089 | 3.327077 | 2.293255 | NA | 40S ribosomal protein S9-2; K02997 small subunit ribosomal protein S9e |
| BnaA01G0210500WE | 2.322629 | 2.836025 | 2.120091 | 2.747655 | 1.257562 | Pre-mRNA-splicing factor SLU7-like; K12819 pre-mRNA-processing factor SLU7 |
| BnaA02G0000800WE | NA | 2.833857 | 10.64509 | NA | 11.21154 | Pyridoxal biosynthesis protein PDX1.3; K06215 pyridoxal 5'-phosphate synthase pdxS subunit [EC:4.3.3.6] |
| BnaA05G0117900WE | 2.46685 | 2.792009 | 2.823842 | 1.65756 | NA | Heat shock 70 kDa protein 8-like; K09489 heat shock 70kDa protein 4 |
| BnaA03G0022900WE | 1.939787 | 2.702495 | 1.928843 | 2.251845 | NA | Transcription factor bHLH100-like; K18486 heart-and neural crest derivatives-expressed protein 2 |
| BnaA07G0306600WE | 1.939787 | 2.702495 | 1.928843 | 2.251845 | NA | Transcription factor bHLH100-like; K18486 heart-and neural crest derivatives-expressed protein 2 |
| BnaA05G0366100WE | NA | 2.678283 | 4.275763 | NA | NA | Chaperonin 60 subunit beta 2, chloroplastic; K04077 chaperonin GroEL |
| BnaA03G0344900WE | 2.259242 | 2.673058 | 2.540752 | 1.349036 | NA | Chaperonin 60 subunit beta 2, chloroplastic-like; K04077 chaperonin GroEL |
| BnaA09G0044200WE | 2.428164 | 2.468054 | 2.981328 | 1.303206 | NA | Beta-D-xylosidase 1; K15920 beta-D-xylosidase 4 [EC:3.2.1.37] |
| BnaA04G0180700WE | 2.211296 | 2.410939 | 2.797396 | 2.842365 | NA | 17.6 kDa class I heat shock protein 2-like; K13993 HSP20 family protein |

NA*: Not DE in this contrast

Table S8. Log2FC of top 30 down regulated genes at 1 DAT, as obtained by DESeq2 pipeline. HS0vsC0, HS1vsC1, HS2vsC2, HS6vsC6 & HS12vsC12 correspond to pairwise comparison of expression between heat stress and control at 0 DAT, 1 DAT, 2 DAT, 1 DOR & 7 DOR respectively.

| **Gene** | **HS0vsC0** | **HS1vsC1** | **HS2vsC2** | **HS6vsC6** | **HS12vsC12** | **Gene description** |
| --- | --- | --- | --- | --- | --- | --- |
| BnaA01G0299900WE | NA* | -25.0402 | -24.7672 | NA | NA | Probable ATP-dependent DNA helicase CHR12; K11647 SWI/SNF-related matrix-associated actin-dependent regulator of chromatin subfamily A member 2/4 [EC:3.6.4.-] |
| BnaA02G0390800WE | NA | -24.1049 | NA | NA | NA | 50S ribosomal protein L22-like; K02890 large subunit ribosomal protein L22 |
| Bnascaffold390G0000100WE | NA | -22.8427 | 23.50133 | NA | NA | BTB/POZ and MATH domain-containing protein 2-like; K10523 speckle-type POZ protein |
| BnaA09G0548300WE | NA | -21.0548 | NA | NA | NA | Serine/threonine-protein phosphatase 2A 65 kDa regulatory subunit A beta isoform-like; K03456 serine/threonine-protein phosphatase 2A regulatory subunit A |
| Bnascaffold2041G0000500WE | NA | -10.5063 | NA | NA | -9.63683 | Solute carrier family 2, facilitated glucose transporter member 8-like; K08145 MFS transporter, SP family, solute carrier family 2 (facilitated glucose transporter), member 8 |
| BnaC03G0531800WE | NA | -10.0227 | NA | NA | NA | Asparagine synthetase [glutamine-hydrolyzing]; K01953 asparagine synthase (glutamine-hydrolysing) [EC:6.3.5.4] |
| BnaA05G0410300WE | NA | -7.40007 | 8.067661 | NA | NA | Cell division control protein 48-like; K08900 mitochondrial chaperone BCS1 (A) |
| BnaC03G0161100WE | NA | -5.64143 | -7.23627 | -6.53429 | NA | Bidirectional sugar transporter SWEET9; K15382 solute carrier family 50 (sugar transporter) |
| BnaA08G0122000WE | NA | -5.09401 | NA | NA | NA | Endo-1,4-beta-xylanase C-like; K01181 endo-1,4-beta-xylanase [EC:3.2.1.8] |
| BnaC06G0349400WE | NA | -4.83218 | NA | NA | NA | Peptidase, putative (EC:3.4.21.25); K18443 golgi-specific brefeldin A-resistance guanine nucleotide exchange factor 1 |
| BnaA05G0285000WE | NA | -4.25057 | -3.68042 | -4.24831 | NA | Homocysteine S-methyltransferase 3; K00547 homocysteine S-methyltransferase [EC:2.1.1.10] |
| BnaC02G0269700WE | NA | -3.9799 | NA | NA | NA | Strictosidine synthase 1-like; K01757 strictosidine synthase [EC:4.3.3.2] |
| BnaC03G0440400WE | NA | -3.43342 | NA | NA | NA | Geranylgeranyl diphosphate reductase, chloroplastic; K10960 geranylgeranyl reductase [EC:1.3.1.83] |
| BnaA08G0177000WE | NA | -3.4117 | NA | NA | NA | Glutamyl-tRNA(Gln) amidotransferase subunit A; K02433 aspartyl-tRNA(Asn)/glutamyl-tRNA(Gln) amidotransferase subunit A [EC:6.3.5.6 6.3.5.7] |
| BnaA03G0270900WE | NA | -3.26988 | -3.36832 | -3.13217 | NA | Uncharacterized LOC103858630; K14488 SAUR family protein |
| BnaC05G0336600WE | -2.85089 | -3.01245 | -3.3535 | -2.71611 | NA | Homocysteine S-methyltransferase 3-like; K00547 homocysteine S-methyltransferase [EC:2.1.1.10] |
| BnaC02G0072900WE | NA | -2.95089 | -3.60784 | -3.89519 | NA | Homeobox-leucine zipper protein HDG9-like; K09338 homeobox-leucine zipper protein (A) |
| BnaA03G0328400WE | -3.61265 | -2.90637 | -3.15024 | -3.01336 | NA | Alanine--glyoxylate aminotransferase 2 homolog 3, mitochondrial; K00827 alanine-glyoxylate transaminase / (R)-3-amino-2-methylpropionate-pyruvate transaminase [EC:2.6.1.44 2.6.1.40] |
| BnaA05G0069600WE | -2.44688 | -2.83579 | -2.23355 | -1.87658 | NA | Ribonucleoprotein At2g37220, chloroplastic-like; K11294 nucleolin |
| BnaC01G0410600WE | NA | -2.82246 | -4.1351 | -3.26051 | NA | Cytosolic sulfotransferase 11-like; K01016 estrone sulfotransferase [EC:2.8.2.4] |
| BnaC04G0129500WE | -2.72218 | -2.78587 | NA | -2.45757 | NA | Peroxidase 19; K00430 peroxidase [EC:1.11.1.7] |
| BnaA06G0144700WE | -2.07888 | -2.73372 | -2.42098 | -2.29323 | NA | Auxin:hydrogen symporter, putative; K07088 uncharacterized protein |
| BnaC07G0304700WE | -1.57004 | -2.70645 | -3.10218 | -1.91553 | NA | Bidirectional sugar transporter SWEET4-like; K15382 solute carrier family 50 (sugar transporter) |
| BnaC03G0244200WE | NA | -2.70621 | -2.35369 | -3.11471 | NA | Auxin-responsive protein SAUR36-like; K14488 SAUR family protein |
| BnaC04G0003100WE | -3.12879 | -2.66295 | NA | NA | NA | Probable pectinesterase/pectinesterase inhibitor 20; K01051 pectinesterase [EC:3.1.1.11] |
| BnaA04G0096200WE | NA | -2.55892 | -2.11985 | NA | NA | Proline dehydrogenase 2, mitochondrial; K00318 proline dehydrogenase [EC:1.5.-.-] |
| BnaC02G0359700WE | -2.66622 | -2.5338 | -3.28094 | NA | NA | BAHD acyltransferase DCR; K19747 BAHD acyltransferase [EC:2.3.1.-] |
| BnaA05G0341600WE | NA | -2.43426 | -2.45497 | NA | NA | AAEL008905-PA; K14966 host cell factor |
| BnaC03G0583300WE | NA | -2.37502 | NA | NA | NA | Glutamyl-tRNA(Gln) amidotransferase subunit A; K02433 aspartyl-tRNA(Asn)/glutamyl-tRNA(Gln) amidotransferase subunit A [EC:6.3.5.6 6.3.5.7] |
| BnaC05G0172700WE | -2.1298 | -2.34087 | -2.52748 | -2.03926 | NA | Auxin:hydrogen symporter, putative; K07088 uncharacterized protein |

NA*: Not DE in this contrast

Table S9. Log2FC of top 30 up regulated genes at 2 DAT, as obtained by DESeq2 pipeline. HS0vsC0, HS1vsC1, HS2vsC2, HS6vsC6 & HS12vsC12 correspond to pairwise comparison of expression between heat stress and control at 0 DAT, 1 DAT, 2 DAT, 1 DOR & 7 DOR respectively.

| **Gene** | **HS0vsC** | **HS1vsC1** | **HS2vsC2** | **HS6vsC6** | **HS12vsC12** | **Gene Description** |
| --- | --- | --- | --- | --- | --- | --- |
| BnaC06G0468700WE | NA* | NA | 24.45162 | -23.1045 | NA | Sister chromatid cohesion protein PDS5 homolog A; K11267 sister chromatid cohesion protein PDS5 |
| BnaC02G0000100WE | NA | NA | 23.93091 | NA | 24.17929 | Uncharacterized LOC104816269; K11978 E3 ubiquitin-protein ligase UBR3 [EC:2.3.2.27] |
| Bnascaffold390G0000100WE | NA | -22.8427 | 23.50133 | NA | NA | BTB/POZ and MATH domain-containing protein 2-like; K10523 speckle-type POZ protein |
| Bnascaffold2770G0000100WE | NA | 19.39967 | 22.95662 | -22.2903 | NA | Probable ATP-dependent DNA helicase CHR12; K11647 SWI/SNF-related matrix-associated actin-dependent regulator of chromatin subfamily A member 2/4 [EC:3.6.4.-] |
| BnaC02G0211000WE | NA | NA | 21.93849 | -21.602 | NA | Putative caffeoyl-CoA O-methyltransferase At1g67980; K00588 caffeoyl-CoA O-methyltransferase [EC:2.1.1.104] |
| BnaA02G0169400WE | NA | NA | 21.93396 | NA | NA | Putative caffeoyl-CoA O-methyltransferase At1g67980; K00588 caffeoyl-CoA O-methyltransferase [EC:2.1.1.104] |
| BnaA05G0284500WE | NA | NA | 21.52973 | NA | NA | Hypothetical protein; K00128 aldehyde dehydrogenase (NAD+) [EC:1.2.1.3] |
| BnaC03G0236200WE | 10.02693 | 10.83592 | 11.02688 | 5.902732 | NA | HSP22; 22.0 kDa heat shock protein; K13993 HSP20 family protein |
| BnaA02G0000800WE | NA | 2.833857 | 10.64509 | NA | 11.21154 | Pyridoxal biosynthesis protein PDX1.3; K06215 pyridoxal 5'-phosphate synthase pdxS subunit [EC:4.3.3.6] |
| Bnascaffold1692G0000200WE | NA | NA | 10.20429 | NA | NA | Nucleolar protein 56-like; K14564 nucleolar protein 56 |
| BnaA05G0410300WE | NA | -7.40007 | 8.067661 | NA | NA | Cell division control protein 48-like; K08900 mitochondrial chaperone BCS1 |
| BnaA03G0518500WE | NA | NA | 7.944113 | NA | NA | Spermidine coumaroyl CoA acyltransferase-like; K15400 omega-hydroxypalmitate O-feruloyl transferase [EC:2.3.1.188] |
| BnaC06G0410600WE | 5.130505 | 6.54705 | 7.802509 | 4.717313 | 1.577222 | Developmentally regulated G-protein 2; K06944 uncharacterized protein |
| BnaA05G0186500WE | NA | NA | 7.735252 | NA | NA | Aldehyde dehydrogenase family 3 member H1-like; K00128 aldehyde dehydrogenase (NAD+) [EC:1.2.1.3] |
| BnaA06G0312000WE | 7.112808 | 7.788084 | 6.901468 | NA | NA | 23.6 kDa heat shock protein, mitochondrial-like; K13993 HSP20 family protein |
| BnaC02G0043400WE | 7.053362 | 5.568559 | 6.309569 | 4.491351 | NA | 17.6 kDa class II heat shock protein; K13993 HSP20 family protein |
| BnaA01G0068500WE | 7.6943 | 7.31251 | 6.126235 | 6.231749 | NA | 23.6 kDa heat shock protein, mitochondrial-like; K13993 HSP20 family protein |
| BnaA09G0429400WE | 4.483177 | 5.324329 | 5.65562 | 4.467147 | NA | P23; uncharacterized protein OsI_027940-like; K15730 cytosolic prostaglandin-E synthase [EC:5.3.99.3] |
| BnaC06G0380500WE | NA | 3.284288 | 5.346548 | 3.869077 | 5.648205 | Hypothetical protein; K17803 methyltransferase OMS1, mitochondrial [EC:2.1.1.-] |
| BnaC05G0190700WE | 4.426428 | 5.552314 | 5.204933 | 3.399992 | NA | P23; uncharacterized protein OsI_027940-like; K15730 cytosolic prostaglandin-E synthase [EC:5.3.99.3] |
| BnaC03G0524800WE | 5.407274 | NA | 5.085613 | 2.727258 | NA | 17.6 kDa class I heat shock protein 3-like; K13993 HSP20 family protein |
| BnaA06G0177800WE | 6.467447 | 5.331956 | 4.962772 | 5.620651 | NA | BcHSP, BcHSP17.6; 17.4 kDa class I heat shock protein; K13993 HSP20 family protein |
| BnaA10G0226400WE | 4.759718 | 5.144102 | 4.922522 | 3.192107 | NA | 17.6 kDa class II heat shock protein-like; K13993 HSP20 family protein |
| Bnascaffold3043G0000200WE | 4.538567 | 4.42343 | 4.780174 | 3.693743 | NA | 17.6 kDa class II heat shock protein; K13993 HSP20 family protein (A) |
| BnaA04G0104000WE | 6.423794 | NA | 4.726792 | 5.495205 | NA | 40S ribosomal protein S9-2-like; K02997 small subunit ribosomal protein S9e (A) |
| BnaA08G0073800WE | 4.06132 | 4.74478 | 4.643646 | 3.274426 | 2.133371 | Zinc finger protein ZPR1-like; K06874 zinc finger protein |
| BnaC03G0057000WE | 6.374908 | NA | 4.491543 | 6.206712 | NA | 18.1 kDa class I heat shock protein-like; K13993 HSP20 family protein |
| BnaA05G0366100WE | NA | 2.678283 | 4.275763 | NA | NA | Chaperonin 60 subunit beta 2, chloroplastic; K04077 chaperonin GroEL |
| BnaA04G0089500WE | 2.939984 | NA | 4.269828 | NA | NA | 15.7 kDa heat shock protein, peroxisomal; K13993 HSP20 family protein |
| BnaC08G0285900WE | NA | NA | 4.237486 | NA | NA | Aquaporin PIP2-1-like; K09872 aquaporin PIP |

NA*: Not DE in this contrast

Table S10. Log2FC of top 30 down regulated genes at 2 DAT, as obtained by DESeq2 pipeline. HS0vsC0, HS1vsC1, HS2vsC2, HS6vsC6 & HS12vsC12 correspond to pairwise comparison of expression between heat stress and control at 0 DAT, 1 DAT, 2 DAT, 1 DOR & 7 DOR respectively.

| **Gene** | **HS0vsC0** | **HS1vsC1** | **HS2vsC2** | **HS6vsC6** | **HS12vsC12** | **Gene Description** |
| --- | --- | --- | --- | --- | --- | --- |
| BnaA01G0299900WE | NA* | -25.0402 | -24.7672 | NA | NA | Probable ATP-dependent DNA helicase CHR12; K11647 SWI/SNF-related matrix-associated actin-dependent regulator of chromatin subfamily A member 2/4 [EC:3.6.4.-] |
| BnaC06G0162800WE | 23.16202 | NA | -22.4002 | NA | NA | Hypothetical protein; K10352 myosin heavy chain |
| BnaC03G0161100WE | NA | -5.64143 | -7.23627 | -6.53429 | NA | Bidirectional sugar transporter SWEET9; K15382 solute carrier family 50 (sugar transporter) |
| BnaA08G0139800WE | NA | NA | -4.68598 | NA | NA | Geranylgeranyl diphosphate reductase, chloroplastic; K10960 geranylgeranyl reductase [EC:1.3.1.83] |
| BnaA10G0021500WE | NA | NA | -4.38041 | NA | NA | Secretory carrier-associated membrane protein 2-like; K19995 secretory carrier-associated membrane protein |
| BnaA03G0204300WE | NA | NA | -4.18928 | NA | NA | Bra000116, SWEET9; bidirectional sugar transporter SWEET9; K15382 solute carrier family 50 (sugar transporter) |
| BnaC01G0410600WE | NA | -2.82246 | -4.1351 | -3.26051 | NA | Cytosolic sulfotransferase 11-like; K01016 estrone sulfotransferase [EC:2.8.2.4] |
| BnaC05G0020800WE | NA | NA | -4.11721 | -4.93054 | NA | Secretory carrier-associated membrane protein 2; K19995 secretory carrier-associated membrane protein |
| BnaC03G0671900WE | NA | NA | -3.96775 | -2.74931 | NA | Geranylgeranyl diphosphate reductase, chloroplastic; K10960 geranylgeranyl reductase [EC:1.3.1.83] |
| BnaC09G0341100WE | NA | NA | -3.93873 | -3.46231 | NA | Flavone 3'-O-methyltransferase 1-like; K13066 caffeic acid 3-O-methyltransferase [EC:2.1.1.68] |
| BnaC08G0470200WE | NA | NA | -3.76408 | NA | NA | Probable fructose-bisphosphate aldolase 3, chloroplastic; K01623 fructose-bisphosphate aldolase, class I [EC:4.1.2.13] |
| BnaA06G0424600WE | NA | NA | -3.72984 | NA | NA | 1,8-cineole synthase 1, chloroplastic-like; K07385 1,8-cineole synthase [EC:4.2.3.108] |
| BnaA03G0462900WE | NA | NA | -3.70364 | -5.34377 | NA | CYP71A13; K11868 indoleacetaldoxime dehydratase [EC:4.99.1.6] |
| BnaC09G0263300WE | NA | NA | -3.69454 | NA | NA | auxin-responsive protein SAUR36-like; K14488 SAUR family protein (A) |
| BnaA05G0285000WE | NA | -4.25057 | -3.68042 | -4.24831 | NA | Homocysteine S-methyltransferase 3; K00547 homocysteine S-methyltransferase [EC:2.1.1.10] |
| BnaC02G0072900WE | NA | -2.95089 | -3.60784 | -3.89519 | NA | Homeobox-leucine zipper protein HDG9-like; K09338 homeobox-leucine zipper protein |
| BnaC06G0215000WE | -5.08388 | NA | -3.4853 | NA | NA | Probable mitochondrial chaperone BCS1-B; K08900 mitochondrial chaperone BCS1 |
| BnaA05G0348800WE | NA | NA | -3.43047 | -3.97975 | NA | NAC domain-containing protein 89-like; K12581 CCR4-NOT transcription complex subunit 7/8 (A) |
| BnaA03G0270900WE | NA | -3.26988 | -3.36832 | -3.13217 | NA | uncharacterized LOC103858630; K14488 SAUR family protein (A) |
| BnaC05G0336600WE | -2.85089 | -3.01245 | -3.3535 | -2.71611 | NA | Homocysteine S-methyltransferase 3-like; K00547 homocysteine S-methyltransferase [EC:2.1.1.10] |
| BnaA02G0003400WE | -2.32333 | NA | -3.32554 | NA | NA | ATP-dependent zinc metalloprotease FTSH 10, mitochondrial; K08956 AFG3 family protein [EC:3.4.24.-] (A) |
| BnaA08G0141600WE | NA | NA | -3.31656 | -6.37331 | NA | Venom phosphodiesterase 2-like; K01513 ectonucleotide pyrophosphatase/phosphodiesterase family member 1/3 [EC:3.1.4.1 3.6.1.9] |
| BnaC02G0359700WE | -2.66622 | -2.5338 | -3.28094 | NA | NA | BAHD acyltransferase DCR; K19747 BAHD acyltransferase [EC:2.3.1.-] |
| BnaA03G0328400WE | -3.61265 | -2.90637 | -3.15024 | -3.01336 | NA | Alanine--glyoxylate aminotransferase 2 homolog 3, mitochondrial; K00827 alanine-glyoxylate transaminase / (R)-3-amino-2-methylpropionate-pyruvate transaminase [EC:2.6.1.44 2.6.1.40] |
| BnaC07G0304700WE | -1.57004 | -2.70645 | -3.10218 | -1.91553 | NA | Bidirectional sugar transporter SWEET4-like; K15382 solute carrier family 50 (sugar transporter) |
| BnaC06G0297300WE | NA | NA | -3.06469 | NA | NA | Uncharacterized LOC101250545; K11426 SET and MYND domain-containing protein |
| BnaC03G0258400WE | NA | NA | -2.99598 | NA | NA | Glutathione S-transferase F3-like; K00799 glutathione S-transferase [EC:2.5.1.18] |
| BnaC04G0492800WE | NA | -1.58127 | -2.95436 | NA | NA | BrNIT1, NIT-T2; nitrilase 2-like; K01501 nitrilase [EC:3.5.5.1] |
| BnaC01G0191600WE | NA | NA | -2.95091 | -5.16291 | NA | Caffeic acid 3-O-methyltransferase-like; K13066 caffeic acid 3-O-methyltransferase [EC:2.1.1.68] |
| BnaA10G0150800WE | NA | -2.02317 | -2.86033 | NA | NA | Laccase-17-like; K05909 laccase [EC:1.10.3.2] |

NA*: Not DE in this contrast
